# Contrasting salinity regimes reshape microbe–DOM coupling and reduce recalcitrant dissolved organic carbon preservation in a salt lake

**DOI:** 10.1093/ismejo/wrag107

**Published:** 2026-05-07

**Authors:** Xiding Wang, Yang Liu, Xinyue Yang, Ruikai Zhang, Xudong Liu, Fangru Nan, Qi Liu, Junping Lv, Jia Feng, Shulian Xie

**Affiliations:** Shanxi Key Laboratory for Research and Development of Regional Plants, School of Life Science, Shanxi University, Taiyuan 030006, China; Shanxi Key Laboratory for Research and Development of Regional Plants, School of Life Science, Shanxi University, Taiyuan 030006, China; Shanxi Key Laboratory for Research and Development of Regional Plants, School of Life Science, Shanxi University, Taiyuan 030006, China; Shanxi Key Laboratory for Research and Development of Regional Plants, School of Life Science, Shanxi University, Taiyuan 030006, China; Shanxi Key Laboratory for Research and Development of Regional Plants, School of Life Science, Shanxi University, Taiyuan 030006, China; Shanxi Key Laboratory for Research and Development of Regional Plants, School of Life Science, Shanxi University, Taiyuan 030006, China; Shanxi Key Laboratory for Research and Development of Regional Plants, School of Life Science, Shanxi University, Taiyuan 030006, China; Shanxi Key Laboratory for Research and Development of Regional Plants, School of Life Science, Shanxi University, Taiyuan 030006, China; Shanxi Key Laboratory for Research and Development of Regional Plants, School of Life Science, Shanxi University, Taiyuan 030006, China; Shanxi Key Laboratory for Research and Development of Regional Plants, School of Life Science, Shanxi University, Taiyuan 030006, China

**Keywords:** salt lakes, dissolved organic carbon, recalcitrant dissolved organic carbon, salinity, microbial communities

## Abstract

Salt lakes account for nearly half of the world’s inland water area and play an irreplaceable role as “carbon conversion and stabilization factories,” making substantial contributions to the global carbon cycle. Central to this function is the transformation of dissolved organic carbon (DOC) and its accumulation into recalcitrant dissolved organic carbon (RDOC), which together underpin internal carbon processing in these systems. However, the pathways through which DOC is converted to RDOC in salt lakes, and how these pathways are shaped by salinity and microbial communities, remain poorly resolved. Here, using Yuncheng Salt Lake as a within-lake system, we combined field-based *in situ* characterization with long-term incubation experiments to examine how contrasting salinity regimes were associated with microbial and dissolved organic matter (DOM) variation. Higher salinity was associated with reduced bacterial and dissolved organic matter diversity, stronger deterministic bacterial assembly, and a restructured bacteria–DOM association network. Under the standardized nutrient-replete incubation conditions used here, samples from the higher-salinity regime exhibited higher biodegradable DOC, lower RDOC preservation, and greater overall DOC loss over the 100-day experimental timescale. Salinity-related differences in microbial community composition, metabolomic profiles, and dissolved organic matter characteristics were closely associated with variation in RDOC dynamics, suggesting that these carbon-processing differences were accompanied by coordinated microbial and metabolic reorganization. Together, these results provide process-relevant, condition-specific evidence that contrasting salinity regimes within Yuncheng Salt Lake were associated with differences in microbe–DOM coupling and in DOC/RDOC outcomes.

## Graphical Abstract

**Graphical Abstract:**
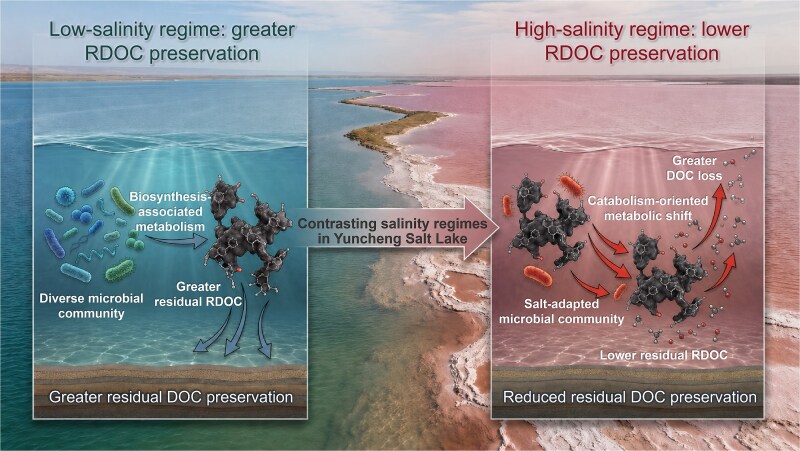

## Introduction

Inland saline lakes, which occupy ~44% of the world’s inland water area [1], represent a dynamic, and often overlooked, interface in the global carbon cycle because of their distinctive hydrogeochemical features, including high salinity, intense evaporation, and frequent wet–dry cycles [2, 3]. Carbon-cycle research has traditionally focused on forests, oceans, and freshwater systems; consequently, the sink/source function of salt lakes has been underestimated, in part because these lakes are spatially dispersed and are subjected to environmentally “extreme” conditions [4, 5]. However, endorheic basins have stored ~174 Pg of carbon since the Last Glacial Maximum, accounting for ~15%–20% of the global inland sedimentary carbon pool [4, 6], but they also contribute an estimated ~18%–50% of lake-derived CO_2_ emissions [7–9]. This delicate balance is increasingly being perturbed by rapid salinization and desiccation driven by climate change and human activities, which threaten to destabilize stored carbon and alter lake biogeochemical function [10].

A key to understanding this balance is the behavior of dissolved organic carbon (DOC). Although oceans contain the largest dissolved organic matter (DOM) reservoir (~662 Pg C) [11], inland saline lakes often exhibit disproportionately high DOC concentrations, frequently reaching thousands of milligrams of carbon per liter, owing to long hydraulic residence times and evaporative concentration [12–14]. The fate of this large carbon pool is governed by interacting abiotic and microbial processes that collectively operate as a “biogeochemical reactor.” High salinity and strong ultraviolet radiation promote photochemical transformation and mineral-organic interactions [13], whereas salinity-adapted microbial communities, via mechanisms such as the microbial carbon pump, selectively consume labile DOC and convert a fraction into recalcitrant DOC (RDOC) [15]. This coupling of abiotic condensation and biological transformation can strongly influence the balance between carbon remineralization and carbon preservation in saline lakes and can also foster the accumulation of long-lived RDOC, thereby shaping their carbon sequestration potential [16].

In this framework, salinity acts as a strong environmental filter that shapes microbial diversity, community composition, and functional traits. Bacterial assemblages typically shift from diverse, mixed communities at lower salinity to more specialized halotolerant and halophilic taxa under hypersaline conditions [17–19]. In parallel, salinity modifies DOM through physicochemical processes (e.g. flocculation, solubility shifts, and ionic complexation) and biologically mediated transformations, resulting in systematic changes in molecular composition, elemental ratios, and optical properties [20, 21]. Recent evidence suggests that increasing salinity tends to replace aged, aromatic DOM with smaller, more oxidized, microbially derived compounds, and this is often accompanied by the loss of aliphatic and carboxyl-rich alicyclic molecules (CRAMs) and the enrichment of oxygen-rich DOM constituents [13]. Microbial community attributes frequently explain more variation in DOM composition than abiotic factors alone, highlighting that DOM transformation in saline lakes is tightly regulated by microbial processes [22].

Although many studies have characterized the spatiotemporal patterns of DOM and associated microbial communities in salt lakes, our understanding of how salinity is linked to DOC degradation and RDOC preservation remains limited. In particular, it is still unclear whether salinity-associated differences in carbon-processing outcomes are more closely related to microbial metabolic reorganization than to taxonomic turnover alone. Here, using Yuncheng Salt Lake as a within-lake comparison system with contrasting low- and high-salinity basins and three representative habitats (water, the water–sediment interface, and sediments), we aimed to (i) determine how contrasting salinity regimes are associated with DOC degradation and its partitioning between biodegradable DOC (BDOC) and RDOC; (ii) evaluate whether these carbon-processing differences are linked more strongly to microbial metabolic variation than to changes in community composition alone; and (iii) use bacterial community, DOM assembly, and bacteria–DOM association analyses to provide ecological context for salinity-associated restructuring of the system. By integrating Fourier-transform ion cyclotron resonance mass spectrometry (FT-ICR MS), excitation–emission matrix spectroscopy coupled with parallel factor analysis (EEM-PARAFAC), 16S rRNA gene sequencing, metabolomics, *in situ* characterization, and long-term laboratory incubation, we sought to link salinity-associated ecological and chemical reorganization with variation in DOC degradation and RDOC preservation. A better process-based understanding of these relationships is important for improving global carbon budget estimates, interpreting carbon-cycle variability in paleoclimate records, and projecting carbon-cycle feedbacks under future climate change.

## Materials and methods

### Site description and sample collection

Yuncheng Salt Lake [23], often referred to as the “Dead Sea of China,” is located on the southern outskirts of Yuncheng City, Shanxi Province, eastern China (34°54′00″–35°04′00″N, 110°07′30″–110°50′00″E) (Supplementary Fig. S1). It is the world’s third-largest natural inland sodium sulfate lake. The brine displays two distinct salinity regimes: a low-salinity zone (~25 ppt) and a high-salinity zone (~80 ppt) [24]. Sampling was conducted in autumn, beginning on 22 October 2024, and all sites were sampled within a 2-day period to minimize short-term temporal variation in DOM composition and microbial communities. To characterize the system comprehensively and ensure that our conclusions were not restricted to a single matrix, we collected three types of samples from each salinity regime: water, the water–sediment interface, and surface sediments (~0–15 cm depth). These regimes provide a within-lake spatial contrast under a shared regional setting and are treated here as two contrasting salinity environments rather than as a temporal record of progressive salinization. Five sampling sites were established in the low-salinity zone and five in the high-salinity zone. At each site, all three media were sampled in parallel, yielding 30 samples in total. For the water, 2 l of lake water was collected in sterile polypropylene bottles. Water–sediment interface material (the upper mixed, flocculent layer at the lake bottom) was gently scooped with sterile spatulas and transferred to sterile containers (250 g wet weight per site). Surface sediments were collected using a stainless-steel corer, and the 0–15 cm section was retained (500 g wet weight per site). This depth interval may integrate multiple microscale redox layers within the surface sediment. Therefore, the measured DOM and microbial characteristics represent depth-integrated near-surface sediment signatures rather than layer-specific redox-resolved profiles. Such integration may smooth fine-scale vertical heterogeneity, but it provides a standardized bulk surface-sediment comparison across sites and salinity regimes. Immediately after collection, samples were placed in insulated ice boxes maintained at 4°C, transported to the laboratory within 6 h, and processed upon arrival. For microbial community analysis, 500 ml of each water sample was filtered through a sterile 0.22 μm membrane filter. For interface and sediment samples, 10 g subsamples were aliquoted into sterile 50-ml centrifuge tubes. Filters and solid subsamples designated for DNA analysis were snap frozen after preprocessing and stored at −80°C. DNA was extracted within 24 h of sampling, and the extracted DNA was stored at −80°C until PCR amplification and sequencing.

### Physicochemical parameters

*In situ* water parameters, including temperature, dissolved oxygen (DO), pH, salinity (SAL), total dissolved solids (TDS), and specific conductance (SPC), were measured at each site using a YSI Pro Plus multiparameter water-quality meter (YSI Inc., Yellow Springs, OH, USA). Concentrations of ammonium nitrogen (NH_4_^+^-N), total nitrogen (TN), total phosphorus (TP), and chemical oxygen demand (COD) in water samples were quantified in the laboratory following standard analytical methods [25]. Dissolved organic carbon (DOC) was measured after filtration through pre-combusted GF/F filters (Whatman, UK). Filtrates were acidified to pH <2 with HCl and analyzed using a total organic carbon analyzer (TOC-L; Shimadzu, Japan) operated in high-temperature catalytic oxidation mode. Each sample was analyzed in triplicate, with instrument blanks and certified reference standards included for quality assurance. For sediment samples, pH and SPC were measured in a 1:2.5 (w/v) sediment-to-deionized water slurry using a calibrated pH/EC meter. Total organic carbon (TOC) was determined using a TOC analyzer after removal of inorganic carbon with dilute HCl. Total silicon (TSi), total phosphorus (TP), total iron (TFe), and total aluminum (TAl) were quantified after mixed-acid digestion (HNO_3_–HF–HClO_4_) using inductively coupled plasma optical emission spectrometry. Sediment DOC was determined following aqueous extraction [26]. Briefly, 3 g of air-dried, homogenized sediment was mixed with 30 ml of ultrapure water and shaken at 150 rpm for 2 h to extract water-soluble organic components. The slurry was then centrifuged at 3000×*g* for 20 min, and the supernatant was collected and filtered through pre-rinsed 0.45-μm membrane filters. DOC in the filtrates was measured using a TOC-L analyzer (Shimadzu). A portion of the clarified extract was used immediately for DOC quantification, and the remaining volume was reserved for subsequent solid-phase extraction of DOM for FT-ICR MS and EEM-PARAFAC analyses. The physicochemical parameters are summarized in Tables S1, S2 and S3.

### DNA extraction, amplification, and sequencing

Total DNA was extracted from all samples using an E.Z.N.A. Mag-Bind Soil DNA Kit (Omega, M5635-02, USA) following the manufacturer’s protocol. DNA concentration and purity were assessed with a Qubit fluorometer to confirm that sufficient, high-quality DNA was obtained for sequencing. Bacterial 16S rRNA genes were amplified from the V3–V4 region using the primer pair 341F (CCTACGGGNGGCWGCAG) and 806R (GACTACHVGGGTATCTAATCC). Purified amplicons were pooled and sequenced on a HiSeq System (Illumina) at BGI (Shenzhen, China). Paired-end reads were quality-filtered and merged using PEAR, and downstream processing was performed with USEARCH. After removing chimeras and singletons, sequences were clustered into operational taxonomic units (OTUs) at 97% similarity. Representative OTU sequences were taxonomically assigned using the RDP database (v11.4). The resulting OTU table was used for all subsequent diversity and community analyses. Additional details on PCR conditions and sequence processing are provided in Supplementary Methods 1.

### E‌EM-PARAFAC analysis

Fluorescence excitation-emission matrices (EEMs) were collected for all DOM samples using a spectrofluorometer (F-7100; Hitachi, Tokyo, Japan). Excitation wavelengths were scanned from 200 to 450 nm, and emission spectra were recorded from 230 to 600 nm at a constant scan rate of 30 000 nm min^−1^. Both excitation and emission slit widths were set to 5 nm per the manufacturer’s recommendations. Ultrapure water blanks were measured under identical settings and subtracted from sample spectra to correct for instrument background and Raman signals. Inner-filter effects and Rayleigh/Raman scattering were further reduced using established correction procedures and the instrument software [27].

Corrected EEMs were decomposed by parallel factor analysis (PARAFAC) using the DOMFluor toolbox in MATLAB 2023b (MathWorks, USA). Model robustness was evaluated via split-half validation and inspection of residuals. In addition to PARAFAC components, standard fluorescence indices were calculated, including the fluorescence index (FI), humification index (HIX), biological index (BIX), and the β/α ratio, based on characteristic regions of the excitation–emission space [28–30].

### FT-ICR MS analysis

For molecular-level characterization, DOM was isolated by solid-phase extraction (SPE) and analyzed using FT-ICR MS. All glassware that contacted samples was rigorously pre-cleaned by sequential acid washing, rinsed with ultrapure water, and underwent high-temperature combustion to minimize organic contamination. Filtered DOM solutions (typically 100 ml per sample) were collected in acid-cleaned amber vials and stored at −20°C in the dark until extraction. To minimize interference from inorganic salts during electrospray ionization, samples were desalted prior to mass spectrometry. SPE was performed using Bond Elut PPL cartridges (200 mg, 3 ml; Agilent Technologies). Cartridges were conditioned with liquid chromatography-mass spectrometry (LC–MS)-grade methanol and then equilibrated with acidified (pH 2) ultrapure water before sample loading. Acidified DOM filtrates were loaded onto the cartridges by gravity flow through Teflon tubing. After loading, cartridges were gently dried under a stream of high-purity nitrogen in the dark, and retained DOM was eluted with LC–MS-grade methanol. Eluates were stored at −20°C until FT-ICR MS analysis [31].

Mass spectra were acquired on a 7.0-T Solarix 2XR FT-ICR MS system (Bruker, Billerica, MA, USA) equipped with an electrospray ionization source operated in negative-ion mode. Spectra were collected over an *m/z* range of 100–900, and only peaks with a signal-to-noise ratio (S/N) ≥6 were retained for downstream processing [32]. External and internal calibration were applied to achieve sub-ppm mass accuracy. Molecular formulas were assigned using Compass DataAnalysis (Bruker) combined with in-house batch-processing scripts, using a mass error tolerance of ≤0.5 ppm and elemental constraints of C_0–∞_, H_0–∞,_ O_0–∞_, N_0–2_, and S_0–2_, consistent with prior DOM studies [33, 34]. For each assigned formula, we calculated molecular descriptors including the modified aromaticity index (AI_mod_), double-bond equivalents (DBE), nominal oxidation state of carbon (NOSC), H/C and O/C ratios, and Kendrick mass defect (KMD). CRAMs were identified using commonly applied constraints in H/C–O/C space combined with aromaticity criteria.

To further characterize DOM composition, we applied two complementary classification schemes. First, molecules were grouped into Van Krevelen classes (lipid-, protein-, amino sugar-, carbohydrate-, unsaturated hydrocarbon-, lignin-like, condensed aromatic-, and tannin-like) based on H/C and O/C ratio thresholds (ranges adapted from prior studies) [35]. Second, compounds were categorized by aromaticity and heteroatom content using AI_mod_, H/C, O/C, and nitrogen presence, yielding classes such as condensed aromatic structures, aromatic structures, highly unsaturated structures, aliphatics, peptides, and sugar-like molecules [36].

### Metabolomic sample preparation and LC–MS analysis

Metabolomic profiling was performed for all samples to characterize small-molecule organic compounds. Samples stored at −80°C were thawed on ice, extracted with methanol–water containing internal standards, and clarified by centrifugation. The resulting supernatants were filtered and analyzed by ultra-high-performance liquid chromatography coupled with high-resolution mass spectrometry. Each extract was analyzed in both positive- and negative-ionization modes using reverse-phase chromatography. Full-scan MS data were acquired over a broad *m/z* range, with data-dependent tandem mass spectrometry fragmentation used to support metabolite annotation. Detailed extraction procedures, chromatographic gradients, and instrument parameters are provided in Supplementary Methods 2.

### Incubation experiment

To examine DOM transformation under standardized laboratory conditions, incubation systems were established for each salinity regime and sample matrix. For each combination of salinity regime and sample matrix, five field replicates were composited to generate a representative source sample, which was then divided into three parallel incubation microcosms. Incubations were conducted in 10 l transparent borosilicate glass carboys that had been acid cleaned, combusted (450°C for 4 h), and sealed with polytetrafluoroethylene-lined caps to minimize contamination from leachable organic compounds. Microbial inocula were prepared by passing fresh samples through a 53 μm mesh to remove large particles, followed by gentle mixing to obtain microbial suspensions. DOM-containing water was prepared by filtering the corresponding water samples through 0.22 μm membranes to remove most microbial cells but retain DOM. For each treatment, a 10 l incubation system was assembled using 8 l of 0.22 μm-filtered DOM-rich water and 2 l of microbial suspension [37]. For sediment-derived treatments, DOM and microbial fractions were first extracted from the sediment matrix and then recombined at the same volume ratio [25, 38].

At the start of the incubation, salinity in the low-salinity treatment was adjusted to 30 ppt and that in the high-salinity treatment to 90 ppt using sterile concentrated salt solutions prepared from the corresponding lake brines. These targets were set slightly above the mean field salinities (25 and 80 ppt) to maintain stable and clearly separated salinity levels throughout the 100-day incubation despite minor dilution caused by inoculation and repeated subsampling, and remained within the environmentally realistic range of the study system. To minimize unequal nutrient limitation among microcosms and standardize comparisons among treatments, NaNO_3_ and KH_2_PO_4_ were added to establish a nutrient-replete condition based on the day-0 DOC concentration of each incubation microcosm and a target molar C:N:P ratio of 106:16:1; treatment-specific final additions are provided in Table S4. Thus, the incubation was designed to compare DOC/RDOC dynamics among source samples from contrasting salinity regimes under nutrient-replete conditions, rather than to reproduce ambient *in situ* nutrient availability. Incubations were maintained under a 12 h light:12 h dark photoperiod at a controlled light intensity of 50 μmol photons m^−2^ s^−1^. Because all treatments were incubated in the same transparent borosilicate vessels under the same light regime, any photochemical contribution to DOM alteration was standardized across treatments rather than confounded with salinity. Additional details are provided in Supplementary Methods 3.

### Statistical analysis

All statistical analyses were conducted in R (v4.3.3). Bacterial and DOM α-diversity metrics were calculated with the “vegan” package [39]. Differences in community composition were assessed using principal coordinate analysis (PCoA) and non-metric multidimensional scaling (NMDS) based on Bray–Curtis dissimilarities, with ordinations visualized using “ggplot2.” Group separation was tested by permutational multivariate analysis of variance (PERMANOVA) using the *adonis* function in “vegan.” DOM molecular diversity (H′-Mol), elemental-group diversity (H′-Ele), and compound-class diversity (H′-Com) were calculated as Shannon Index values with the *diversity* function in “vegan,” using intensity-weighted abundances of individual FT-ICR MS formulae, elemental groupings, and Van Krevelen–based compound classes. Together, these indices capture DOM richness and evenness across molecular, elemental, and structural dimensions.

KMD analysis was applied to identify homologous series and salinity-responsive molecular families. KMD-CH_2_ and KMD-CO_2_ were calculated from FT-ICR MS *m/z* values in R, and their relationships with salinity were evaluated using Spearman correlations implemented in “psych” package. Molecular formulae showing significant associations (*P* < .05) were visualized as Kendrick plots generated with “ggplot2” package.

To quantify the joint effects of environmental variables and DOM characteristics on bacterial community structure, redundancy analysis (RDA) was performed using “vegan,” with significant predictors identified by *envfit* (999 permutations). Variation partitioning analysis was conducted with *varpart* to apportion explained variance between environmental and DOM predictors after excluding highly collinear variables (|*r*| > 0.9) and those with high multicollinearity, as indicated by the variance inflation factor (VIF > 10); significance was assessed using *anova.cca*. The independent contribution of each predictor was further evaluated by hierarchical partitioning using “rdacca.hp,” with adjusted *R*^2^ from the RDA used as the variance metric [40]. A targeted variation partitioning analysis was additionally conducted on Hellinger-transformed OTU data using salinity (SAL), co-varying chemistry (DOC + TP), and habitat type as three explanatory sets, and the significance of the unique fractions was tested by partial RDA with 999 permutations.

Bacterial community assembly processes were inferred using iCAMP (integrated Community Assembly Mechanisms by Phylogenetic bin-based null-model analysis) implemented in the “iCAMP” package [41]. OTUs were phylogenetically binned, within-bin phylogenetic signal was assessed using *ps.bin*, and null-model analyses were performed with *icamp.big* (1000 randomizations) to derive the β Net Relatedness Index (βNRI) and the Raup–Crick metric based on Bray–Curtis dissimilarity (RC_bray), which were used to quantify the relative contributions of selection, dispersal, and drift. Process contributions were summarized using *icamp.bins*. For DOM, a transformation-weighted characteristic dendrogram (TWCD) was first constructed from FT-ICR MS data based on molecular-property distances and biochemical-transformation networks. TWCD was selected because it incorporates both molecular characteristics and potential transformation connectivity, whereas molecular characteristic dendrograms and transformation-based dendrograms emphasize only one of these dimensions. This integrative framework was considered more suitable for DOM assembly inference in the present study, where both molecular traits and potential transformation relationships were of interest. The TWCD, together with a DOM presence–absence matrix, was then analyzed using the same iCAMP workflow to obtain βNRI, RC_bray, and the relative importance of deterministic versus stochastic assembly processes [42]. Alternative molecular-property distance metrics were not systematically tested; therefore, the inferred process proportions should be interpreted within the chosen TWCD framework. Additional details are provided in Supplementary Methods 4.

Bacteria–DOM association networks were constructed based on Spearman correlations between bacterial OTUs and DOM molecules only [43]. Correlations were computed with the “Hmisc” package, whereas within-matrix correlations (i.e. OTU–OTU or DOM–DOM correlations) were deliberately excluded so that the resulting network would specifically represent cross-domain OTU–DOM associations rather than a full co-occurrence network. This design was chosen to focus on bacteria–DOM coupling and to avoid network topology being dominated by within-bacteria or within-DOM relationships. Accordingly, the resulting module structure should be interpreted as clusters of cross-domain OTU–DOM co-variation, rather than as complete ecological interaction modules or representations of within-domain organization. Under the primary network definition, only strong and significant associations (|*r*| > 0.9, FDR-adjusted *P* < .05) were retained as edges to reduce false positives. To evaluate robustness to threshold choice, the network was additionally reconstructed using a relaxed cutoff (|*r*| > 0.8, FDR-adjusted *P* < .05) as a sensitivity analysis. Networks were assembled and analyzed using the “igraph” package in R. Node roles were classified according to within-module connectivity (Zi) and among-module connectivity (Pi), with peripherals defined as Zi <2.5 and Pi <0.62, connectors as Zi <2.5 and Pi ≥0.62, module hubs as Zi ≥2.5 and Pi <0.62, and network hubs as Zi ≥2.5 and Pi ≥0.62 [44]. Network visualization was performed in Gephi (v0.10). Because the network was inferred from correlation structure, it was interpreted as an exploratory association network rather than as direct evidence of ecological interaction.

Metabolomic profiles were initially explored by principal component analysis (PCA) using *prcomp* in R after unit-variance scaling to visualize overall sample separation [45]. Differential metabolites were defined using the combined criteria of variable importance in projection (VIP) >1, absolute log_2_ fold change (|log_2_FC|) ≥2, and Benjamini–Hochberg (BH) FDR-adjusted *P* <.05. For pairwise comparisons, *P* values were calculated using two-sided Student’s *t-*tests and adjusted using the BH procedure, whereas analysis of variance was applied for multigroup comparisons. VIP scores and orthogonal partial least squares–discriminant analysis (OPLS-DA) modeling, including score plots and 200-permutation tests, were conducted using “MetaboAnalystR” after log_2_ transformation and mean centering. Metabolites were annotated against the KEGG Compound database, and pathway enrichment analysis was performed by mapping significant metabolites to KEGG Pathway entries.

Random forest analysis was performed in R using the “randomForest” package for two purposes: (i) to relate metabolite predictors to DOM molecular variation, represented by the first principal coordinate (PCoA1), and (ii) to evaluate the relative contributions of dominant bacterial families to DOC degradation–related variables in the incubation experiment. In both analyses, predictors were standardized before modeling, samples were randomly divided into training and test sets at a 70:30 ratio using a fixed random seed set.seed (123), and models were fitted with ntree = 500 and the default mtry setting [46]. Predictor importance was evaluated using permutation-based testing, and additional model settings and preprocessing steps for each analysis are provided in Supplementary Methods 5. Because predictor collinearity was not explicitly screened prior to model fitting, variable-importance values were interpreted as relative importance rankings within the predictor set rather than as strictly independent effects.

DOC decay during incubation was fitted using a two-pool exponential decay model [37]:


$$DOC(t)= BDOC\;{\mathrm{e}}^{- kt}+ RDOC\kern1em$$

where *DOC*(*t*) is the DOC concentration at incubation time *t* (days), *BDOC* represents the biodegradable DOC pool, *RDOC* represents the recalcitrant DOC, and *k* is the apparent first-order decay constant of the labile pool. Parameters were estimated for each treatment replicate in R by nonlinear least squares using the *nlsLM* function in the “minpack.lm” package with non-negativity constraints. A multistart procedure was applied by testing 54 combinations of initial values per replicate, and the converged solution with the minimum residual sum of squares was retained. All starting-value combinations converged successfully, and final best-fit parameter estimates did not occur at the imposed lower bounds, indicating that the constraints improved numerical stability without determining the fitted values. Replicate-level parameter estimates were summarized as treatment means with 95% confidence intervals, and model fit was evaluated using the coefficient of determination (*R*^2^) and root mean square error (RMSE). BDOC and RDOC were expressed as both absolute pool sizes and percentages of total modeled DOC. The fitted parameters were also used to generate smoothed DOC decay curves and reconstruct the temporal trajectories of the BDOC and RDOC fractions over the 100-day incubation. In this study, RDOC is operationally defined as the residual DOC fraction remaining after 100 days of incubation, as estimated from the two-pool model. Therefore, it reflects relative DOC persistence at the experimental timescale under the imposed incubation conditions, rather than an absolute measure of intrinsic recalcitrance. Accordingly, this operationally defined RDOC should be interpreted as an incubation-derived proxy for residual carbon preservation at the 100-day timescale, and not as direct evidence of multiyear persistence *in situ*.

Piecewise structural equation modeling (piecewise SEM) was used to evaluate an *a priori* hypothesized pathway linking salinity, bacterial community variation, microbial metabolomic variation, and DOC-related outcomes [47]. The first axis of the Bray–Curtis-based PCoA (PCoA1) was used to represent bacterial community composition, and the first principal component (PC1) from metabolomic PCA was used to summarize microbial metabolic variation. All continuous variables were standardized prior to analysis. Component models were fitted as linear mixed-effects models using *lmer* in the “lme4” package, with sample type included as a random intercept, and were integrated using *psem*. Path directions were specified *a priori* based on the experimental design, with salinity treated as an exogenous variable, microbial variables as intermediate responses, and RDOC and ΔDOC as downstream outcomes. Overall model fit was evaluated using Fisher’s *C* statistic, degrees of freedom, and the associated *P* value, and standardized path coefficients and *R*^2^ values were obtained using *coefs* and *rsquared*, respectively [48].

## Results and discussion

### Salinity is associated with bacterial and DOM diversity and compositional differences

**Figure 1:**
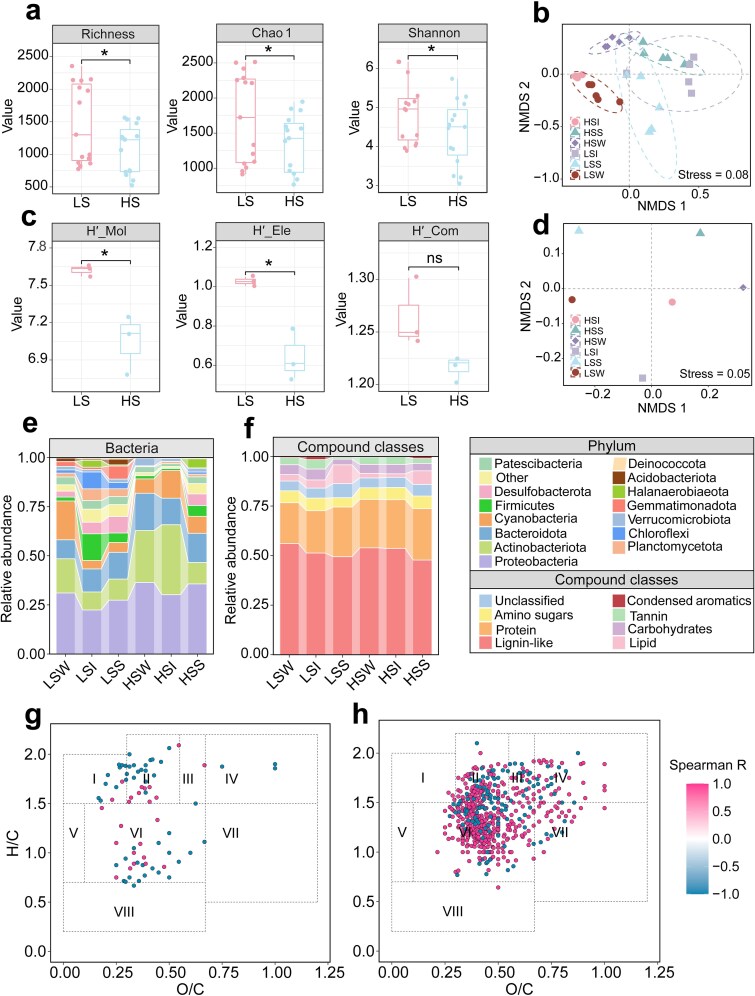
Bacterial and DOM diversity, community structure, and molecular characteristics across low- (LS) and high-salinity (HS) environments. a, b α-Diversity of bacterial communities (a) and DOM diversity (b), quantified as molecular chemodiversity (H′-Mol), elemental combination diversity (H′-Ele), and compound-class diversity (H′-Com), in LS and HS groups. Statistical differences were assessed using two-sided *t-*tests. c, d NMDS ordinations of bacterial communities (c) and DOM (d) across six sample groups: LSW (low-salinity water), LSI (low-salinity water–sediment interface), LSS (low-salinity sediment), HSW (high-salinity water), HSI (high-salinity water–sediment interface), and HSS (high-salinity sediment). e, f Relative abundances of dominant bacterial phyla (e) and DOM compound classes (f). g, h Spearman correlations between DOM molecular formulae and bacterial Shannon index values under low-salinity (g) and high-salinity (h) conditions. *P < .05, **P < .01, ***P < .001.

Salinity was associated with pervasive shifts in both bacterial and DOM diversity, consistent with an environmental filtering effect (Fig. 1). Although the low-salinity (LS) group was already moderately saline (~25 ppt), it still supported significantly higher bacterial observed OTU richness and Shannon Index values than the high-salinity (HS) group (*P* < .05), suggesting that further increases in salinity are associated with lower microbial α-diversity (Fig. 1a). This pattern is consistent with global observations from inland saline waters, where elevated osmotic stress is often associated with substantial metabolic costs and reduced microbial diversity relative to freshwater systems [49, 50]. Beyond diversity metrics, pronounced compositional turnover was evident. NMDS analyses showed clear clustering by both salinity regime and habitat type (stress <0.1; Fig. 1c, d), and Bray–Curtis dissimilarity increased significantly with salinity difference for both bacterial communities (*R*^2^ = 0.24, *P* < .001) and DOM assemblages (*R*^2^ = 0.42, *P* < .01). These relationships indicate a strong salinity-related distance-decay pattern, suggesting that salinity may act as an important environmental barrier associated with rapid community turnover even within the saline range examined here [51, 52] (Supplementary Fig. S2). At the taxonomic level, this turnover manifested as clear community replacement: increasing salinity shifted assemblages from freshwater-associated toward salt-tolerant phyla (Fig. 1e; Supplementary Fig. S3). Actinobacteriota, Bacteroidota, and Proteobacteria, which are typically favored under low ionic strength, declined significantly (*P* < .05), whereas Acidobacteriota and Desulfobacterota increased (*P* < .05). The enrichment of Desulfobacterota is particularly consistent with adaptation to the sulfate-rich conditions characteristic of terminal saline lakes [50, 53, 54]. These phylum-level shifts mirror patterns reported from arid saline soils and lakes and are consistent with the view that salinity functions as an environmental filter associated with reduced niche space for non-halophilic taxa and greater representation of specialized halotolerant lineages [55–57].

**Figure 2:**
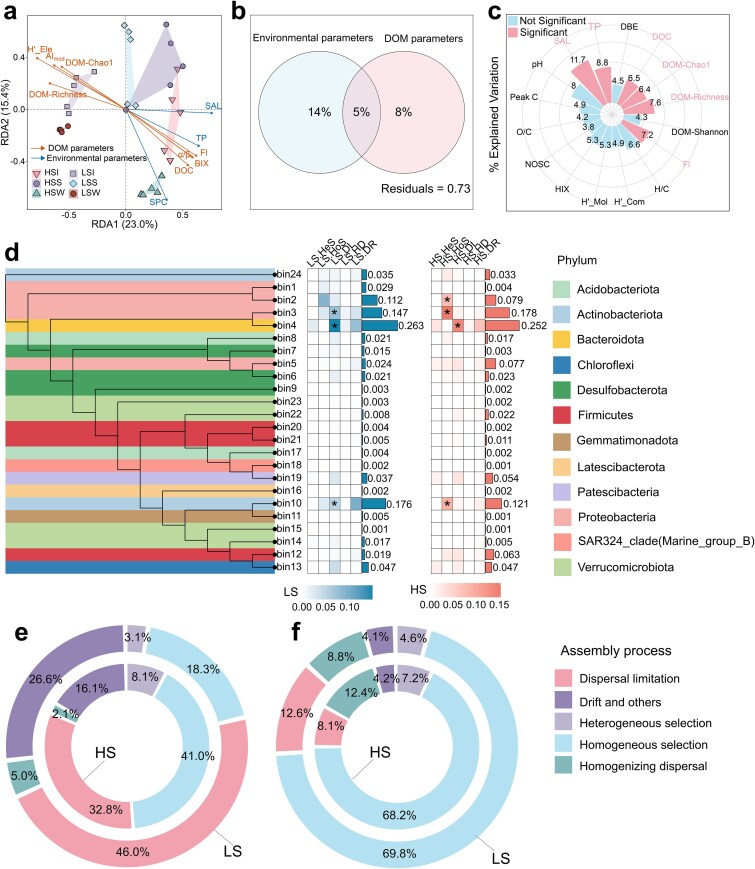
Environmental controls and assembly processes of bacterial communities and DOM molecules. a Redundancy analysis (RDA) showing relationships between bacterial community composition and environmental and DOM variables; only factors with *P* <.05 are shown. b Variation partitioning analysis (VPA) quantifying the fractions of community variation explained uniquely by environmental variables, uniquely by DOM variables, and jointly by both, based on 17 predictors retained after variance inflation factor filtering (VIF < 10). c Hierarchical partitioning illustrating the independent and shared contributions of each environmental and DOM variable to bacterial community variation. d iCAMP-based phylogenetic binning analysis of bacterial community assembly. The phylogenetic tree indicates phylum-level affiliations, and the adjacent heatmap shows, for LS and HS, the relative contributions of heterogeneous selection (HeS), homogeneous selection (HoS), dispersal limitation (DL), homogenizing dispersal (HD), and drift and others (DR) for each bin; asterisks indicate significant processes within a given bin. e, f Donut charts summarizing the proportional contributions of the five ecological processes to the assembly of bacterial communities (e) and DOM molecular assemblages (f) under LS and HS conditions. *P < .05, **P < .01, ***P < .001.

In parallel with bacterial responses, DOM composition showed a comparable salinity-associated filtering pattern. DOM chemodiversity declined with increasing salinity, and both molecular chemodiversity (H′-Mol) and elemental diversity (H′-Ele) were significantly higher in the LS group than in the HS group (*P* < .01 and *P* < .05, respectively; Fig. 1b). This contraction of molecular diversity may reflect two nonexclusive processes. Under low-salinity conditions, diverse allochthonous and autochthonous compounds can coexist [58], whereas elevated salinity may enhance physicochemical removal processes (e.g. flocculation of humic substances) and microbial reworking, potentially leaving a narrower pool dominated by recalcitrant compounds or continuously produced halophilic metabolites [13]. This reduction in chemical diversity may be linked to reduced microbial niche differentiation, as variance partitioning analysis showed that DOM diversity indices explained a significant proportion of bacterial community variation (Fig. 2b). Thus, chemically heterogeneous environments tended to support more diverse microbial assemblages in this study, consistent with close coupling between DOM and microbial diversity [35, 59, 60]. At the molecular level, the LS group was enriched in CHO-only formulas and exhibited higher proportions of lignin-like, lipid, condensed aromatic, and CRAM-type molecules, along with slightly higher AI_mod_ and DBE values and lower NOSC values, features characteristic of terrestrially influenced, slowly processed DOM [61] (Fig. 1f; Tables S5 and S6). In contrast, DOM in the HS group shifted toward sulfur- and nitrogen-containing formulas (CHSO and CHSNO) and protein-like compounds, accompanied by higher H/C ratios and NOSC values and lower AI_mod_ and DBE. These features are consistent with more saturated, oxidized, and microbially derived DOM [22, 62]. This pattern is consistent with known physicochemical effects associated with higher salinity, including reduced solubility and flocculation of aromatic moieties, potentially combined with enhanced microbial reworking in hypersaline waters, which may contribute to the depletion of diverse aromatic compounds and the relative persistence of recalcitrant or halophile-derived molecules [13, 58]. Fluorescence properties further supported this transition. Three fluorescence components were identified in both salinity groups: two humic-like components (C1 and C2) and one protein-like component (C3) (Supplementary Fig. S4; Table S7). The LS group exhibited a higher proportion of protein-like fluorescence (C3 = 40.4%), whereas the HS group was dominated by humic-like components (C1 = 41.7%, C2 = 42.7%). Correspondingly, *F*_max_ values for C1 and C2 were significantly higher in the HS group (*P* < .05), whereas C3 fluorescence was significantly higher in the LS group (*P* < .05). Fluorescence indices revealed additional salinity-dependent patterns: FI, BIX, and β/α were significantly elevated in the HS group (*P* < .001), indicating stronger autochthonous and microbially derived DOM signatures [28–30], whereas HIX was significantly higher in the LS group (*P* < .05), reflecting a more aromatic and humified DOM character [63] (Supplementary Fig. S5). This apparent paradox, namely, greater humic-like fluorescence in the HS group despite lower structural condensation, suggests a shift in dominant DOM generation pathways. Specifically, high salinity may favor the accumulation of microbially derived humic-like fluorescent DOM that is relatively “young” and weakly condensed, whereas the higher HIX values in the LS group reflect the persistence of structurally condensed, aromatic, allochthonous humic materials under lower ionic stress [64, 65]. Similar patterns have been reported across river-estuary transitions and other aquatic systems spanning contrasting salinity conditions, where FI and BIX typically increase with *in situ* microbial DOM production, whereas HIX remains higher when DOM is dominated by older, soil- and plant-derived humics [29, 66]. In marine and hypersaline systems, humic-like fluorescence often represents relatively small, weakly condensed “marine humics” formed *in situ* rather than highly aromatic terrestrial humics [67, 68]. Together, these observations are consistent with the interpretation that increasing salinity was associated with a shift in fluorescent dissolved organic matter sources and structure from allochthonous, aromatic humics toward more microbially produced, less condensed humic-like substances [69]. KMD patterns were consistent with this interpretation. The LS group exhibited both positive and negative correlations with salinity within a relatively narrow mass range, whereas the HS group was dominated by positively correlated molecular clusters spanning a broader mass range (Supplementary Fig. S6). This pattern is consistent with selective retention of salt-tolerant, often more oxidized and hydrophobic molecules under hypersaline conditions, and with a DOM pool experiencing stronger thermodynamic and physicochemical filtering [70]. Similar broadening and oxidation of salinity-correlated molecular fractions have been reported in other hypersaline systems during lake concentration and brine exchange [13, 61]. Finally, correlations between individual DOM molecules and bacterial α-diversity (Shannon Index values) differed markedly between salinity regimes (Fig. 1g, h). Few significant associations were detected in the LS group, whereas the HS group exhibited a higher density and broader distribution of correlated molecules, suggesting tighter statistical coupling between DOM composition and microbial diversity under hypersaline conditions [71]. Collectively, fluorescence and molecular evidence suggest that low-salinity environments were associated with a more diverse, aromatic, and strongly humified DOM pool dominated by allochthonous inputs, whereas higher salinity was associated with a less diverse but more microbially reworked, oxidized humic-like assemblage associated with a subset of salt-adapted bacterial taxa.

### Environmental control and assembly mechanisms of bacteria and DOM across contrasting salinity regimes

Having established pronounced salinity-associated turnover in both community composition and DOM chemistry (Fig. 1), we next examined how community structure was associated with physicochemical variables and whether these patterns were consistent with shifts in assembly processes. RDA and hierarchical partitioning identified salinity as the strongest environmental correlate, explaining the largest independent fraction of variation (11.7%, *P* < .05). HS communities were associated with higher salinity, TP, and DOC, as well as stronger autochthonous signatures (FI and BIX) (Fig. 2a, c). A targeted variation partitioning analysis further showed that salinity accounted for the largest unique fraction of bacterial community variation (10.1%, *P* = .001), whereas co-varying chemistry (DOC + TP; 5.4%, *P* = .003) and habitat type (1.6%, *P* = .039) also explained significant unique fractions (Supplementary Fig. S7), indicating that bacterial community variation reflected the joint influence of salinity, chemistry, and habitat context. This independent association of salinity with bacterial community variation is consistent with global meta-analyses of inland waters, in which ionic strength is frequently identified as an important broad-scale correlate of microbial biogeography across continental scales [72–75]. An additional explanatory factor also emerged. LS communities were closely associated with DOM diversity indices (H′-Ele and richness), and DOM variables alone explained 8% of community structural variation (Fig. 2b). This pattern is consistent with the Chemodiversity Hypothesis, which posits that a more diverse chemical environment expands niche space and thereby sustains higher microbial diversity [59, 76, 77]. Thus, although salinity appears to act as a broad environmental filter, finer-scale variation in bacterial community structure may also be associated with the diversity of available organic substrates [21, 78]. Such strong environmental filtering would be expected to coincide with a shift from more stochastic to more deterministic assembly. Consistent with this expectation, iCAMP analyses based on phylogenetic binning showed that bacterial assembly transitioned from stochastic dominance in the LS group (DL: 46.0%; HD: 18.3%) to deterministic dominance in the HS group (HeS: 41.0%; HoS: 32.8%) (Fig. 2e; Supplementary Fig. S8). This shift is consistent with the Stress Gradient Hypothesis in microbial ecology, which predicts that stochastic processes (e.g. drift and dispersal) prevail under relatively benign conditions, whereas harsh environments impose strong selection for specific stress-tolerant traits [41, 79, 80]. At finer resolution, phylogenetic binning revealed bin-specific responses to salinity stress (Fig. 2d). Under LS conditions, bin3 (Proteobacteria) and bin4 (Bacteroidota) were primarily governed by dispersal limitation, whereas bin10 (Actinobacteriota) was dominated by drift (*P* < .05), suggesting that community assembly in lower-ionic-strength waters may be more historically contingent and more strongly influenced by stochasticity [72, 81]. Under HS conditions, these patterns reversed; bin2 and bin3 (Proteobacteria), together with bin10 (Actinobacteriota), shifted significantly toward homogeneous selection (*P* < .05). The trajectory of bin10, from stochastic drift in LS to stronger deterministic selection in HS, is particularly notable and suggests that intense salinity stress may be associated with more convergent selection, leading to increasingly similar communities across sites (i.e. stronger homogeneous selection) even when initial compositions differ [56].

In contrast to the shifts observed for bacterial communities, DOM molecules exhibited a different pattern, suggesting partial decoupling in assembly dynamics between biotic and abiotic components (Supplementary Fig. S9). DOM assembly was consistently dominated by homogeneous selection under both salinity regimes (LS: 69.8%; HS: 68.2%; Fig. 2f). This suggests that DOM chemistry may be shaped by strong and relatively invariant influences, such as thermodynamic filtering, source inputs, and recurring microbial processing, which together could contribute to similar molecular signatures within a given salinity regime [82]. Together, these contrasting assembly patterns suggest partial decoupling: bacterial communities showed greater variation in assembly pathways and a salinity-associated shift toward stronger deterministic assembly, whereas DOM composition exhibited a more consistently homogeneous assembly pattern across the two contrasting salinity regimes [10, 83, 84]. Although these assembly patterns help define the ecological context of salinity-associated restructuring, they do not by themselves resolve how carbon is processed; the more direct process-level evidence for DOC degradation and RDOC preservation is provided by the subsequent metabolomic and incubation analyses.

### Salinity-associated reconfiguration of bacteria–DOM association networks

**Figure 3:**
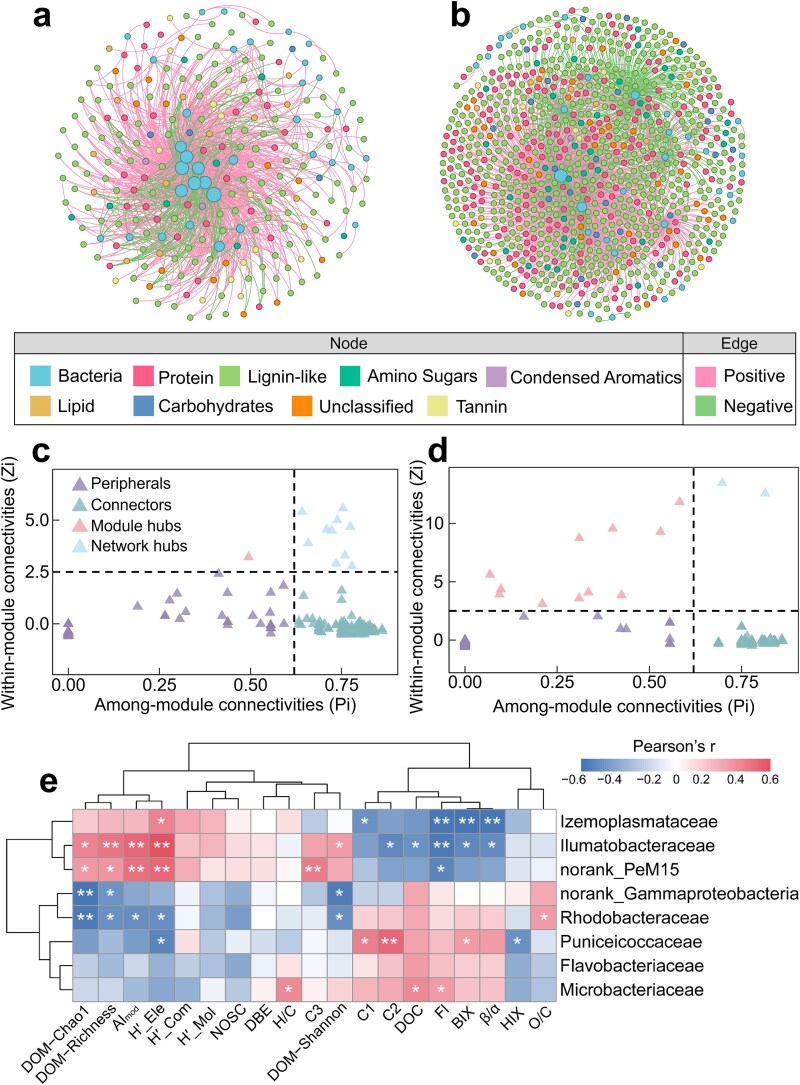
Bacteria–DOM association networks and topological hub taxa under contrasting salinity conditions. a, b Association networks linking bacterial OTUs and DOM molecular classes in the LS (a) and HS (b) groups. Node categories distinguish bacterial taxa and different DOM molecular classes, node size scales with connectivity, and edge attributes indicate positive or negative correlations. c, d Topological role analysis identifying topological hub taxa in the LS (c) and HS (d) networks based on within-module connectivity (Zi) and among-module connectivity (Pi). Nodes were classified as peripherals, connectors, module hubs, or network hubs according to their Zi–Pi positions. Network hubs and module hubs were considered topologically important nodes because of their disproportionate structural positions in the association network. Taxonomic annotation indicated that LS hubs were primarily affiliated with Proteobacteria, Actinobacteriota, Bacteroidota, and Firmicutes, including Rhodobacteraceae, Microbacteriaceae, Flavobacteriaceae, Cryomorphaceae, Ilumatobacteraceae, norank_Izemoplasmatales, and norank_PeM15. In contrast, HS hubs were mainly assigned to Verrucomicrobiota and Proteobacteria, represented by Puniceicoccaceae and norank_Gammaproteobacteria (Table S10). e Pearson correlations between family-level network hubs and DOM indices; the correlation matrix indicates Pearson’s *r*, and asterisks denote significant correlations. *P < .05, **P < .01, ***P < .001.

The shifting balance of diversity and assembly processes was reflected in the architecture of the bacteria–DOM association networks. In the LS group, the network was relatively small (263 nodes) yet dense (1177 edges; density = 0.03), with predominantly positive associations between bacteria and DOM molecules (Fig. 3a; Table S8). The average path length and diameter were short, and bacterial nodes consistently exhibited higher centrality metrics than DOM nodes in both salinity groups (*P* < .001; Table S9). This compact and highly connected architecture is consistent with a more cohesive association structure under lower salinity, in which a chemically diverse resource landscape may allow multiple metabolic guilds to coexist through partial resource partitioning and sequential substrate transformation [59, 85, 86]. Such a structure is often associated with functional redundancy and buffering capacity in comparatively less stressful environments [87, 88]. By contrast, the HS network contained many more nodes (916) and edges (1847) but exhibited much lower density (0.004), longer path lengths, and a larger diameter (Fig. 3b; Table S8), indicating a more expansive but less cohesive association structure. The proportion of negative bacteria–DOM associations nearly doubled (from 18.2% in the LS group to 34.7% in the HS group), and the network partitioned into more distinct modules, indicating stronger compartmentalization under hypersaline conditions. Shifts in centrality patterns were also evident, with betweenness and closeness centralities higher in the HS group, whereas degree and eigenvector centralities were higher in the LS group (*P* < .001; Supplementary Fig. S10). Together, these patterns suggest that higher salinity was associated with reduced overall connectivity and greater dependence on a smaller set of bridging nodes. This interpretation is also consistent with the robustness analysis, which showed that the HS network was more vulnerable to node removal (Supplementary Fig. S11), implying lower structural resilience. Although such topological contrasts are consistent with stronger specialization, compartmentalization, and reduced cohesion under high salinity [89–91], the inferred links should be interpreted as correlation-based associations rather than direct ecological interactions because both compositional structure and shared environmental drivers may contribute to the observed network architecture.

Correlation analyses of bacteria-associated DOM classes further supported these contrasts: lignin-like and protein-like compounds were the dominant DOM classes involved in bacterial associations (Supplementary Fig. S12). Keystone analysis likewise highlighted the central role of bacteria in maintaining cross-domain connectivity because all network hubs and module hubs identified in both salinity regimes were bacterial OTUs rather than specific DOM formulas (Fig. 3c, d; Table S10), consistent with previous work showing that highly connected bacterial taxa can disproportionately organize complex association structures [59, 92, 93]. In the LS group, 10 network hubs and two module hubs were identified, including Ilumatobacteraceae and norank_PeM15 (Actinobacteria). These topological hub taxa showed positive associations with AI_mod_, H′-Ele, and DOM diversity indices, but negative associations with autochthonous fluorescence indices (FI, BIX, and β/α) (Fig. 3e), consistent with a central position in more aromatic-rich and chemically diverse DOM settings. This interpretation is also compatible with the capacity of many Actinobacteria to transform high-molecular-weight and aromatic compounds in aquatic systems [94]. By contrast, the HS network exhibited a much narrower hub composition under the primary threshold, with only two network hubs identified: Puniceicoccaceae (Verrucomicrobiota) and norank_Gammaproteobacteria. The Puniceicoccaceae hub was positively associated with humic-like components and BIX but negatively associated with H′-Ele, whereas the norank_Gammaproteobacteria hub showed negative associations with DOM diversity indices but positive associations with protein-like components, suggesting distinct patterns of linkage with DOM composition under hypersaline conditions. These patterns are consistent with previous studies showing that Verrucomicrobiota can participate in polysaccharide degradation and contribute to the transformation of more recalcitrant byproducts [95], whereas halotolerant Gammaproteobacteria are often metabolically versatile consumers capable of rapidly exploiting diverse organic substrates in hypersaline environments [96, 97]. These main patterns were robust to threshold choice. When the association network was reconstructed using a relaxed cutoff (|r| > 0.8, FDR-adjusted *P* < .05), the major topological contrasts between LS and HS remained qualitatively consistent, and the identities of the core hub nodes were largely preserved (Supplementary Fig. S13; Table S11; Table S12). Together, these results indicate that the principal biological interpretations derived from the bacteria–DOM network comparison were stable, even though the absolute number of retained associations increased under the relaxed threshold. Accordingly, these network results are interpreted here primarily as evidence of salinity-associated reorganization in cross-domain bacteria–DOM co-variation, whereas inference regarding DOC/RDOC fate relies mainly on the incubation-based carbon-processing patterns and their associated metabolomic signatures presented below.

### Salinity-associated metabolomic shifts and their coupling to DOM molecular features

**Figure 4:**
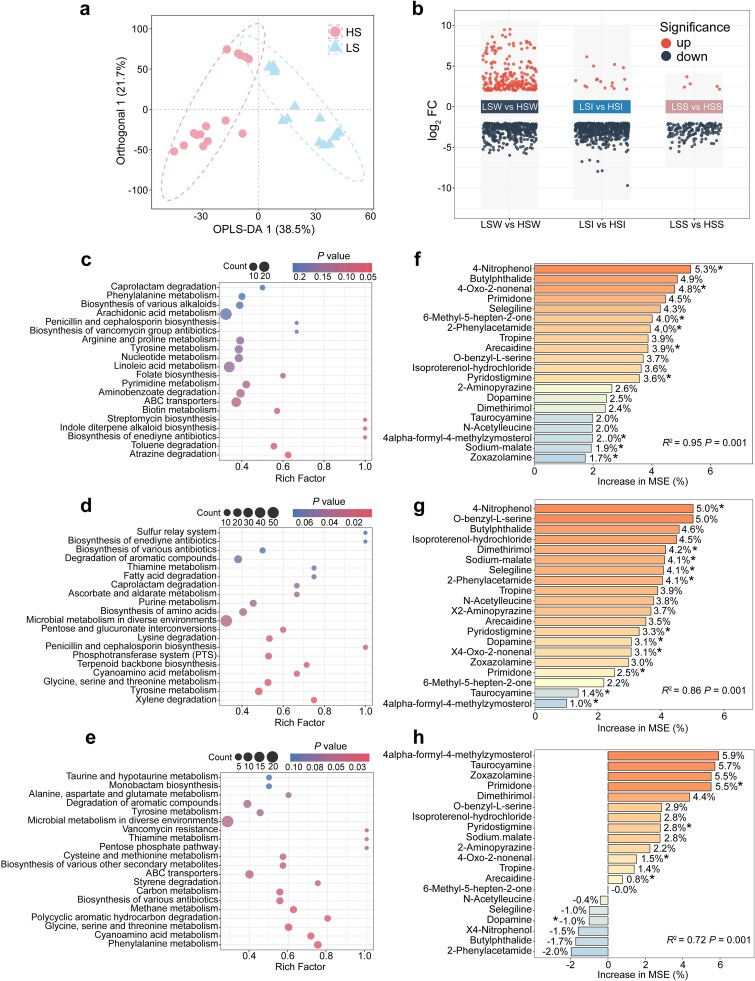
Salinity-associated shifts in metabolite profiles and key differentially abundant metabolites across matrices. a Orthogonal partial least squares discriminant analysis (OPLS-DA) score plot showing separation between low-salinity (LS) and high-salinity (HS) samples based on metabolite profiles. b Volcano plots illustrating pairwise metabolite comparisons between LS and HS across the three matrices: LSW vs HSW (water), LSI vs HSI (water–sediment interface), and LSS vs HSS (sediment). Metabolites exhibiting significant differences between HS and LS are highlighted, with point categories indicating significantly upregulated and downregulated metabolites in HS. Differential metabolites were identified using the criteria *P* <.05, |log_2_FC| ≥2, and VIP >1. c–e KEGG pathway enrichment analysis of metabolites significantly upregulated in HS relative to LS in water (c), water–sediment interface (d), and sediment (e). Enrichment *P* values were calculated using a hypergeometric test, and the top 20 pathways (ranked by increasing *P* value) are shown. The *x*-axis denotes the rich factor (the ratio of enriched metabolites to all annotated metabolites within each pathway), and the *y*-axis lists pathway names ordered by significance. Bubble size represents the number of enriched metabolites, and bubble shading indicates the corrected enrichment *P* value. f–h Random forest analyses of the 20 metabolites consistently elevated in HS across all three matrices, showing their importance in predicting DOM molecular features in water (f), water–sediment interface (g), and sediment (h). Model performance metrics (*R*^2^ and *P* value) are provided in each panel. *P < .05, **P < .01, ***P < .001.

To assess whether these compositional shifts were accompanied by differences in metabolomic profiles, we next examined small-molecule profiles across the two contrasting salinity regimes. OPLS-DA showed separation between LS and HS samples across the water, interface, and sediment matrices, indicating distinct metabolic states under contrasting salinity regimes (Fig. 4a). Consistent with this separation, the LS group exhibited substantially more downregulated than upregulated metabolites relative to the HS group (water: 230 up- and 417 downregulated; interface: 16 and 570; sediment: 5 and 157), indicating that a broad suite of metabolites was maintained at higher relative abundance under hypersaline conditions (Fig. 4b). Rather than indicating uniform metabolic suppression or dormancy, this broad enrichment is consistent with metabolomic reorganization under salt stress. It suggests that hypersaline communities may remain metabolically active, and the taxa that persist under these conditions are associated with metabolite profiles consistent with energetic homeostasis and stress-responsive chemistry [98, 99]. Such patterns are also consistent with greater emphasis on energy acquisition and compatible-solute-related metabolism [100, 101], processes often linked to amino acid turnover and widely recognized as important for stabilizing proteins and maintaining enzymatic function under high ionic strength [102, 103]. KEGG pathway enrichment provided additional context for these metabolomic differences. Across matrices, differentially abundant metabolites were mainly associated with amino acid metabolism, microbial secondary metabolism, and the degradation of aromatic and xenobiotic compounds (Fig. 4c, d). In the water, pathways related to antibiotic biosynthesis and the breakdown of compounds such as caprolactam and toluene were prominent, consistent with potential transformation of complex organics under hypersaline conditions. At the water–sediment interface, enriched pathways included amino acid metabolism, fatty acid degradation, and transport-related processes (e.g. the phosphotransferase system), consistent with intensive substrate uptake and energy generation in this exchange zone. In sediments, pathways related to carbon and methane metabolism and the degradation of polycyclic aromatic hydrocarbons were dominant, and high-salinity sediment communities were accompanied by metabolites linked to the transformation of structurally complex compounds. Together, these enrichment patterns are consistent with a broad catabolic orientation in hypersaline environments, in which diverse substrates, including aromatic and xenobiotic compounds often considered relatively refractory, may be routed into energy-yielding metabolism and stress-tolerance-related pathways [104–106]. This signature of opportunistic scavenging is consistent with prior evidence indicating that halophilic consortia, including genera such as *Halomonas* and *Marinobacter*, can maintain high catabolic versatility under high salinity, with demonstrated capacities to degrade aromatic hydrocarbons and polycyclic aromatic hydrocarbons at elevated NaCl concentrations [97, 107, 108].

To identify which HS-enriched metabolites were most closely linked to DOM molecular features, we focused on 20 compounds that were consistently elevated in HS across all matrices. These metabolites were annotated, and their predictive importance was evaluated using random forest models (Fig. 4f–h; Table S13). Among them, 4-nitrophenol emerged as the strongest predictor of DOM molecular characteristics in both the water and the water–sediment interface (*P* < .05; Fig. 4f, g). As a phenolic intermediate involved in 4-aminobenzoate degradation and xenobiotic metabolism, the prominence of 4-nitrophenol suggests that aromatic-compound transformation may represent one important link between hypersaline metabolomic patterns and the observed DOM features [109, 110]. The remaining key metabolites were dominated by aromatic compounds, nitrogen-containing amines, heterocyclic intermediates, and lipid-peroxidation products (Table S13), spanning biochemical routes related to aromatic-ring cleavage, amino acid turnover, and oxidative degradation; these patterns were also consistent with the pathway-level signals described above.

Taken together, the metabolomic signatures and DOM molecular patterns are consistent with a plausible bioenergetic trade-off in carbon allocation across the two salinity regimes. Under LS conditions, where osmotic stress is comparatively mild, a larger fraction of carbon may be routed toward anabolic processes and extracellular products, consistent with the microbial carbon pump framework that emphasizes the accumulation of aromatic-rich, humic-like, high-AI_mod_ DOM through microbial reworking and retention [15, 111]. Under hypersaline conditions, by contrast, the enrichment of catabolism-related pathways is consistent with greater oxidative processing of organic matter, such that DOM may be more extensively transformed into simpler, more oxidized, and less diverse molecules [20, 112, 113]. This metabolomic shift is consistent with the coexistence of a smaller yet more oxidized DOM pool in the HS group and with the idea that hypersaline conditions favor microbial investment in energetic and osmotic maintenance rather than in the production or preservation of long-lived DOM [13, 61]. These results further suggest that the high-salinity regime may be associated with lower residual DOC preservation in dissolved pools under the studied conditions [114, 115], a possibility that we further evaluated in the subsequent incubation experiment.

### Salinity-associated changes in DOC turnover and their metabolomic signatures

**Figure 5:**
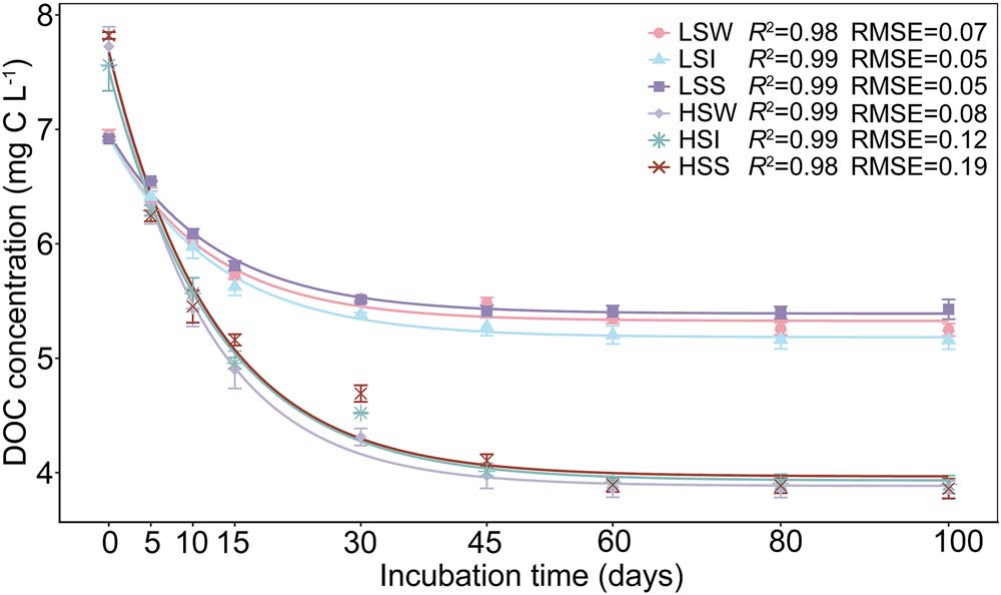
DOC decay dynamics during a 100-day incubation under contrasting salinity conditions. Time-series profiles of DOC concentration during a 100-day incubation for six treatments: LSW (low-salinity water), LSI (low-salinity water–sediment interface), LSS (low-salinity sediment), HSW (high-salinity water), HSI (high-salinity water–sediment interface), and HSS (high-salinity sediment). DOC declined rapidly during the early stage of incubation and then gradually approached a plateau. Symbols represent treatment means, and error bars indicate standard errors. Solid lines show fitted decay trajectories based on a two-pool first-order exponential model, in which DOC is partitioned into BDOC and RDOC. Goodness-of-fit statistics for each treatment, including the coefficient of determination (*R*^2^) and root mean square error (RMSE), are shown in the corresponding panels and provided in Table S15.

To examine whether the salinity-associated patterns observed in the field were also reflected in carbon-processing outcomes under standardized incubation conditions, we incubated samples under low- and high-salinity treatments and tracked DOC loss over 100 days. Across all treatments, DOC declined rapidly at the beginning of the incubation and then gradually approached a plateau (Fig. 5). The two-pool model provided a generally good fit to the DOC decay trajectories across treatments, with *R*^2^ ranging from 0.98 to 0.99 and RMSE ranging from 0.05 to 0.19, although fit quality varied modestly among treatments, with HSS showing the lowest fit quality and HSW the highest (Fig. 5; Table 1). Although the fitted decay constants (*k*) did not differ significantly between salinity treatments (*P* > .05), BDOC, RDOC, and total DOC loss (ΔDOC) diverged markedly (*P* < .05): HS incubations exhibited higher BDOC, lower final RDOC, and greater overall DOC loss (Fig. 5; Table 1). In other words, elevated salinity did not increase how fast DOC was removed once accessible, but increased how much of the DOC pool became accessible within the experimental timeframe. This pattern suggests that, under the standardized incubation conditions used here, high salinity reduced the effective preservation of a fraction of DOM that remained relatively protected under lower-salinity conditions. This result is consistent with the view that apparent recalcitrance can depend on environmental context, including factors that influence microbial accessibility and processing, rather than reflecting chemical inertness alone [116–121]. Higher ionic strength may influence substrate availability, e.g. by altering solubility and aggregation, shifting sorption or colloidal partitioning, and modifying enzyme–substrate encounter rates, potentially shifting some compounds from an effectively “protected” pool into a more bioavailable state [101, 106]. This interpretation is consistent with previous observations that halophilic consortia can sustain substantial degradation capacity under brine-like salinities, allowing efficient turnover once substrates become accessible [20, 122, 123]. An additional point is that this HS–LS contrast cannot be readily attributed solely to differences in nutrient background among the source samples. In the two habitats for which pre-incubation nutrient data were available, the high-salinity groups did not show systematically lower nutrient status; instead, TP and DOC were significantly higher in HS than in LS in both water and interface habitats, whereas NH_4_^+^-N was not lower in HS and TN did not decrease significantly relative to LS (Table S1; Table S2). Nevertheless, the incubation outcomes remained directionally consistent across both habitats, with higher ΔDOC and BDOC proportion but lower RDOC proportion in the high-salinity treatments (Table 1). Together, these results indicate that although nutrient amendment may have influenced the absolute magnitude of DOC turnover, the reduced RDOC preservation under high salinity cannot be readily explained by nutrient relief alone. Rather, the pattern is more consistent with different DOC/RDOC processing outcomes among source samples from contrasting salinity regimes under nutrient-replete conditions, although the present design does not fully disentangle salinity effects, nutrient-amendment effects, and their possible interaction.

**Table 1:** Kinetic parameters of the two-pool DOC degradation model.

Index	Within group						Between group	
	LSW	LSI	LSS	HSW	HSI	HSS	LS	HS
*k* (day^−1^)	0.084 ± 0.002	0.084 ± 0.014	0.080 ± 0.002	0.088 ± 0.010	0.079 ± 0.008	0.080 ± 0.005	0.081 ± 0.002^a^	0.081 ± 0.004^a^
BDOC (mg/l)	1.611 ± 0.078	1.767 ± 0.117	1.576 ± 0.072	3.823 ± 0.209	3.592 ± 0.175	3.707 ± 0.031	1.646 ± 0.076^a^	3.700 ± 0.096^b^
RDOC (mg/l)	5.326 ± 0.045	5.173 ± 0.095	5.389 ± 0.581	3.875 ± 0.069	3.927 ± 0.084	3.962 ± 0.041	5.300 ± 0.086^a^	3.928 ± 0.034^b^
∆DOC (mg/l)	1.704 ± 0.095	1.755 ± 0.154	1.490 ± 0.148	3.871 ± 0.348	3.665 ± 0.314	3.960 ± 0.163	1.650 ± 0.177^a^	3.832 ± 0.312^b^
BDOC proportion (%)	23.2 ± 0.9	25.5 ± 1.6	22.6 ± 1.0	49.6 ± 1.8	47.7 ± 1.4	48.3 ± 0.5	23.8 ± 0.02^a^	48.6 ± 0.002^b^
RDOC proportion (%)	76.8 ± 0.9	74.6 ± 1.6	77.37 ± 1.0	50.4 ± 1.8	52.3 ± 1.4	51.7 ± 0.5	76.2 ± 0.02^a^	51.5 ± 0.002^b^

Values are reported as mean ± SD and include the apparent first-order decay constant (*k*, day^−1^), biodegradable DOC (BDOC), recalcitrant DOC (RDOC), total DOC loss (ΔDOC), and the initial-pool proportions of BDOC and RDOC. Within-group columns report parameter estimates for LSW (low-salinity water), LSI (low-salinity water–sediment interface), LSS (low-salinity sediment), HSW (high-salinity water), HSI (high-salinity water–sediment interface), and HSS (high-salinity sediment), whereas between-group columns summarize pooled LS and HS treatments. Different superscript letters in the LS and HS columns indicate significant differences between salinity groups for a given parameter (*P* < .05, two-sided *t*-test).

**Figure 6:**
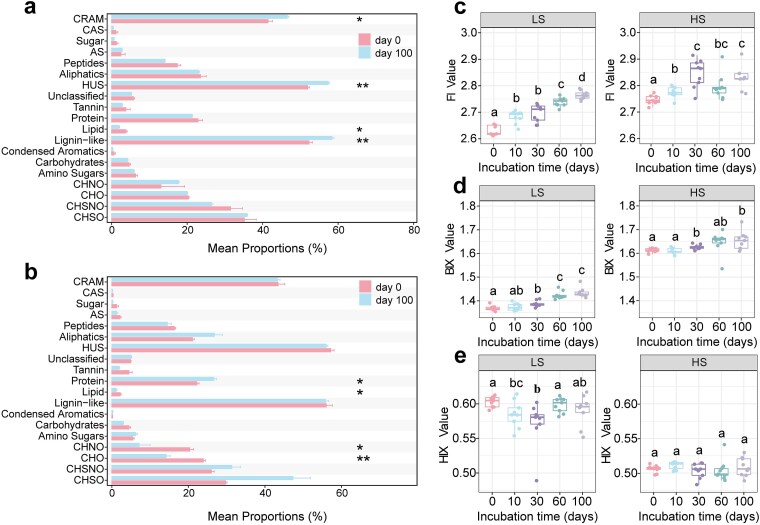
Temporal shifts in DOM molecular composition and fluorescence indices during a 100-day incubation. a, b Changes in the relative abundances of DOM molecular classes between day 0 and day 100 in the LS (a) and HS (b) groups. Bars represent mean proportions (%) for each compound class; differences between day 0 and day 100 were tested using Welch’s *t*-test. c–e Temporal dynamics of three fluorescence indices, FI (c), BIX (d), and HIX (e), across incubation time points (days 0, 10, 30, 60, and 100) in the LS and HS groups. Within each salinity level, differences across time were assessed using the Friedman test, and different letters indicate significant pairwise differences among time points. *P < .05, **P < .01, ***P < .001.

Parallel shifts in DOM composition and intracellular metabolism provided insight into how salinity was associated with altered carbon processing during incubation (Fig. 6; Supplementary Fig. S14). Over 100 days, LS incubations accumulated lignin-like, CRAM-like, and humic-like fractions, whereas lipid-like components declined significantly (*P* < .05; Fig. 6a, b). Fluorescence indices in the LS group showed higher HIX and lower FI and BIX (Fig. 6c–e), together with molecular signatures of greater aromaticity (AI_mod_, DBE) and lower oxidation state (NOSC) compared with HS treatments (Table S14). Intracellular KEGG profiles provide additional context for this pattern: LS communities were enriched in metabolites associated with biosynthesis- and growth-related pathways (e.g. amino acid anabolism and vitamin/cofactor production), consistent with preferential use of labile substrates together with microbial reworking of residual material into more refractory structures [20, 92, 124]. This experimental coupling of anabolism and recalcitrant DOM accumulation was broadly consistent with the signature of field LS samples, which likewise showed higher proportions of CHO and aromatic-like components (Fig. 1; Table S5; Table S6). In HS incubations, CHO-, CHNO-, and lipid-like components declined sharply, and protein-like molecules increased significantly (*P* < .05); furthermore, FI and BIX remained higher, and HIX remained lower in HS than in LS (Fig. 6). AI_mod_ and DBE decreased, and H/C and NOSC increased (Table S14), indicating lower aromaticity, higher saturation, and stronger oxidation of DOM. The HS metabolic profile was consistent with this trajectory: incubations emphasized energy extraction and stress responses, including quinone biosynthesis for respiration, glycolysis and TCA-linked routes via the pentose phosphate pathway and glyoxylate shunt, as well as pathways associated with xenobiotic degradation and reactive oxygen species management (Supplementary Fig. S14). This more oxidative metabolic pattern is consistent with thermodynamic expectations that more oxidized substrates can become energetically favorable under high ionic strength [106, 125]. Moreover, it mirrors the field metabolomics and KEGG patterns observed across the two contrasting salinity regimes (Fig. 4), which was consistent with stronger amino-acid-associated turnover signals and enhanced aromatic/xenobiotic transformation. These findings, together with the greater DOC loss, suggest that under the incubation conditions used here, high salinity was associated with more extensive oxidative processing of carbon and with the persistence of a smaller, more microbially derived, and more oxidized DOM pool [21, 61, 126].

**Figure 7:**
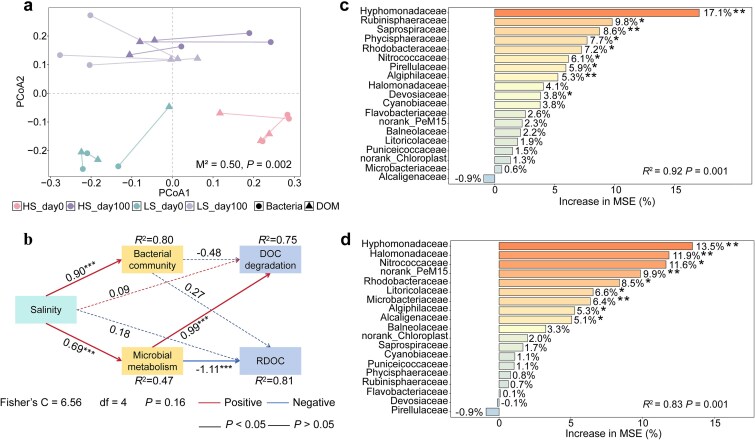
Coupling between bacterial communities, DOM dynamics, and DOC degradation under contrasting salinities. a Procrustes analysis comparing bacterial and DOM ordinations at day 0 and day 100 under low-salinity (LS) and high-salinity (HS) conditions. Points connected by arrows indicate matched bacterial-DOM configurations. The Procrustes statistic *M*^2^ represents the summed squared distance between the two configurations after optimal superimposition; lower *M*^2^ values indicate stronger concordance between bacterial and DOM patterns. The associated *P* value was obtained by permutation testing and evaluates whether the observed concordance exceeds that expected by chance. b Structural equation model (SEM) evaluating hypothesized statistical pathways linking salinity, bacterial community structure, microbial metabolism, DOC degradation, and RDOC accumulation. Arrow thickness is proportional to standardized path coefficients. The sign of each standardized path coefficient indicates whether the corresponding effect is positive or negative. Solid lines denote significant paths (*P* < .05), whereas dashed lines indicate nonsignificant paths. *R*^2^ values beside each response variable indicate the proportion of variance explained, and overall model fit is summarized by Fisher’s *C*, degrees of freedom (*df*), and *P* value. c, d Random forest analyses evaluating the contribution of bacterial families to DOC degradation in LS (c) and HS (d) incubations. Models were built using the 20 most abundant families; bar length indicates the percent increase in mean squared error (MSE) following permutation of each family’s abundance, with higher values reflecting greater predictive importance. Model performance metrics (*R*^2^ and *P* value) are reported in each panel. *P < .05, **P < .01, ***P < .001.

To evaluate whether bacterial and DOM responses were coordinated and to explore the statistical pathways through which salinity was associated with DOC loss, we next evaluated their joint trajectories. Procrustes analysis indicated strong concordance between bacterial and DOM dynamics (*M*^2^ = 0.497, *P* = .003; Fig. 7a). Structural equation modeling was then used to evaluate an *a priori* hypothesized pathway linking salinity, microbial variables, DOC degradation, and RDOC dynamics (Fig. 7b). The hypothesized piecewise SEM showed an acceptable overall fit (Fisher’s *C* = 6.56, *df* = 4, *P* = .161). Salinity was strongly associated with both bacterial community composition and microbial metabolic variation (explaining 80% and 47% of their variance, respectively), whereas DOC degradation and RDOC were more strongly associated with microbial metabolic variation than with community composition (*P* < .001 for both paths). In contrast, the direct statistical paths from community composition to DOC loss were weak and nonsignificant. These results are consistent with the interpretation that salinity-associated differences in carbon-processing outcomes were more closely related to shifts in microbial metabolic state than to taxonomic turnover alone, in line with the broader view that functional traits and metabolic capacities can better predict ecosystem processes than taxonomic identity [112, 127, 128]. In our experiment, bacterial community composition varied with salinity, but DOC degradation was more strongly associated with microbial metabolic activity, whereas the direct statistical effects of community composition were weak and nonsignificant. This pattern is consistent with the view that salinity-associated differences in carbon processing may involve shifts in microbial metabolic state, potentially linked to physiological stress and osmoadaptation [112, 129, 130]. Temporal dynamics and machine-learning analyses provide additional insight into the bacterial families most strongly associated with these processes. Overall community structure was relatively stable: diversity changed little in the LS group and decreased (mainly in richness) under HS (*P* < .001; Supplementary Figs. S15–S19); Proteobacteria, Bacteroidota, and Actinobacteriota remained dominant throughout. However, random forest models identified particular bacterial families as strong predictors of DOC degradation patterns (Fig. 7c, d). In the HS group, early DOC loss was most strongly associated with halotolerant generalist taxa. Halomonadaceae emerged as the second most influential family in the HS model, and its temporal dynamics (high initial abundance followed by a transient bloom around day 10) coincided with the rapid DOC decline phase (*P* < .001). This pattern is consistent with the ecology of Halomonadaceae (e.g. *Halomonas*), which are commonly described as opportunistic generalists capable of exploiting readily available substrates under salt stress [131, 132]. As incubation progressed, a succession toward more specialized consumers became apparent. Hyphomonadaceae ranked as the strongest predictor of DOC degradation under both salinity regimes (Fig. 7c, d) and increased significantly over time in both LS and HS treatments (*P* < .05), suggesting a key role during later stages of DOM degradation. This is consistent with studies indicating that Hyphomonadaceae (e.g. *Hyphomonas*) are surface-associated degraders of hydrocarbons and biopolymers and may contribute disproportionately to the processing of more refractory or particle-associated DOM once labile fractions are depleted [133, 134]. Together, this apparent succession from early-blooming generalists to later-acting, often lower-abundance specialists is consistent with the possibility that “rare” or late-successional taxa may contribute disproportionately to carbon turnover, and that focusing solely on initially abundant or dominant community members may underestimate their potential influence on DOC fate [37, 135, 136]. At the same time, these taxon-level patterns need to be interpreted together with the incubation-derived carbon-processing outcomes and the SEM results. Overall, the incubation-based carbon-processing patterns, DOM compositional trajectories, and SEM results were broadly consistent in indicating that, under the standardized nutrient-replete incubation conditions used here, higher salinity was associated with reduced residual carbon preservation and a stronger shift toward oxidative DOC processing. In the present study, the incubation-derived BDOC/RDOC shifts, together with the associated metabolomic patterns, provided the strongest process-relevant evidence for differences in DOC/RDOC outcomes under the studied conditions. The SEM was also consistent with the interpretation that these carbon-processing differences were more closely associated with microbial metabolic variation than with taxonomic turnover alone. Accordingly, salinity-associated community and network restructuring should be interpreted as the ecological backdrop to these process-level differences, rather than as the sole basis for inferring DOC/RDOC fate.

### Study limitations

Several limitations constrain the interpretation of this study. Taken together, these limitations mean that the present study should be interpreted as providing process-relevant, condition-specific evidence from Yuncheng Salt Lake, rather than a direct mechanistic demonstration or a broadly generalizable account of salinity effects across saline lakes. First, our inferences are based on a single lake system, a limited temporal window, and a comparison between only two contrasting salinity regimes within Yuncheng Salt Lake. Although this within-lake design provides a strong framework for spatial comparison under a shared regional setting, it does not resolve continuous salinity responses, seasonal variability, or broader differences among inland saline lakes. In particular, saline lakes can differ substantially in ionic composition, alkalinity, DOM sources, hydrology, and photochemical setting, all of which may influence DOM processing and microbial assembly. Accordingly, the present results should be interpreted primarily within the environmental context represented here, rather than as directly generalizable across saline lakes more broadly. Second, salinity covaried with other environmental attributes in this system, including chemistry and habitat context. Although targeted variation partitioning showed that salinity retained the largest unique explained fraction, co-varying chemistry and habitat type also contributed significantly to bacterial community variation. Thus, our dataset supports the interpretation that salinity was an important and independently significant factor in this system, but not that it acted in isolation or as the sole determinant of the observed ecological and biogeochemical differences. Third, although the controlled incubation experiment, two-pool DOC modeling, metabolomic evidence, and integrative statistical analyses provide process-relevant support for salinity-associated differences in carbon processing, they do not constitute a direct mechanistic demonstration at the gene, enzyme, or *in situ* flux level. The microbial evidence is based primarily on 16S rRNA profiling and untargeted metabolomics, with incomplete metabolite annotation, and key microbial groups such as archaea, fungi, and viruses were not explicitly included. In addition, the bacteria–DOM network was inferred from correlation structure and should therefore be interpreted as an exploratory association network rather than direct evidence of ecological interaction, whereas the random forest and SEM analyses identify statistical associations and relative predictor importance rather than strictly independent or causal effects. Therefore, the mechanistic interpretations in this study should be regarded as experimentally and statistically supported process-level inferences, rather than direct proof of the underlying functional pathways. Fourth, the incubation experiment was intentionally conducted under standardized nutrient-replete conditions and therefore does not isolate the effect of salinity under ambient *in situ* nutrient availability. Although the source chemistry of the water and interface samples did not support systematically stronger nutrient limitation in the high-salinity groups, nutrient amendment may still have influenced the absolute magnitude of DOC turnover and microbial carbon-use efficiency. Moreover, the incubation source material for each combination of salinity regime and sample matrix was generated by compositing five field replicates prior to establishing parallel microcosms. This design improved treatment standardization, but it also reduced our ability to evaluate site-level heterogeneity in incubation responses. Consequently, the incubation results should be interpreted as comparisons among composited source samples from contrasting salinity regimes under nutrient-replete laboratory conditions, and future work with ambient-nutrient and reduced-amendment controls, together with non-composited replicate incubations, will be needed to more fully separate salinity, nutrient, and site-level effects. Fifth, RDOC in this study is operationally defined as the residual DOC fraction remaining after 100 days of incubation, as estimated by the two-pool model. It therefore represents relative DOC persistence at the experimental timescale under the imposed incubation conditions, rather than direct evidence of long-term intrinsic recalcitrance or multiyear persistence *in situ*. Relatedly, we did not directly quantify CO_2_ or CH_4_ production or whole-system carbon balance. Thus, any implication for carbon sequestration or greenhouse-gas release should be interpreted as inferential and conditional on the present evidence, rather than as an empirically demonstrated system-level outcome.

## Conclusions

Overall, our results indicate that, within Yuncheng Salt Lake, salinity was an important factor associated with variation in DOC degradation and RDOC preservation beyond taxonomic composition alone. Lower-salinity conditions were associated with greater preservation of complex organic matter, whereas hypersaline conditions were associated with more extensive oxidation and utilization of compounds that might otherwise persist, consistent with lower RDOC preservation under high salinity. Under the standardized nutrient-replete incubation conditions used here, these contrasting outcomes were consistently accompanied by shifts in BDOC/RDOC partitioning, DOM composition, metabolomic profiles, and stronger associations of DOC degradation and RDOC dynamics with microbial metabolic variation than with community composition alone. Together, these findings provide process-level, rather than direct mechanistic, evidence that contrasting salinity regimes within Yuncheng Salt Lake were associated with differences in microbial carbon-processing patterns. Future work combining ambient-nutrient controls, direct carbon-flux measurements, and process-resolved multi-omics will be needed to better resolve how salinity influences microbial carbon processing and its implications for carbon preservation in saline-lake systems.

## Supplementary Material

wrag107_Supplementary_Materials

## Data Availability

The raw sequencing data for bacterial communities have been deposited in the National Center for Biotechnology Information (NCBI) Sequence Read Archive (SRA) under BioProject accession numbers PRJNA1394519 (field sampling) and PRJNA1394533 (incubation experiment). Metabolomics data generated in this study are available in OMIX (China National Center for Bioinformation/Beijing Institute of Genomics, Chinese Academy of Sciences) under accession numbers OMIX013981 (field sampling) and OMIX013983 (incubation experiment) via the OMIX portal (https://ngdc.cncb.ac.cn/omix).

## Code availability

All R scripts used for statistical analyses and figure generation in this study are publicly available in the GitHub repository (https://github.com/2766311507-dotcom/saline-lake-omics-R).
