# Supplementary material for: Contrasting salinity regimes reshape microbe–DOM coupling and reduce recalcitrant dissolved organic carbon preservation in a salt lake
Source: ISME J. 2026 May 7;20(1):wrag107. doi: 10.1093/ismejo/wrag107 (PMC13200285; doi:10.1093/ismejo/wrag107)
Supplement: wrag107_Supplementary_Materials [file wrag107_supplementary_materials.docx]

Supplementary material for

**Contrasting salinity regimes reshape microbe-DOM coupling and reduce recalcitrant dissolved organic carbon preservation in a salt lake**

Xiding Wang,Yang Liu*, Xinyue Yang, Ruikai Zhang, Xudong Liu, Fangru Nan, Qi Liu, Junping Lv, Jia Feng, Shulian Xie

Shanxi Key Laboratory for Research and Development of Regional Plants, School of Life Science, Shanxi University, Taiyuan 030006, China

* Corresponding E-mail: Yang Liu: [liuy@sxu.edu.cn](mailto:liuy@sxu.edu.cn;)

**Contents of this file:**

- **Texts: 5**
- **Figures: 19**
- **Tables: 15**
- **References: 13**

**Supplementary Methods 1.** DNA extraction, PCR amplification, and library preparation

Total genomic DNA was extracted from all water, water-sediment interface, and sediment samples using the E.Z.N.A.^TM^ Mag-Bind Soil DNA Kit (Omega, M5635-02, USA) following the manufacturer’s protocol, with minor adaptations for high-salinity matrices. Briefly, approximately 0.5 g of homogenized sediment or the equivalent volume of pelleted biomass from water and interface samples was transferred into the lysis tubes supplied with the kit. Samples were subjected to mechanical disruption and chemical lysis, followed by binding of nucleic acids to magnetic beads, multiple washing steps to remove salts and potential inhibitors, and final elution of DNA in nuclease-free buffer. Extraction blanks were included in each batch to monitor possible contamination. DNA yield and purity were assessed using a Qubit 4.0 fluorometer (Thermo Fisher Scientific, USA), and only extracts with sufficient concentration and without obvious signs of degradation were used for downstream amplification.

The bacterial community was characterized by amplifying the V3-V4 hypervariable region of the 16S rRNA gene. PCR reactions were performed with the primer pair 341F (CCTACGGGNGGCWGCAG) and 806R (GACTACHVGGGTATCTAATCC), which has been widely adopted for bacterial diversity studies. Amplifications were carried out in 30 μL reaction volumes containing 2 × Hieff® Robust PCR Master Mix (Yeasen, 10105ES03, China), 10 ng of template DNA, 0.2 μM of each primer, and nuclease-free water to volume. All samples were amplified in triplicate to minimize PCR stochasticity, and negative controls (no-template reactions) were run alongside[1].

PCR was performed in a thermal cycler (Applied Biosystems 9700, USA) using a two-step annealing program optimized for these primers. The cycling conditions were as follows: an initial denaturation at 95 °C for 3 min; 5 cycles of denaturation at 95 °C for 30 s, annealing at 45 °C for 30 s, and extension at 72 °C for 30 s; followed by 20 cycles of denaturation at 95 °C for 30 s, annealing at 55 °C for 30 s, and extension at 72 °C for 30 s; and a final elongation at 72 °C for 5 min. The use of a lower annealing temperature in the initial cycles facilitated primer binding across diverse bacterial templates, while the subsequent higher annealing temperature improved specificity.

Following amplification, PCR products from replicate reactions of the same sample were pooled and checked by electrophoresis on 2% (w/v) agarose gels in TBE buffer. Gels were stained and visualized under UV illumination to confirm the presence of a single band of the expected size (~460 bp) and to verify the absence of amplicons in negative controls. Samples that showed weak or smeared bands were re-amplified or excluded from library preparation.

Pooled amplicons were purified using Hieff NGS^TM^ DNA Selection Beads (Yeasen, China) according to the manufacturer’s instructions to remove primer dimers, residual nucleotides, and other contaminants. The concentration of the cleaned PCR products was re-measured with the Qubit fluorometer, and equimolar amounts of each sample were combined to construct the sequencing library. Adapter ligation and index incorporation followed the standard Illumina workflow used by the sequencing provider. The final library quality (size distribution and concentration) was evaluated before high-throughput sequencing[2].

After sequencing, paired-end Illumina reads were merged with PEAR (v0.9.8) based on their overlap[2]. The resulting FASTQ data were quality-processed to obtain per-sample FASTA and QUAL files for downstream analyses. High-quality reads (“effective tags”) were clustered into operational taxonomic units (OTUs) at a 97% similarity threshold using USEARCH (v11.0.667). Chimeric sequences and singleton OTUs (supported by only one read) were discarded, and the remaining sequences were assigned back to individual samples according to the OTU table[3, 4]. For each OTU, the most abundant tag was chosen as the representative sequence. Taxonomic annotations were determined by comparing bacterial representative sequences against the RDP database (v11.4)[5].

**Supplementary Methods 2.** Metabolomic extraction and LC-MS analysis

All samples used for metabolomic analysis were stored at -80 °C and thawed slowly on ice prior to processing to minimize thermal degradation of labile metabolites. For water-based and interface samples, a pre-chilled solvent mixture consisting of acetonitrile and methanol (1:4, v/v) containing internal standards was added immediately after thawing. The samples were thoroughly mixed on a high-speed shaker to promote efficient precipitation of macromolecules and release of low-molecular-weight compounds into the extraction solvent. After shaking, the mixtures were centrifuged at 3000 rpm for 10 min at 4 °C, and the resulting supernatants were transferred to clean tubes and evaporated under a gentle nitrogen stream. The concentrates were reconstituted in a methanol-water solution (7:3, v/v), vortexed and briefly sonicated in an ice bath to ensure complete dissolution of metabolites, and centrifuged again at high speed to remove any remaining particulate matter. The clarified extracts were transferred to LC-MS autosampler vials for instrumental analysis.

Sediment samples required a slightly different extraction workflow to account for their high matrix complexity. Approximately 250 mg of homogenized sediment was combined with methanol-water (7:3, v/v) containing internal standards. The mixture was vortexed thoroughly and subjected to ultrasonic disruption on ice to enhance extraction efficiency. Following sonication, the extracts were held at -20 °C for approximately 30 min to improve protein precipitation and phase separation. The samples were then centrifuged at 12,000 rpm for 10 min at 4 °C, and the resulting supernatants were filtered through 0.22 μm PTFE membranes to remove fine particulates. The filtrates were transferred to LC-MS vials for immediate acquisition or refrigerated briefly prior to analysis.

Chromatographic separation of metabolites was carried out using a reverse-phase UHPLC system equipped with an ACQUITY Premier HSS T3 column (2.1 × 100 mm, 1.8 µm; Waters). To achieve broad chemical coverage, each extract was analyzed twice, once under positive electrospray ionization conditions and once under negative mode. The mobile phases consisted of water with 0.1% formic acid and acetonitrile with 0.1% formic acid. A multistage gradient was used to elute metabolites with varying polarity, beginning with a low organic content, gradually increasing to a high-acetonitrile fraction, and then returning to initial conditions for column re-equilibration. The chromatographic system was operated at a constant flow rate of 0.4 mL min^-1^, with the column maintained at 40 °C to ensure stable retention times. A 4 µL injection volume was used for all analyses.

Mass spectrometric detection was performed on a high-resolution instrument operated in electrospray ionization mode and configured to alternate between full-scan MS and data-dependent MS/MS fragmentation for structural elucidation. Full-scan spectra were collected across an m/z range of 75-1000 at a resolving power of 35,000. Ionization parameters were optimized for both ion modes, including spray voltage, sheath and auxiliary gas flow, and source temperatures, to ensure robust and reproducible ion generation. Fragmentation energies were applied in a stepped manner to capture complementary MS/MS spectra across a range of compound classes. Dynamic exclusion was enabled to avoid repeated fragmentation of highly abundant ions, allowing the instrument to sample a greater diversity of metabolites. The MS signals were acquired using an intensity threshold appropriate for untargeted metabolomics to ensure the capture of both high- and moderate-abundance ions.

**Supplementary Methods 3 Detailed incubation design and standardization**

To examine DOM transformation under controlled laboratory conditions, incubation systems were established for each salinity regime and sample matrix. For each salinity × matrix group, five field replicates were composited to generate a representative source sample, which was then divided into three parallel incubation microcosms. Therefore, the incubation replicates represent parallel responses of the same composite source rather than independent field-derived environmental replicates.

Incubations were conducted in acid-cleaned and combusted (450 °C for 4 h) 10 L transparent borosilicate glass carboys sealed with PTFE-lined caps to minimize contamination from leachable organic compounds. Microbial inocula were prepared by passing fresh samples through a 53 μm mesh to remove large particles, followed by gentle mixing to obtain microbial suspensions. DOM-containing water was prepared by filtering the corresponding water samples through 0.22 μm membranes to remove most microbial cells while retaining dissolved organic matter. Each 10 L incubation system consisted of 8 L of 0.22 μm-filtered DOM-rich water and 2 L of microbial suspension. For sediment-derived treatments, DOM and microbial fractions were first extracted from the sediment matrix and then recombined at the same volume ratio.

Salinity in the low-salinity and high-salinity treatments was adjusted to 30 ppt and 90 ppt, respectively, using sterile concentrated salt solutions prepared from the corresponding lake brines. These target values were set slightly above the mean field salinities (25 and 80 ppt) to compensate for minor dilution associated with inoculation and repeated subsampling over the 100-day incubation, while remaining within the environmentally realistic range of the Yuncheng Salt Lake system. The purpose of this adjustment was to maintain stable and clearly separated salinity regimes throughout the experiment rather than to impose unrealistically extreme salinity stress.

To minimize unequal nutrient limitation among microcosms and standardize comparisons among treatments, NaNO_3_ and KH_2_PO_4_ were added to establish a nutrient-replete condition based on the day-0 DOC concentration and a target molar C:N:P ratio of 106:16:1. Treatment-specific final additions of nitrate-N and phosphate-P are reported in Table S1 in both mg L^-1^ and μM, together with the equivalent mass additions of NaNO_3_ and KH_2_PO_4_. Accordingly, this incubation design was intended to compare DOC/RDOC dynamics among source samples from contrasting salinity regimes under nutrient-replete conditions, rather than to reproduce ambient in situ nutrient availability.

Incubations were maintained under a 12 h light:12 h dark photoperiod in transparent borosilicate carboys at a controlled light intensity of 50 μmol photons m^-2^ s^-1^. Because transparent vessels were used, some photochemical alteration of DOM may have occurred during the incubation. However, all treatments were exposed to the same vessel type and identical light conditions, so any photochemical contribution was standardized across treatments and cannot explain the consistent directional differences observed between the low- and high-salinity groups.

**Supplementary Methods 4.** iCAMP-Based Inference of Bacterial and DOM Community Assembly Processes

Community assembly processes of bacterial communities were quantified using the iCAMP framework implemented in the R package “iCAMP”[6]. OTU tables, phylogenetic trees and sample metadata were first matched, and low-abundance taxa were removed prior to analysis. Taxa were grouped into phylogenetic bins based on pairwise distances using a threshold distance (ds = 1.6), and the presence of phylogenetic signal within each bin was evaluated using function *ps.bin*. Null-model analyses were then conducted with function *icamp.big* using 1,000 randomizations to compute the β-nearest taxon index (βNRI, using bMPD as the phylogenetic metric) and Raup-Crick dissimilarity for Bray-Curtis (RC_bray). For each pairwise community comparison, βNRI and RC_bray values were used to assign ecological processes—homogeneous selection, heterogeneous selection, dispersal limitation, homogenizing dispersal, or drift/undominated—following the standard iCAMP decision criteria. The relative importance of each process was obtained using function *icamp.bins*, which aggregates process assignments across bins weighted by their relative abundances. Abundance-weighted global βNRI and RC_bray were also extracted for each sample pair and further summarized within and between salinity groups. Bin-level phylogenetic trees and process-contribution matrices were generated for downstream comparison and visualization.

For DOM, a transformation-weighted characteristic dendrogram (TWCD) was constructed prior to assembly analysis[7]. FT-ICR-MS peak lists were merged and processed using the R package “ftmsRanalysis” to compute molecular indices (e.g., NOSC, AImod, DBE) and Van Krevelen-based structural classes. Biochemical transformations among molecular formulae were identified by matching exact mass differences to a curated transformation database, and a transformation network was constructed and converted into a transformation distance matrix using the R packages “igraph” and “vegan”. This network-based distance was combined with a molecular property distance matrix to produce a weighted dissimilarity matrix, from which a UPGMA tree was generated using function *hclust* and converted into a dendrogram with function *as.phylo* in the R package “phangorn”, yielding the TWCD.

The TWCD and DOM presence-absence matrix were then used as inputs to the iCAMP workflow to infer DOM assembly processes in a manner analogous to the bacterial analysis. DOM molecular formulae were binned along the TWCD, the presence of dendrogram-based signal within bins was assessed, and null-model calculations with function *icamp.big* were used to derive βNRI and RC_bray for each bin. Ecological processes were assigned for each pairwise comparison using the same βNRI and RC_bray thresholds, and their relative contributions were summarized across bins and treatments using function *icamp.bins*. This approach allowed DOM assembly processes to be evaluated using a transformation-informed dendrogram that reflects both biochemical connectivity and molecular characteristics.

**Supplementary Methods 5.** Random forest analysis

Two random forest analyses were conducted in R using the “randomForest” package. The first analysis was designed to identify metabolites most strongly associated with DOM molecular variation across sample matrices, whereas the second was used to evaluate the relative contributions of dominant bacterial families to DOC degradation-related variables in the incubation experiment.

In the first analysis, DOM molecular profiles were first converted to relative abundances and Hellinger-transformed using the *decostand* function in the “vegan” package. Bray-Curtis dissimilarities were then calculated with *vegdist*, and principal coordinates analysis (PCoA) was performed using *cmdscale*; the first principal coordinate (PCoA1) was used as the response variable in the random forest model. Metabolite data were log10-transformed [log10(x + 1)] and z-score standardized prior to modeling. Samples shared between the DOM and metabolite datasets were retained, and the combined dataset was randomly split into training and test sets at a 70:30 ratio using a fixed random seed (set.seed (123)). Random forest models were fitted with ntree = 500, importance = TRUE, and the default mtry setting of the “randomForest” package. Model performance was evaluated using mean squared error (MSE) and test-set *R*^2^. Predictor importance and permutation-based significance were further evaluated using the “rfPermute” package with ntree = 500, nrep = 1000, and set.seed (123). Overall model significance was assessed using the “A3” package with p.acc = 0.001.

In the second analysis, the top 20 most abundant bacterial families were used as predictors to model DOC degradation-related response variables in the LS and HS incubation systems. Response variables included BDOC, DOC, and ΔDOC, and separate models were constructed for each response variable within each salinity treatment. Samples shared between the bacterial-family and DOC-variable datasets were retained. For each model, samples were randomly divided into training and test sets at a 70:30 ratio using set.seed (123), and random forest models were fitted with ntree = 500, importance = TRUE, and the default mtry setting. Variable importance and permutation-based significance were assessed using rfPermute with ntree = 500, nrep = 1000, and set.seed (123), and overall model significance was evaluated using “A3” with p.acc = 0.001.

Predictor collinearity was not explicitly screened prior to model fitting in either analysis. Therefore, variable-importance values were interpreted as relative importance rankings within the full predictor set rather than as strictly independent effect estimates.


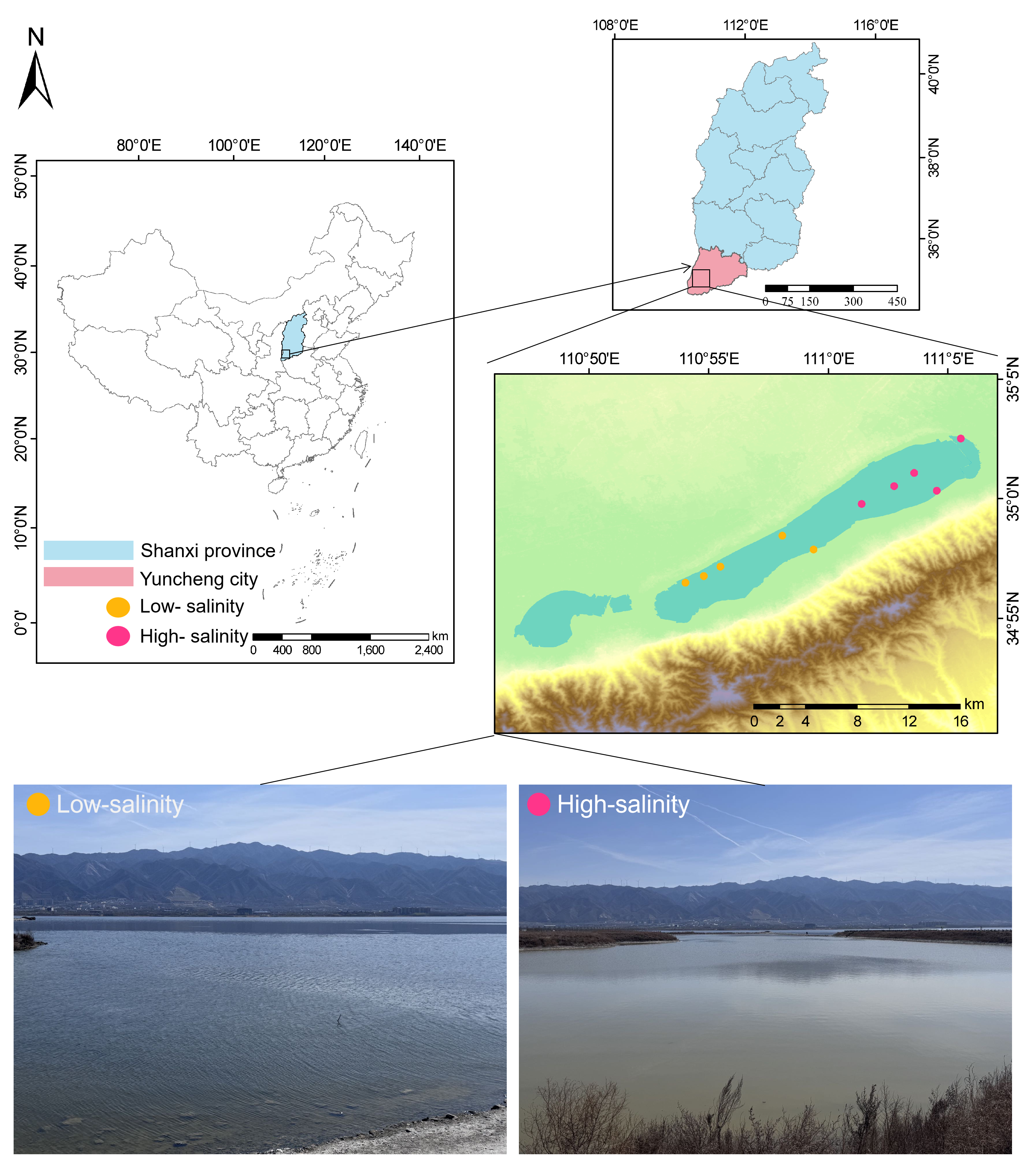


**Supplementary Figure 1. Geographic location of the study area and sampling design in Yuncheng Salt Lake, Shanxi Province, China.** The map shows the position of Yuncheng City within Shanxi Province and the spatial distribution of sampling sites across Yuncheng Salt Lake. The lake water exhibits two distinct salinity regimes: a low-salinity zone (~25 ppt) and a high-salinity zone (~80 ppt). To obtain a comprehensive characterization of biogeochemical patterns and to avoid matrix-specific bias, samples were collected from three environmental media within each salinity regime: water, water-sediment interface, and sediment (0-15 cm). A total of ten sampling sites were established along the longitudinal salinity gradient, including five sites in the low-salinity zone (orange markers) and five sites in the high-salinity zone (pink markers)


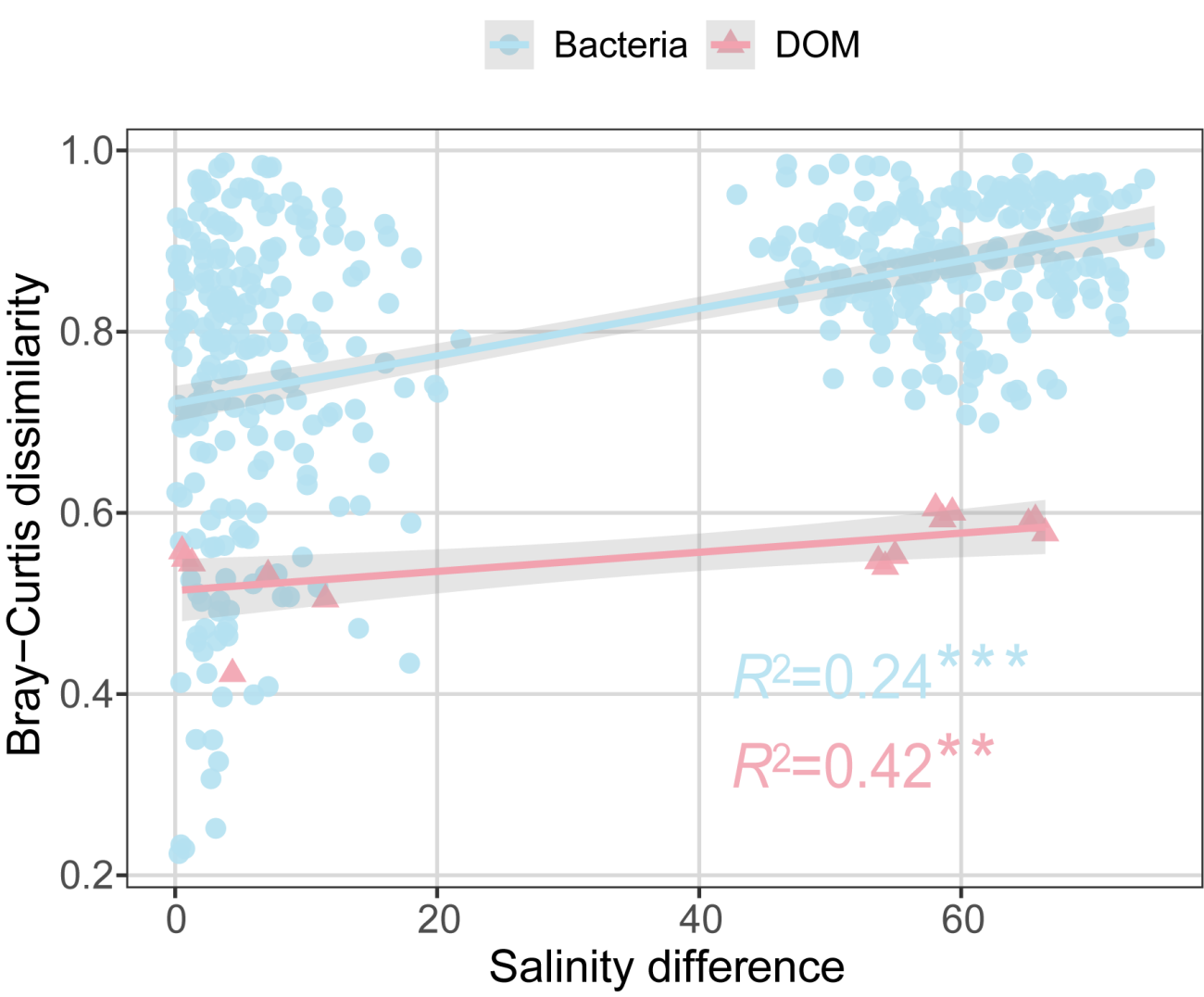


**Supplementary Figure 2. Bray-Curtis dissimilarity of bacterial communities and DOM molecular composition as a function of salinity difference.** Linear regressions were fitted separately for bacteria and DOM to quantify salinity-driven distance-decay patterns. Shaded of grey represent 95% confidence intervals. To reduce the likelihood of false positives, *P* values from all regression analyses were corrected using the Benjamini-Hochberg false discovery rate (FDR) procedure. ***P* < 0.01, and ****P* < 0.001.

**
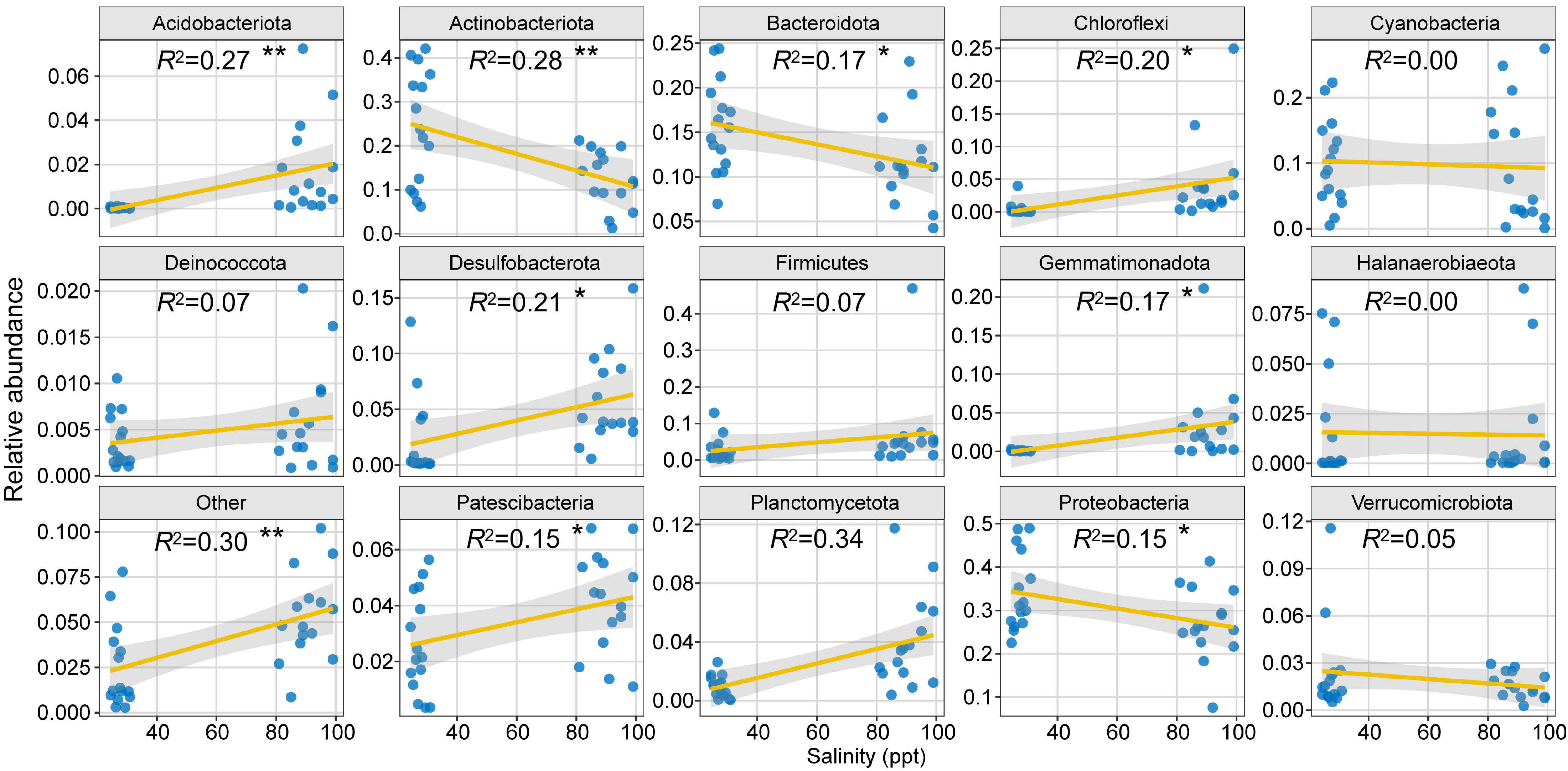
Supplementary Figure 3. Linear relationships between salinity and the relative abundance of major bacterial phyla.** Linear regressions examining how salinity influences the relative abundance of the top 15 dominant bacterial phyla. Shaded of grey represent 95% confidence intervals. To reduce the likelihood of false positives, *P* values from all regression analyses were corrected using the Benjamini-Hochberg false discovery rate (FDR) procedure. **P* < 0.05, ***P* < 0.01.

**
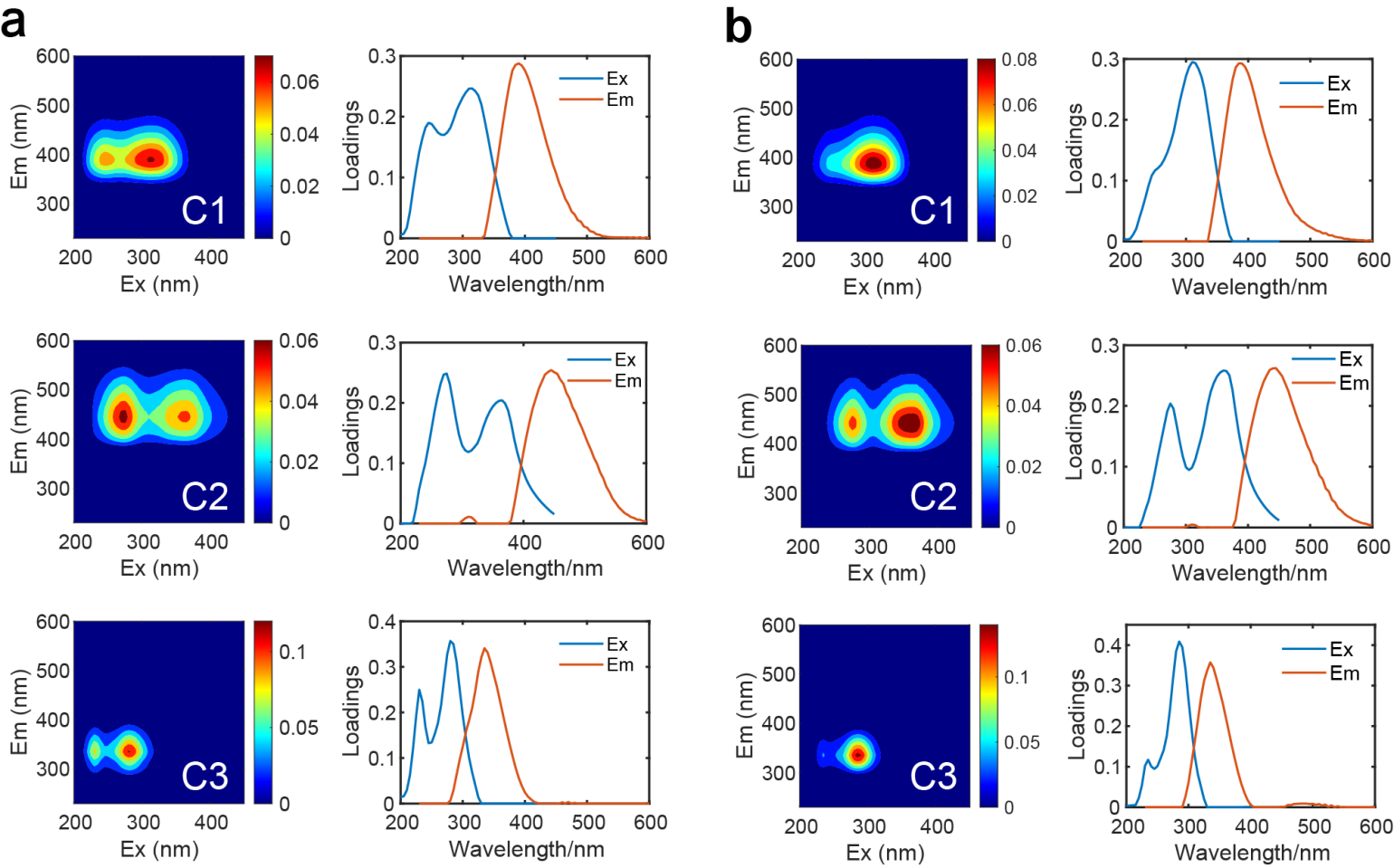
Supplementary Figure 4. Fluorescence characterization of DOM based on excitation-emission matrix (EEM) spectroscopy and PARAFAC modelling between the low-salinity (LS) and high-salinity (HS) groups.** **a** Identified PARAFAC components in the low-salinity (LS) group, including C1, C2, and C3. C1 shows two characteristic peaks at Ex/Em = 245/390 nm (peak A) and 315/390 nm (peak M ), both associated with humic-like fluorophores indicative of allochthonous humic and fulvic substances. C2 exhibits two humic fluorescence peaks at Ex/Em = 275/445 nm and 365/445 nm, corresponding to the peak C, representing terrestrial humic substances commonly found in natural waters. C3 displays two protein-like peaks at Ex/Em = 230/335 nm and 280/335 nm, characteristic of the peak T, indicating autochthonous tryptophan-like fluorescence. In the LS group, the relative contributions of the three components were C1: 38.1%, C2: 21.5%, and C3: 40.4%. **b** PARAFAC components identified in the high-salinity (HS) group. C1 contains a single humic-like peak M at Ex/Em = 310/385 nm. C2 features two peaks at Ex/Em = 275/445 nm and 360/445 nm, corresponding to the peak C (terrestrial humic substances). C3 shows two protein-like peak T at Ex/Em = 235/335 nm and 285/335 nm, representing tryptophan-like fluorescence. In the HS group, the relative contributions of the components were C1: 41.7%, C2: 42.7%, and C3: 15.6%.


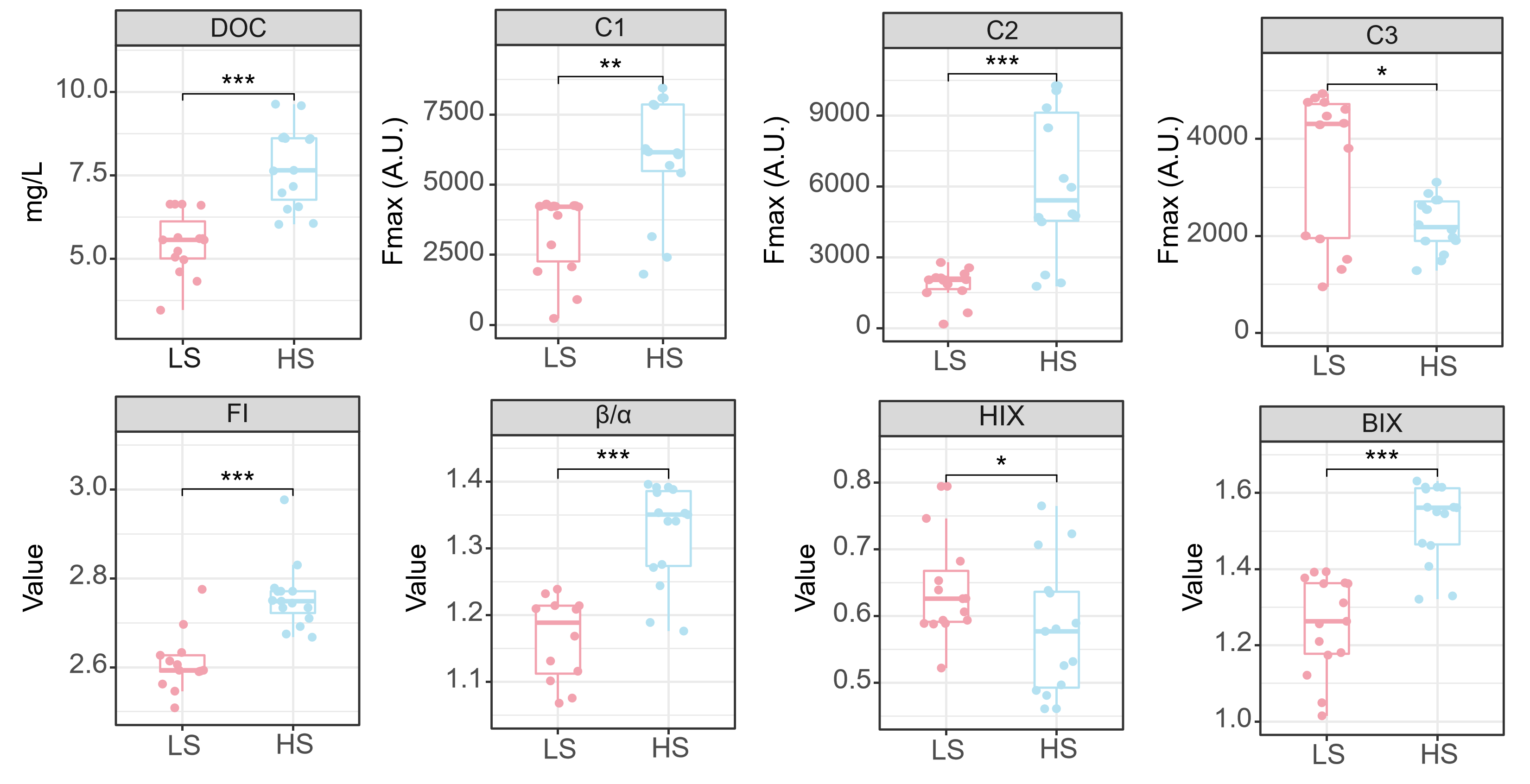


**Supplementary Figure 5. Comparison of DOC, PARAFAC component fluorescence intensities (Fmax of C1, C2, and C3), and four fluorescence indices (FI, β/α, HIX, and BIX) between the low-salinity (LS) and high-salinity (HS) groups.** Statistical significance was assessed using two-sided t-tests. **P* < 0.05, ***P* < 0.01, and ****P* < 0.001

**
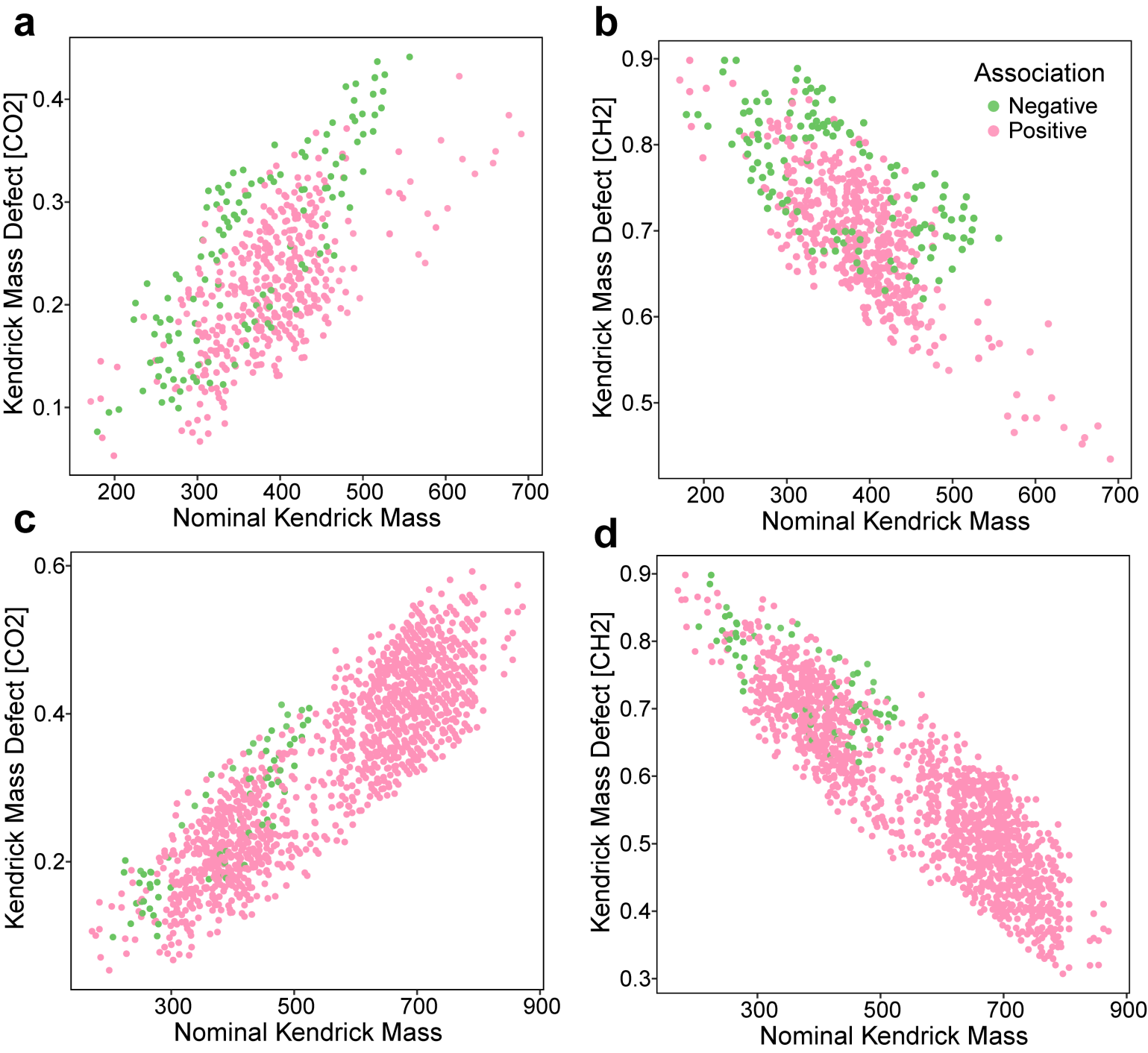
**

**Supplementary Figure 6. Kendrick mass defect (KMD) patterns of DOM molecules under low- and high-salinity conditions.** a, b KMD distributions of DOM molecular formulae in the low-salinity (LS) group based on CO_2_-normalized (a) and CH_2_-normalized (b) Kendrick mass defect. c, d Corresponding KMD distributions for the high-salinity (HS) group using CO_2_-KMD (c) and CH_2_-KMD (d). For each molecule, the relationship between KMD values and salinity was assessed, and points are colored to indicate positive (pink) or negative (green) correlation


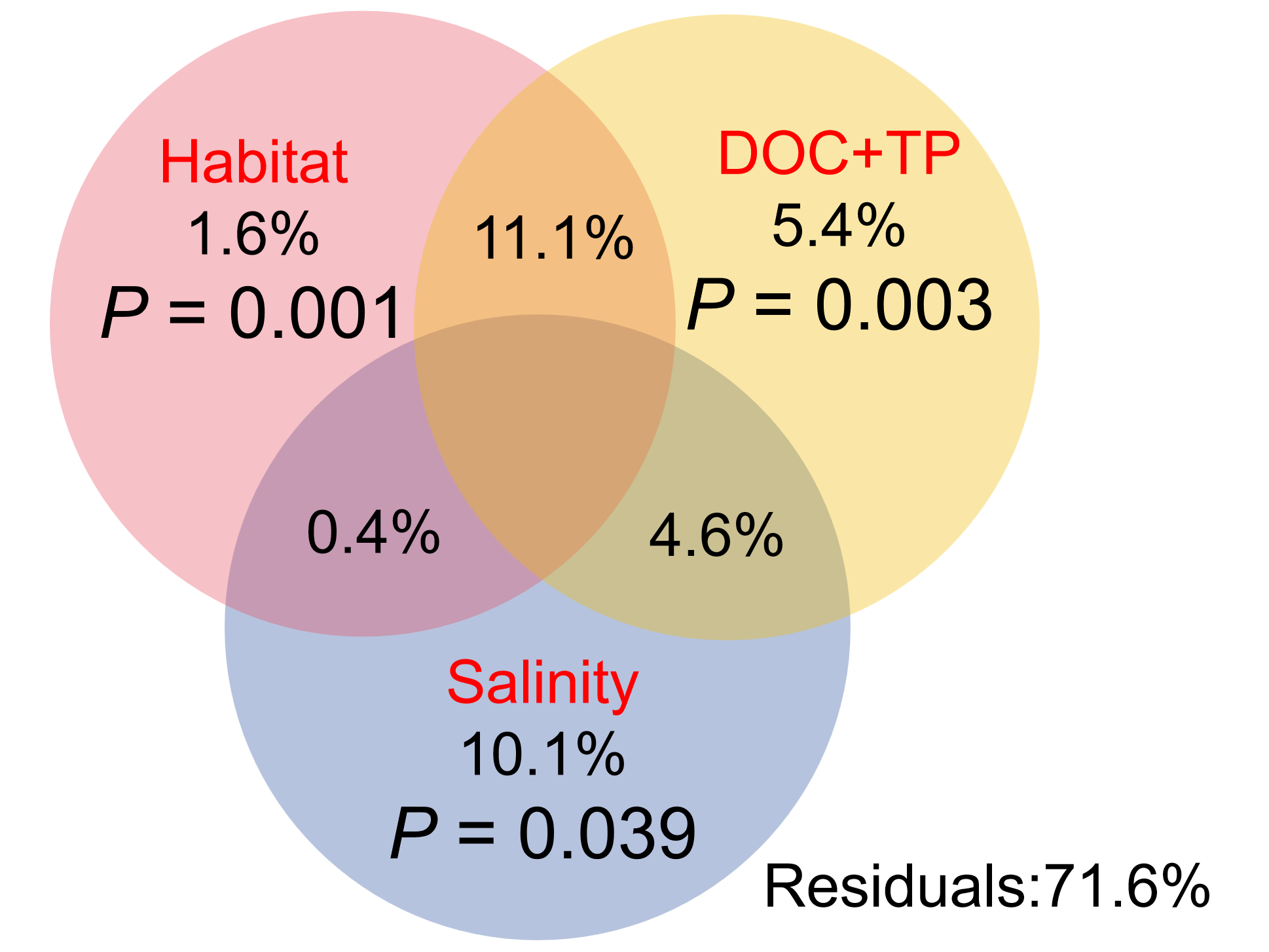


**Supplementary Figure 7. Targeted variation partitioning analysis of bacterial community variation explained by salinity, co-varying chemistry, and habitat type.** The Hellinger-transformed OTU matrix was partitioned into three explanatory sets: salinity (SAL), co-varying chemistry (DOC + TP), and habitat type. Values in non-overlapping regions indicate the unique adjusted fractions explained by each set, whereas values in overlapping regions indicate shared fractions. Salinity explained the largest unique fraction of bacterial community variation (10.11%, p = 0.001), followed by DOC + TP (5.39%, p = 0.003) and habitat (1.61%, p = 0.039). Shared fractions were largest between habitat and DOC + TP (11.11%), followed by DOC + TP and salinity (4.55%), whereas the shared fraction between habitat and salinity was comparatively small (0.35%). Residuals accounted for 71.57% of the total variation. These results indicate that, although bacterial community structure reflected the joint influence of salinity, chemistry, and habitat context, salinity retained the strongest unique association among the explanatory sets tested here. Significance of the unique fractions was assessed by permutation tests based on partial RDA (999 permutations).

**
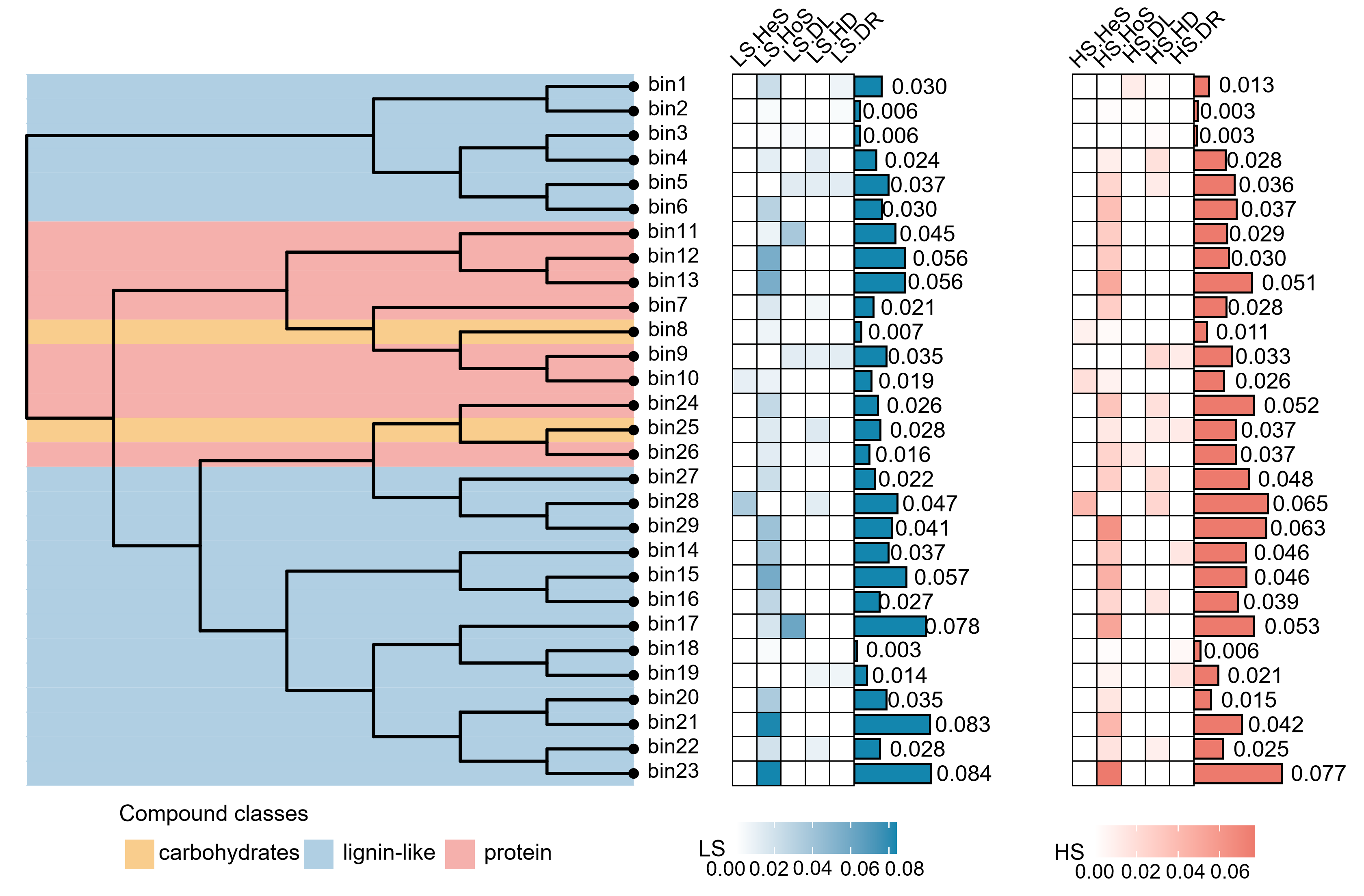
Supplementary Figure 8.** **iCAMP-based phylogenetic-binning analysis of DOM molecular assembly.** The left tree is colored by compound classes, and the adjacent heatmap show, for LS and HS, the relative importance of heterogeneous selection (HeS), homogeneous selection (HoS), dispersal limitation (DL), homogenizing dispersal (HD), and drift and others (DR) for each bin.

**
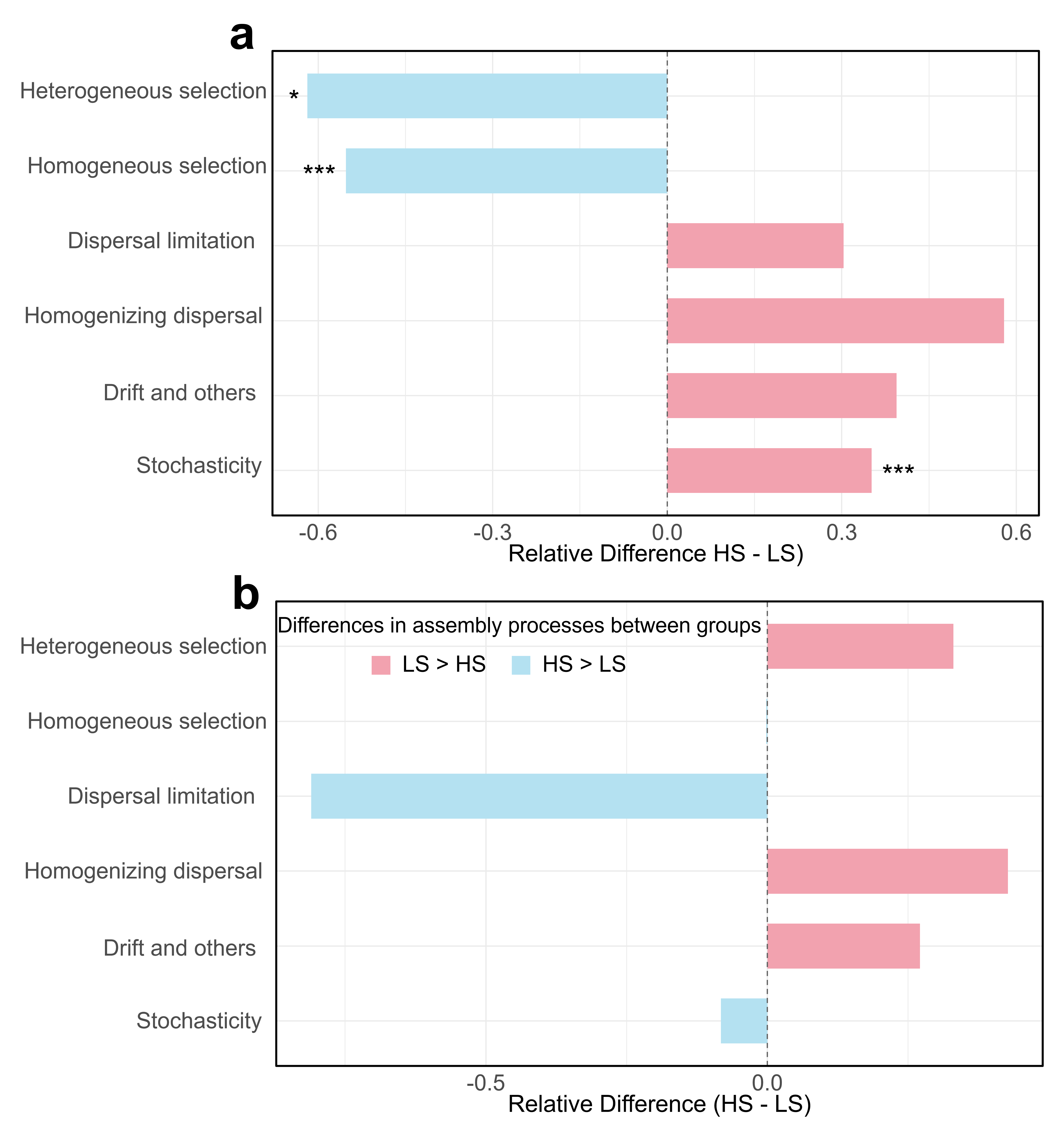
**

**Supplementary Figure 9. Differences in ecological assembly processes between low-salinity and high-salinity groups. a, b** Relative differences in the contributions of the five ecological processes to **bacterial communities** **(a)** and DOM molecular **(b)** assemblages between HS and LS groups. Bars to the right indicate higher contributions in HS, and bars to the left indicate higher contributions in LS; asterisks denote significant differences. Significance of assembly-process differences was assessed using permutation tests (iCAMP Compare), with *P*values based on random group-label shuffling. **P* < 0.05, and ****P* < 0.001.

**
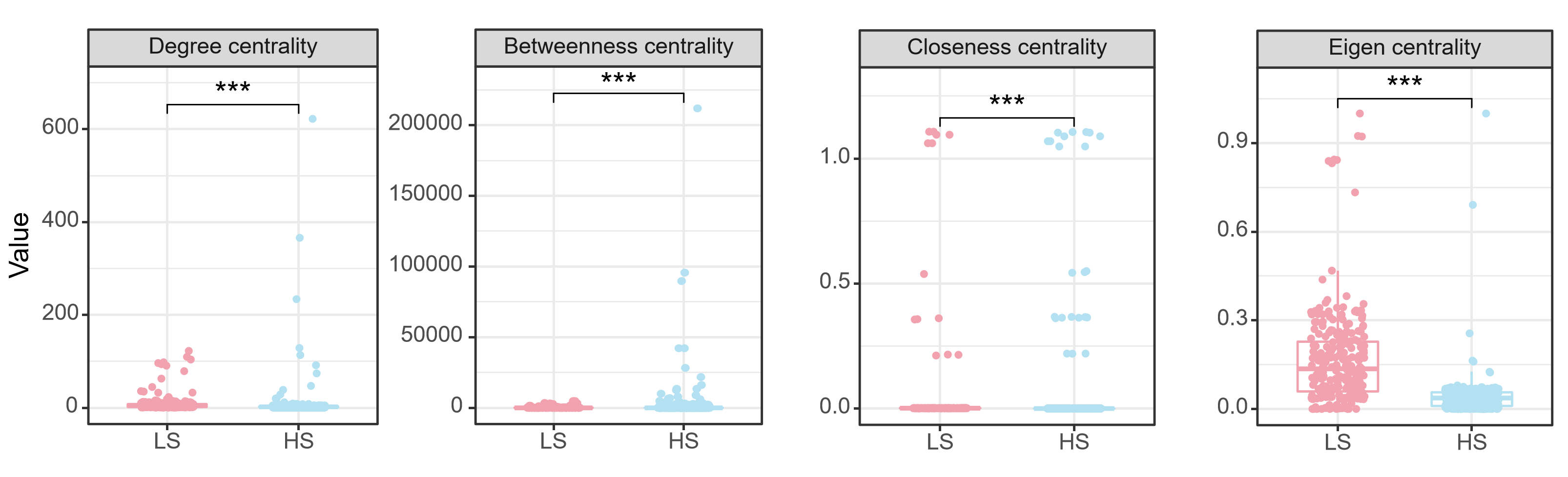
Supplementary Figure 10. Comparison of node centrality metrics between low-salinity (LS) and high-salinity (HS) co-occurrence networks.** Boxplots comparing four centrality metrics—degree, betweenness, closeness, and eigenvector centrality—between LS and HS networks. Statistical significance was evaluated using the Mann-Whitney U test, and asterisks indicate significant differences between salinity groups. ****P* < 0.001.


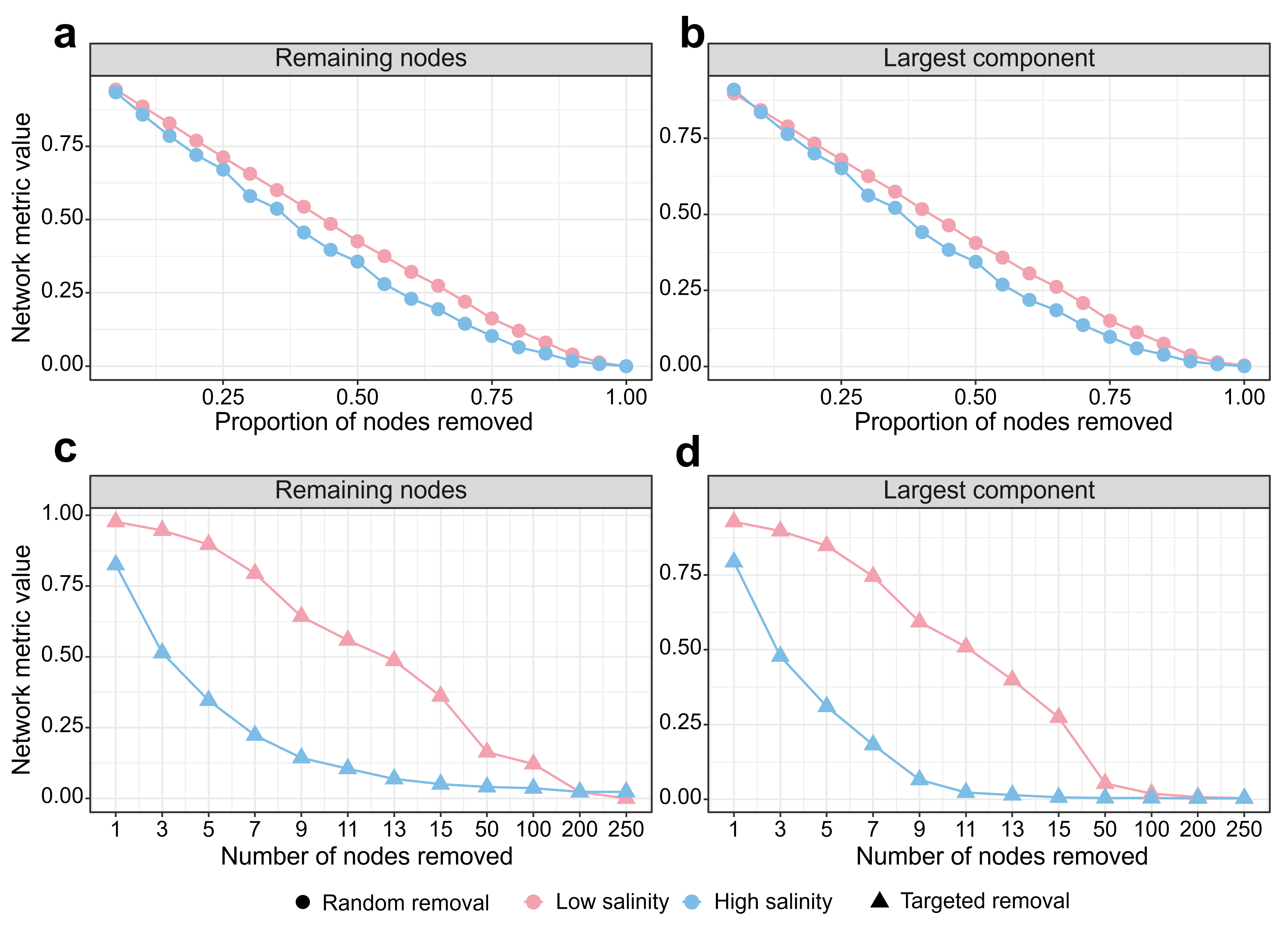
**Supplementary Figure 11. Robustness of low-salinity (LS) and high-salinity (HS) co-occurrence networks to random and targeted node removal. a,b** Robustness under random node removal. The fraction of remaining nodes **(a)** and the relative size of the largest connected component **(b)** were plotted as a function of the proportion of nodes removed. Both LS and HS networks decreased progressively with increasing node loss, but the HS network showed a more rapid decline, indicating lower structural robustness under random perturbations. **c, d** Robustness under targeted removal, in which nodes were removed sequentially from highest to lowest degree (i.e., hub-first attack). Declines in the proportion of remaining nodes **(c)** and the size of the largest component **(d)** were markedly faster in the HS network, demonstrating that the HS network is substantially more vulnerable to the removal of highly connected nodes. In contrast, the LS network retained larger connected substructures even after the removal of many hubs, reflecting higher tolerance to targeted disturbances. Together, these comparisons show that the LS network displays overall greater robustness—both to random loss of nodes and to deliberate removal of highly connected hubs—whereas the HS network is more fragile, exhibiting rapid fragmentation especially under targeted attacks. This suggests that microbial-DOM interactions in high-salinity environments rely more heavily on a small number of highly connected keystone nodes, making the HS network structurally less resilient

**
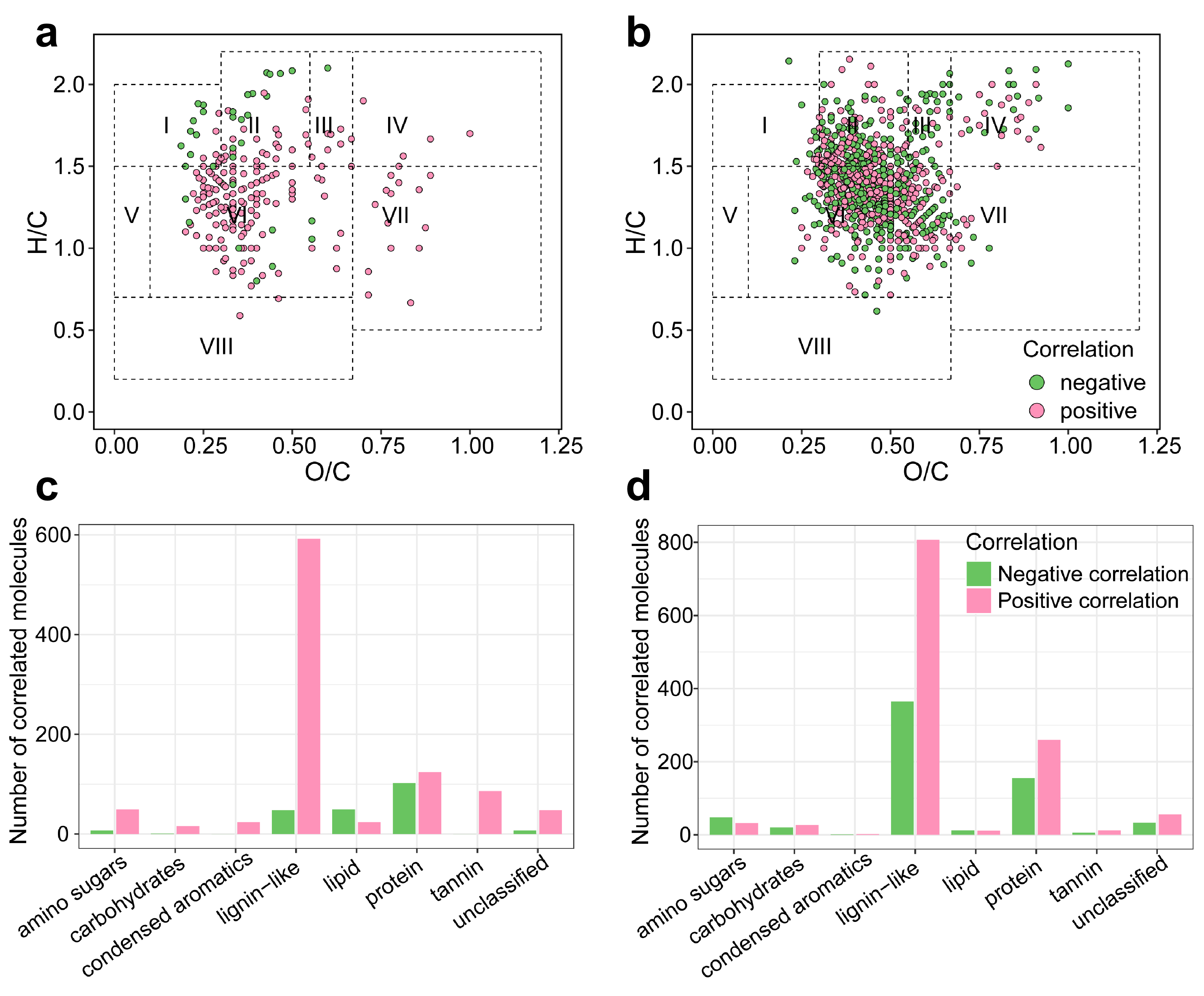
Supplementary Figure 12.** **Correlation patterns between DOM molecules and bacterial communities across low- and high-salinity conditions. a, b** The distribution of DOM molecular formulae that were significantly correlated with bacterial OTUs in the LS **(a)** and HS **(b)** groups. Points are colored according to the direction of correlation (positive or negative). **c, d** The number of positively and negatively correlated DOM molecules across different compound classes in the LS **(c)** and HS **(d)** groups.


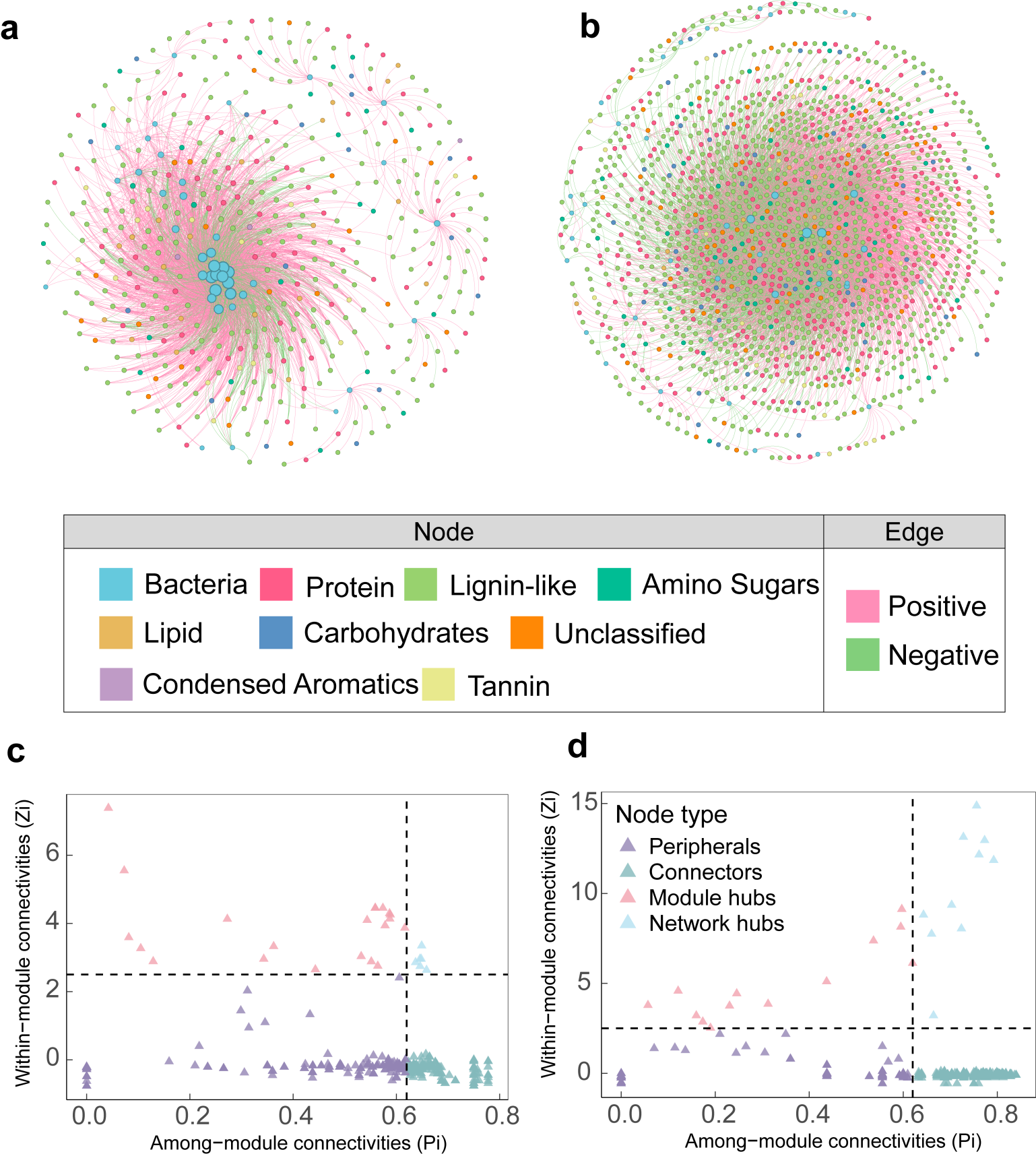


**Supplementary Figure 13.** Sensitivity analysis of the bacteria-DOM association network under a relaxed correlation threshold. **a, b** The low-salinity (LS) and high-salinity (HS) bacteria-DOM association networks reconstructed using a relaxed cutoff (|r| > 0.8, FDR-adjusted *P* < 0.05). Nodes represent bacterial OTUs and DOM molecules, colored by node category/chemical class, and edges represent positive or negative associations. Compared with the primary networks inferred at the more stringent threshold (|r| > 0.9, FDR-adjusted *P* < 0.05), the relaxed cutoff increased the absolute numbers of nodes and edges in both salinity groups, as expected from the inclusion of additional weaker associations. However, the major relative patterns remained qualitatively consistent: the HS network retained a larger association structure with more nodes and edges, a higher proportion of negative associations, and greater modularity, whereas the LS network remained denser and more positively connected (Table S11). These results indicate that the principal topological contrasts between salinity groups were robust to moderate variation in the correlation threshold. **c, d** The corresponding Zi-Pi plots for the LS and HS networks inferred at |r| > 0.8. Each point represents a node positioned by within-module connectivity (Zi) and among-module connectivity (Pi), and node roles were assigned using the standard Zi-Pi criteria for peripherals, connectors, module hubs, and network hubs. Under the relaxed threshold, the identity of the core hub nodes remained largely stable: in the LS network, six of the eight network hubs identified under the stringent threshold were recovered, and in the HS network, both original network hubs were retained, although additional hub candidates emerged under the relaxed cutoff (Table S12). Together, these results show that both the overall association topology and the identification of key hub nodes were robust to threshold choice, supporting the stability of the main biological interpretations derived from the bacteria-DOM association network.


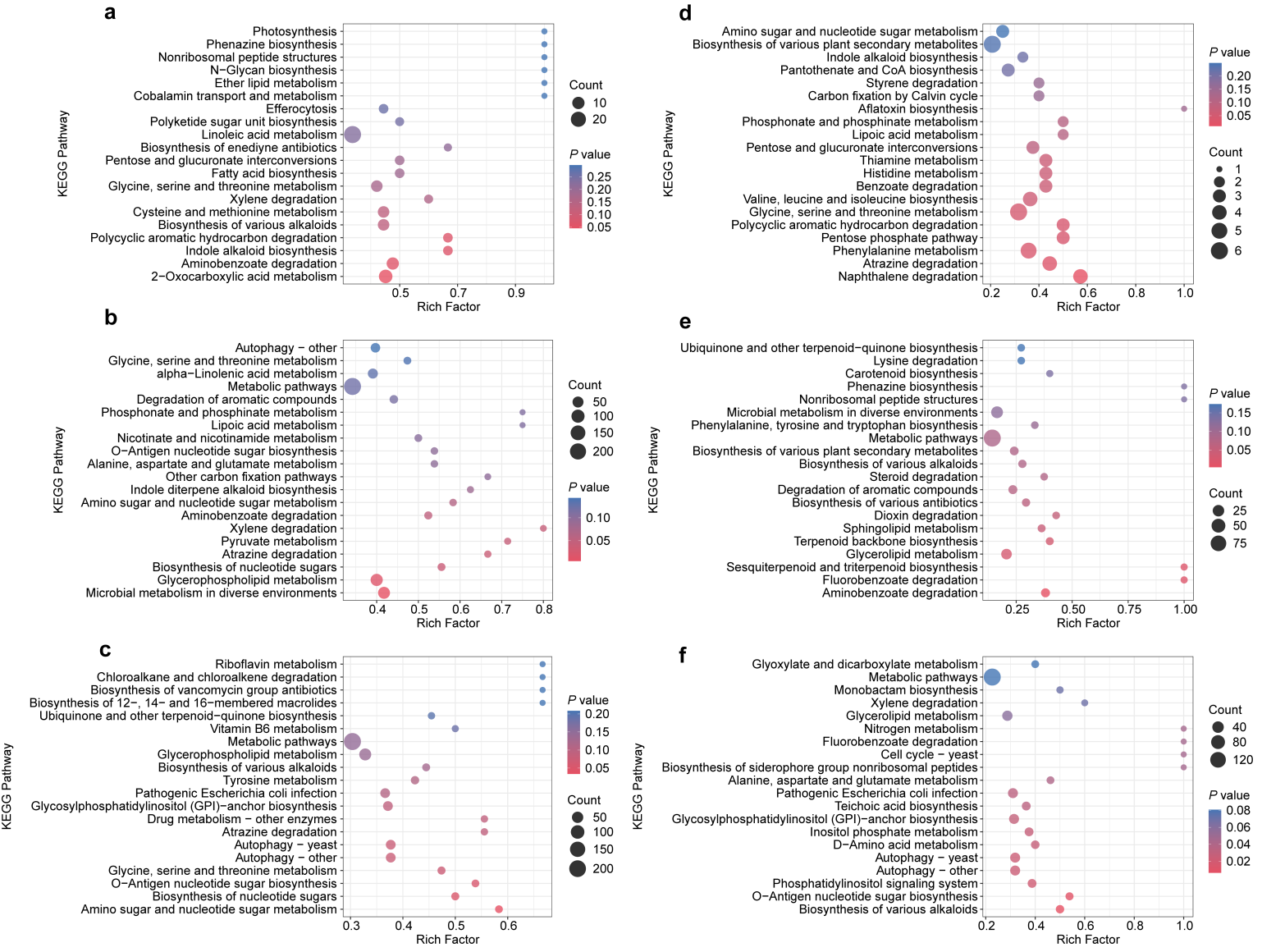
**Supplementary Figure 14. KEGG pathway enrichment analysis of significantly altered metabolites during 100-day incubation under low-salinity (LS) and high-salinity (HS) conditions. a-c** KEGG enrichment of metabolites that showed significant temporal changes between day 0 and day 100 in the low-salinity water **(a)**, water-sediment interface **(b)**, and sediment **(c)** environments. In LS samples, enriched pathways mainly included pentose and glucuronate interconversions, glycine-serine-threonine metabolism, and several vitamin-related biosynthetic routes (e.g., riboflavin, thiamine, and nicotinate metabolism). **d-f** KEGG enrichment of metabolites significantly altered between day 0 and day 100 in the high-salinity water **(d)**, water-sediment interface **(e)**, and sediment **(f)** environments. In HS samples, enriched pathways prominently involved ubiquinone and other terpenoid-quinone biosynthesis, amino-acid degradation, the pentose phosphate and glyoxylate cycles, as well as degradation pathways for aromatic and xenobiotic compounds such as benzoate, xylene, and polycyclic aromatic hydrocarbons. Each bubble represents a KEGG pathway, with bubble size indicating the number of significantly changed metabolites mapped to that pathway and bubble color representing the hypergeometric-test *P* value. The x-axis shows the Rich Factor (ratio of the number of significant metabolites mapped to a pathway to the total number of metabolites annotated to that pathway). Pathways are ranked by *P* value, and only the top enriched pathways are displayed.


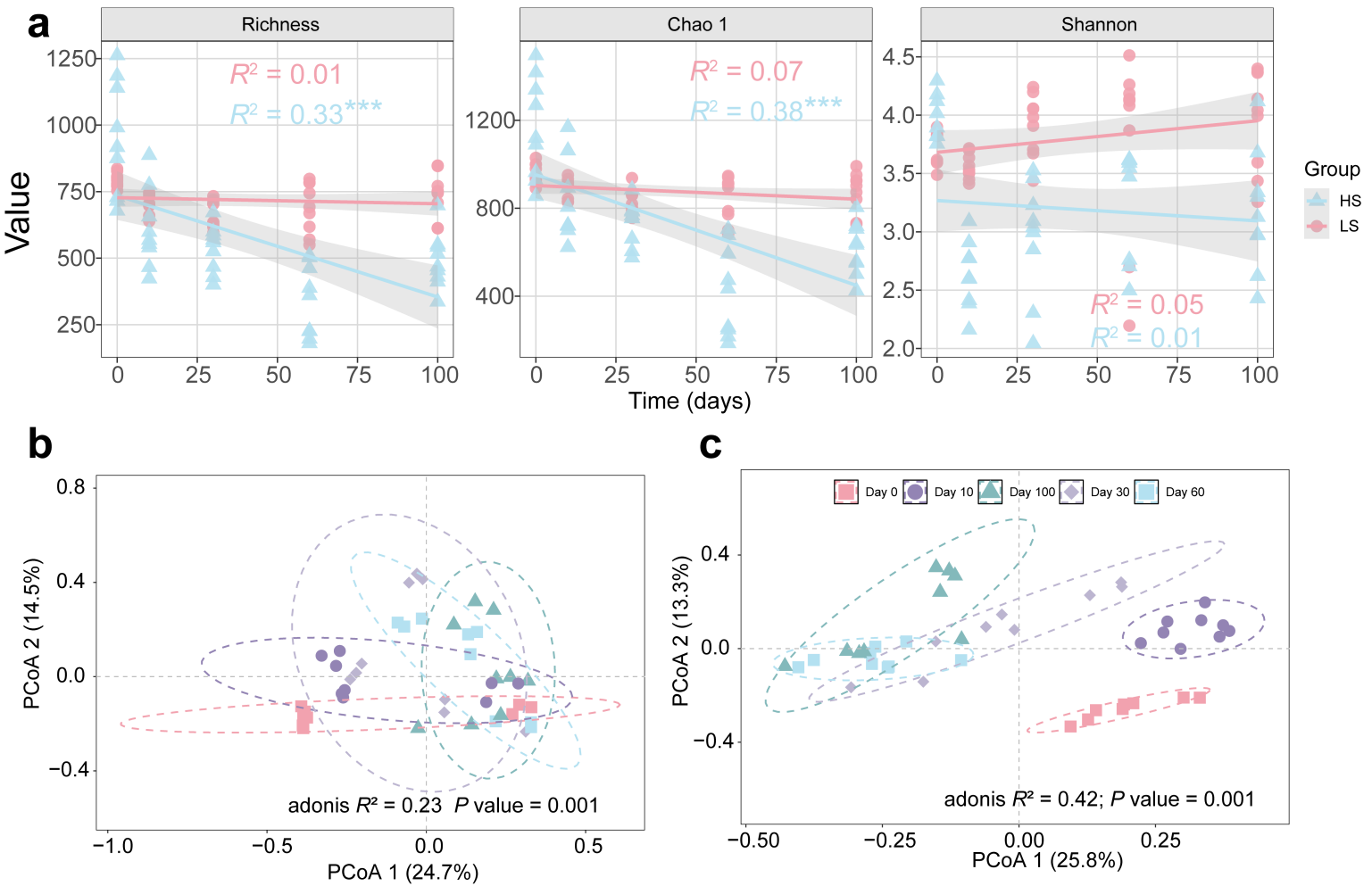


**Supplementary Figure 15.** **Temporal dynamics of bacterial diversity and community structure during the 100-day indoor incubation experiment. a** Linear regression of three α-diversity indices—Richness, Chao1, and Shannon—across incubation time (Day 0, 10, 30, 60, 100) under low-salinity (LS) and high-salinity (HS) conditions. Shaded of grey represent 95% confidence intervals. To reduce the likelihood of false positives, *P* values from all regression analyses were corrected using the Benjamini-Hochberg false discovery rate (FDR) procedure. b, c PCoA of bacterial community composition over the incubation period for LS (b) and HS (c) samples. Points are grouped by sampling day, and ellipses represent 95% confidence intervals around each time point. PERMANOVA (adonis) statistics (*R*^2^ and *P* value) summarize the proportion of community variation attributable to incubation time within each salinity level. ****P* < 0.001.

**
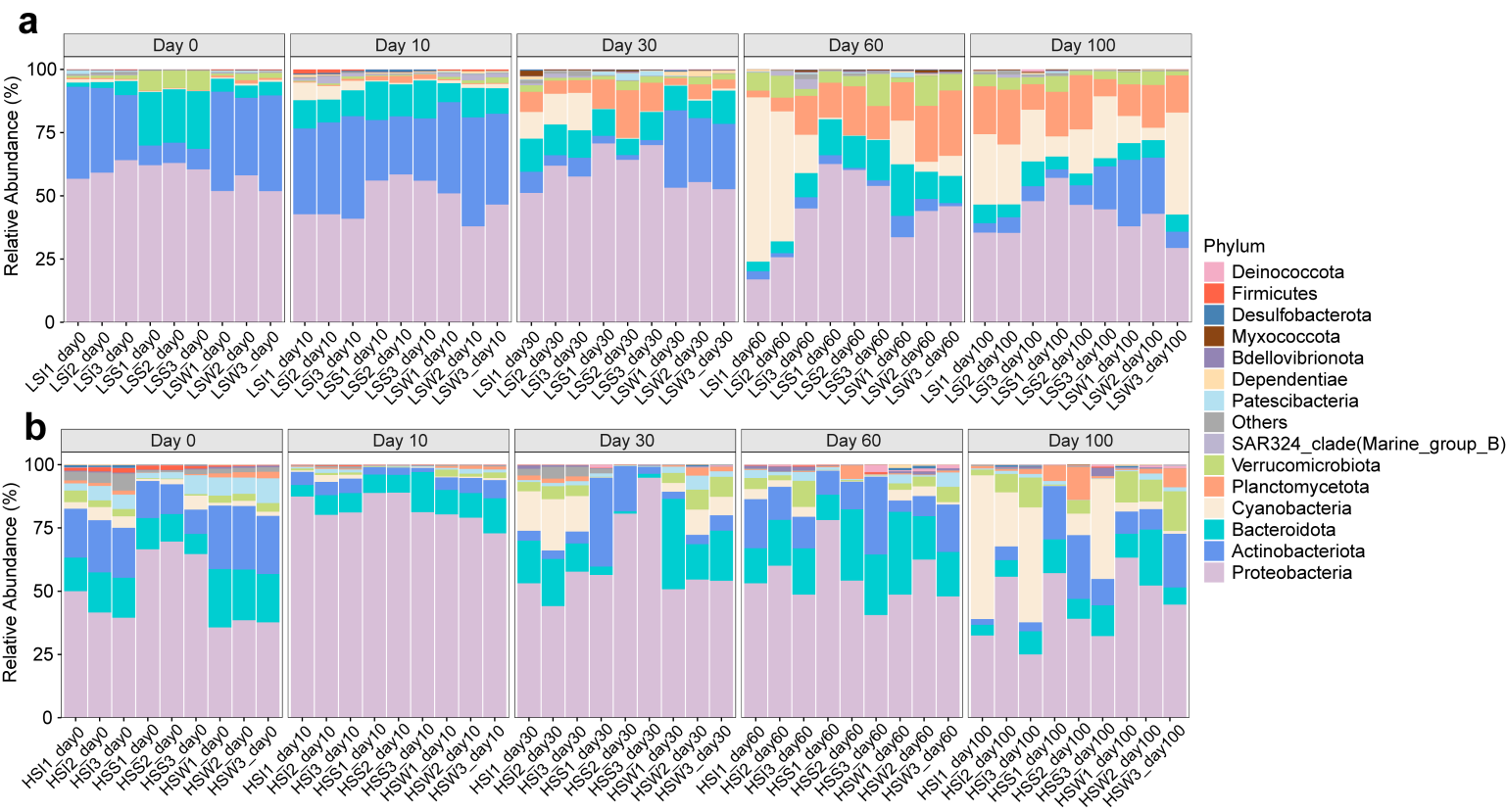
Supplementary Figure 16.** **Temporal shifts in the relative abundance of dominant bacterial phyla during 100-day indoor incubation. a, b** Relative abundance dynamics of the top 15 most abundant bacterial phyla across five incubation time points (day 0, 10, 30, 60, and 100) under low-salinity **(a)** and high-salinity **(b)** conditions. Phyla outside the top-30 ranking were grouped into “Others” Each stacked bar represents an individual sample, grouped by medium type (water, water-sediment interface, sediment) and time.

**
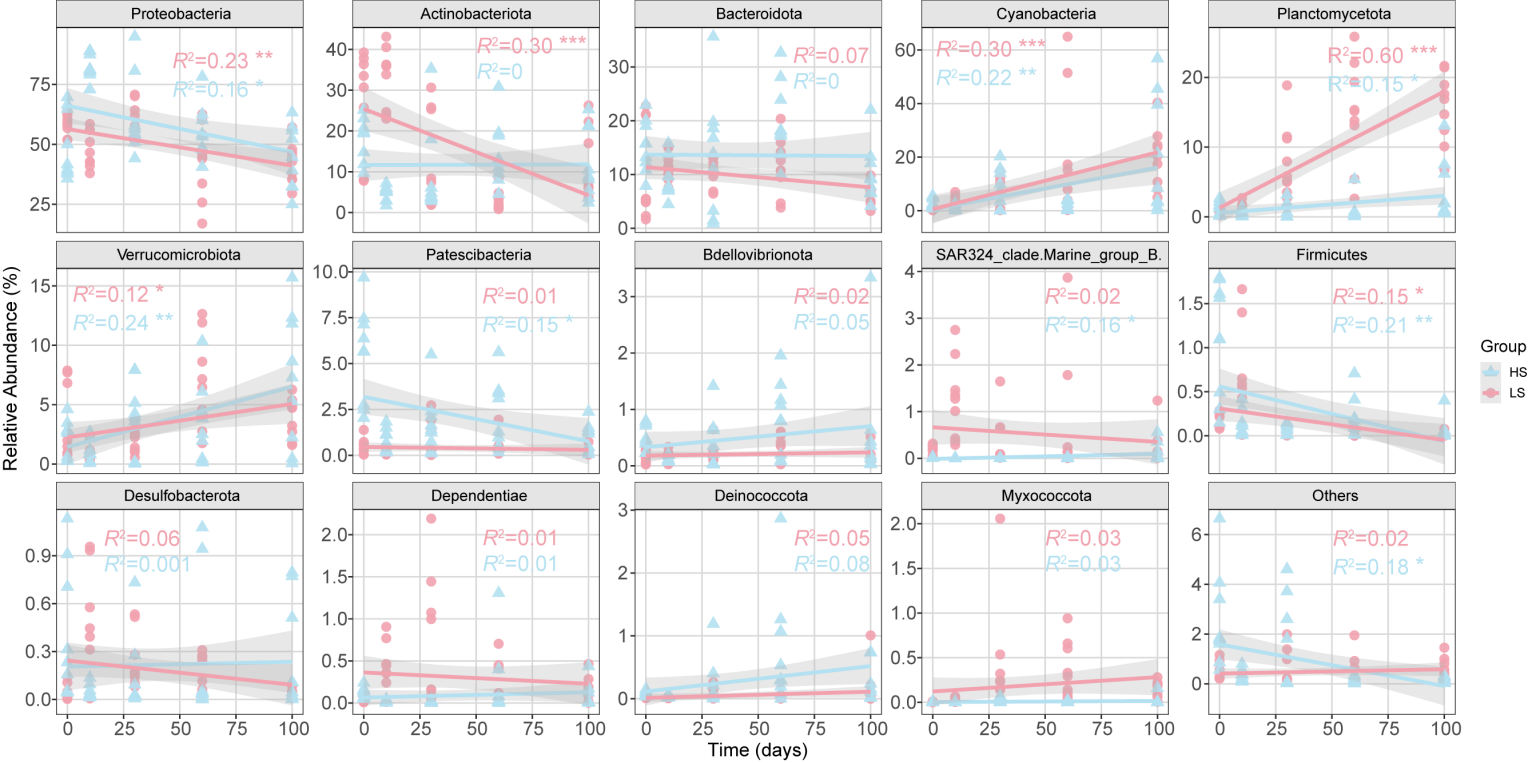
**

**Supplementary Figure 17. Linear regression analysis of the 15 most abundant bacterial phyla showing changes in relative abundance over time (Day 0, 10, 30, 60, and 100) in low-salinity (LS) and high-salinity (HS) incubations.** Shaded of grey represent 95% confidence intervals. To reduce the likelihood of false positives, *P* values from all regression analyses were corrected using the Benjamini-Hochberg false discovery rate (FDR) procedure. **P* < 0.05, ***P* < 0.01, and ****P* < 0.001

**
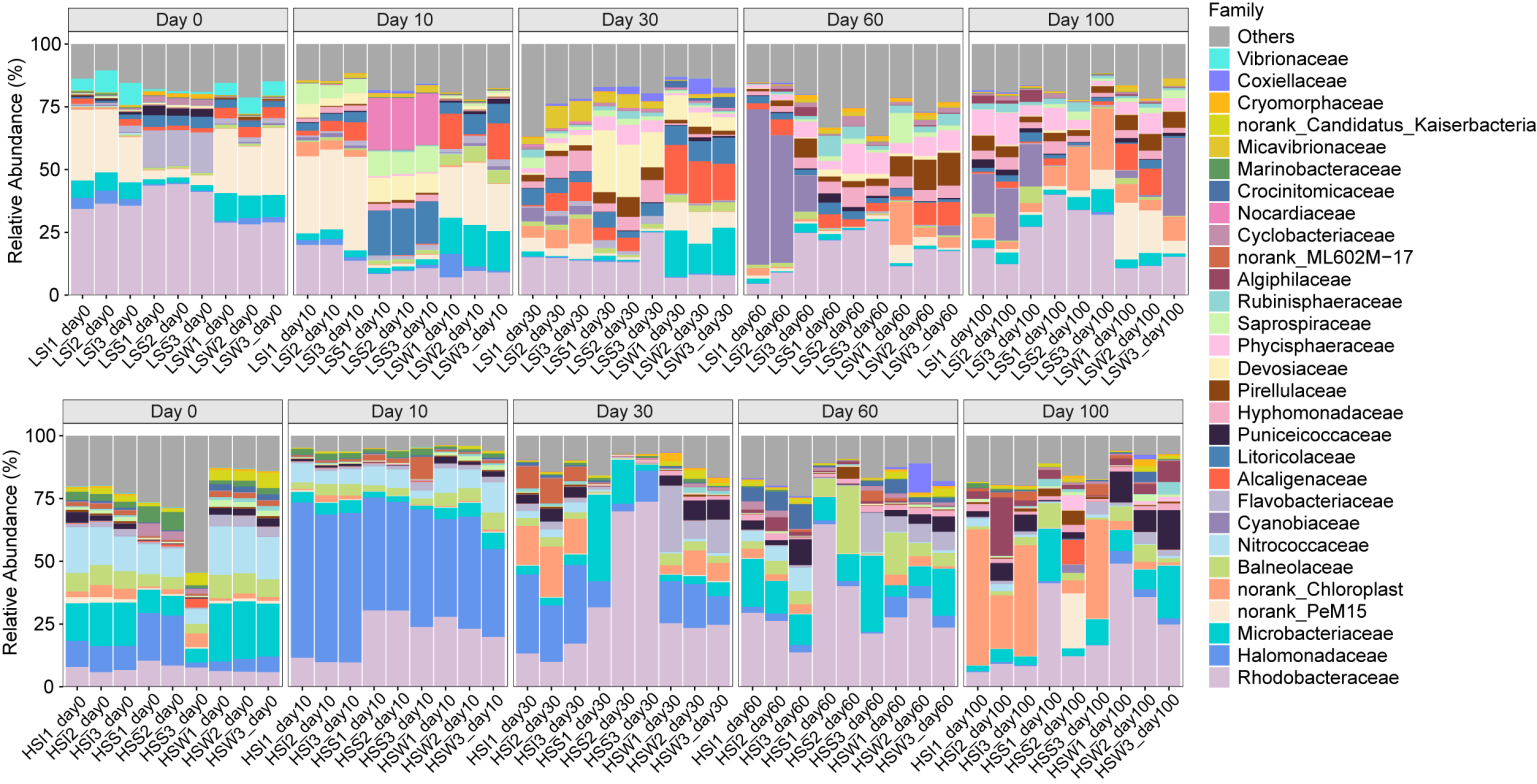
Supplementary Figure 18. Temporal shifts in the relative abundance of dominant bacterial families during 100-day incubation.** a, b Relative abundance patterns of the top 30 most abundant bacterial families across five incubation time points (day 0, 10, 30, 60, and 100) under low-salinity **(a)** and high-salinity **(b)** conditions. Families outside the top-30 ranking were grouped into “Others”. Each stacked bar represents an individual sample, arranged by medium type (water, water-sediment interface, and sediment) and sampling day

**
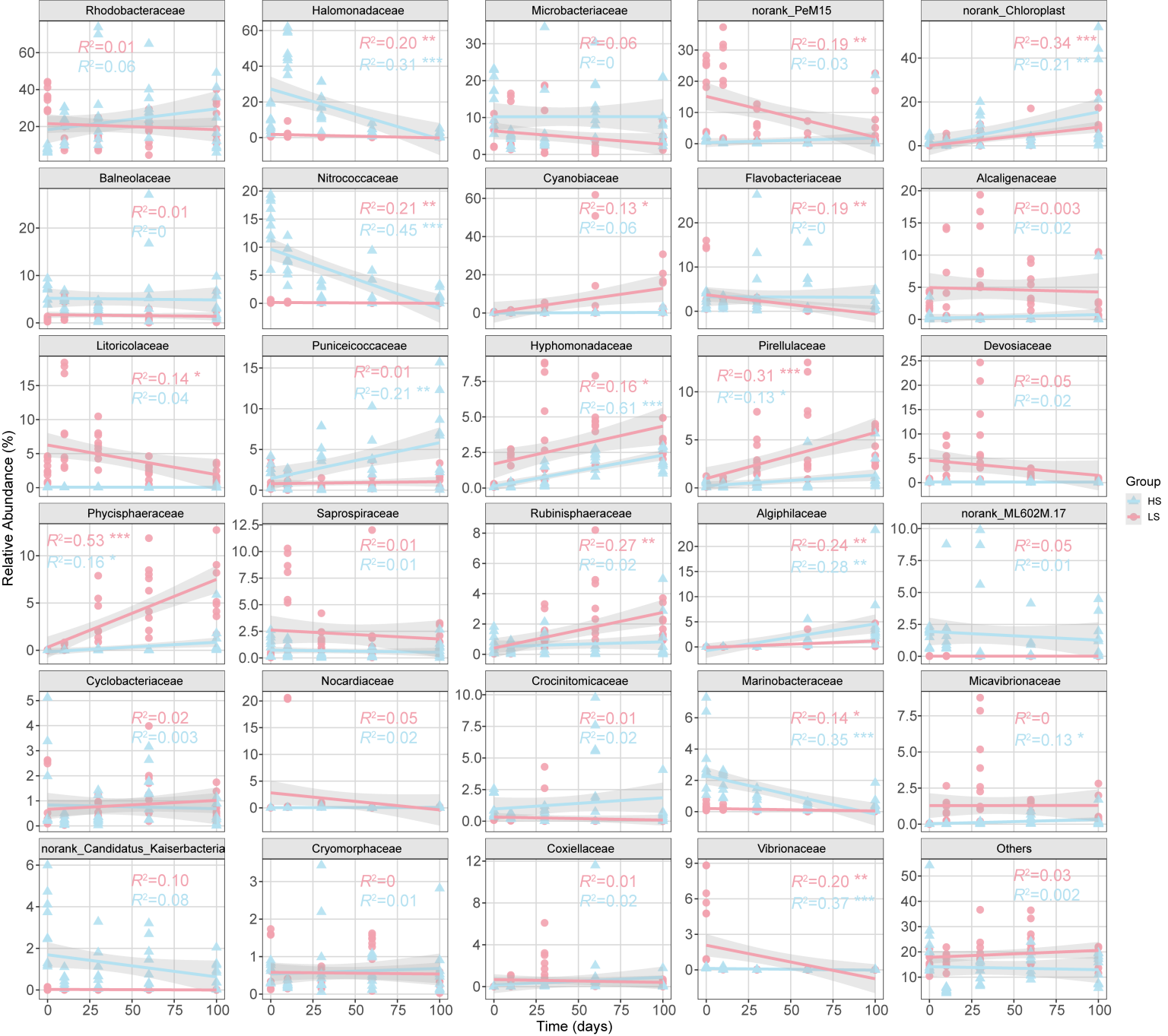
Supplementary Figure 19. Linear regression analysis of the 30 most abundant bacterial families showing temporal trends in relative abundance across five incubation time points (Day 0, 10, 30, 60, and 100) under low-salinity (LS) and high-salinity (HS) conditions**. Shaded grey areas represent 95% confidence intervals for regression fits. To minimize false-positive findings, *P* values from all regression models were adjusted using the Benjamini-Hochberg false discovery rate (FDR) correction. **P* < 0.05, ***P* < 0.01, ****P* < 0.001.

**Supplementary Table S1**. Replicate-specific nitrate and phosphate additions used in the incubation experiment

| Treatment | Initial DOC (mg C L^-^¹) | Added nitrate-N (mg N L^-^¹) | Added nitrate-N (μM) | Added phosphate-P (mg P L^-^¹) | Added phosphate-P (μM) | Equivalent NaNO_3_ added (mg L^-^¹) | Equivalent KH_2_PO_4_ added (mg L^-^¹) |
| --- | --- | --- | --- | --- | --- | --- | --- |
| HSW1 | 6.917 | 1.218 | 86.96 | 0.168 | 5.43 | 7.388 | 0.739 |
| HSW2 | 6.958 | 1.225 | 87.47 | 0.170 | 5.47 | 7.432 | 0.744 |
| HSW3 | 6.892 | 1.213 | 86.64 | 0.168 | 5.41 | 7.362 | 0.737 |
| HSI1 | 7.032 | 1.237 | 88.39 | 0.171 | 5.52 | 7.511 | 0.752 |
| HSI2 | 6.916 | 1.218 | 86.95 | 0.168 | 5.43 | 7.387 | 0.739 |
| HSI3 | 6.951 | 1.224 | 87.38 | 0.170 | 5.46 | 7.425 | 0.743 |
| HSS1 | 6.893 | 1.214 | 86.65 | 0.168 | 5.42 | 7.363 | 0.737 |
| HSS2 | 6.919 | 1.218 | 86.98 | 0.168 | 5.44 | 7.390 | 0.740 |
| HSS3 | 6.885 | 1.212 | 86.55 | 0.168 | 5.41 | 7.354 | 0.736 |
| LSW1 | 6.920 | 1.218 | 86.99 | 0.168 | 5.44 | 7.392 | 0.740 |
| LSW2 | 6.934 | 1.221 | 87.16 | 0.169 | 5.45 | 7.406 | 0.741 |
| LSW3 | 6.851 | 1.207 | 86.11 | 0.167 | 5.38 | 7.318 | 0.732 |
| LSI1 | 6.965 | 1.226 | 87.56 | 0.170 | 5.47 | 7.440 | 0.744 |
| LSI2 | 6.957 | 1.225 | 87.46 | 0.170 | 5.47 | 7.431 | 0.744 |
| LSI3 | 6.998 | 1.232 | 87.98 | 0.171 | 5.50 | 7.475 | 0.748 |
| LSS1 | 6.930 | 1.220 | 87.11 | 0.169 | 5.44 | 7.402 | 0.741 |
| LSS2 | 6.881 | 1.211 | 86.50 | 0.168 | 5.41 | 7.350 | 0.735 |
| LSS3 | 6.941 | 1.221 | 87.25 | 0.169 | 5.45 | 7.414 | 0.742 |

Replicate-specific additions of nitrate and phosphate used to establish nutrient-replete incubation conditions. Additions were calculated from the day-0 DOC concentration of each incubation microcosm using a target molar C:N:P ratio of 106:16:1. Nitrate was supplied as NaNO_3_ and phosphate as KH_2_PO_4_. Added concentrations are reported as nitrate-N and phosphate-P, together with the equivalent mass additions of the two salts.

**Supplementary Table S2.** Relative abundance of DOM molecular classes in low-salinity (LS) and high-salinity (HS) groups.

| DOM molecular class | LS | HS |
| --- | --- | --- |
| CHSO (%) | 21.7 ± 8.4 | 30.2 ± 3.1 |
| CHSNO (%) | 14.1 ± 10.1 | 19.9 ± 4.2 |
| CHO (%) | 34.1 ± 13.5 | 23.2 ± 2.7 |
| CHNO (%) | 30.0 ± 4.9 | 26.4 ± 4.5 |
| Amino Sugars (%) | 5.7 ± 0.7 | 6.1 ± 0.2 |
| Carbohydrates (%) | 4.5 ± 1.1 | 4.3 ± 0.4 |
| Condensed Aromatics (%) | 0.8 ± 0.6 | 0.5 ± 0.4 |
| Lignin-like (%) | 52.3 ± 2.8 | 51.8 ± 2.8 |
| Lipid (%) | 5.6 ± 2.8 | 3.7 ± 2.3 |
| Protein (%) | 22.3 ± 2.0 | 25.0 ± 0.7 |
| Tannin (%) | 3.1 ± 1.6 | 3.3 ± 0.7 |
| Unclassified (%) | 5.7 ± 1.0 | 5.3 ± 0.4 |
| CRAM (%) | 37.7 ± 4.6 | 35.3 ± 1.4 |

Values are presented as mean ± standard deviation (n = 3).

**Supplementary Table S3.** Elemental and molecular indices of DOM in low-salinity (LS) and high-salinity (HS) groups.

| Chemical parameters | LS | HS |
| --- | --- | --- |
| AI_mod_ | 0.10 ± 0.30 | 0.08 ± 0.27 |
| DBE | 6.95 ± 3.03 | 6.93 ± 3.08 |
| NOSC | -0.38 ± 0.46 | -0.39 ± 0.43 |
| H/C | 1.41 ± 0.29 | 1.44 ± 0.28 |
| O/C | 0.47 ± 0.15 | 0.47 ± 0.14 |

Values represent the mean ± standard deviation of five molecular-level indices, including modified aromaticity index (AI_mod_), double bond equivalents (DBE), nominal oxidation state of carbon (NOSC), hydrogen-to-carbon ratio (H/C), and oxygen-to-carbon ratio (O/C).

**Supplementary Table S4.** The spectral properties of three fluorescent components were examined using PARAFAC with previously identified sources.

| Component | Ex/Em and peak (LS) | Ex/Em and peak (HS) | Description | Probable source | Reference |
| --- | --- | --- | --- | --- | --- |
| C1 | 245/390 nm: peak A  315/390 nm: peak M | 310/385 nm: peak M | Humic-like | Terrestrial and marine | [8]  [9] |
| C2 | 275/445 nm : peak C  365/445 nm : peak C | 275/445 nm: peak C  360/445 nm: peak C | Humic-like | Terrestrial | [10]  [8] |
| C3 | 230/335 nm: peak T  280/335 nm: peak T | 235/335 nm: peak T  285/335 nm: peak T | Tryptophan-like | Autochthonous | [10]  [11] |

**Supplementary Table S5.** Environmental parameters of low-salinity water (LSW) and high-salinity water (HSW) samples.

| Environmental parameters | LSW | HSW |
| --- | --- | --- |
| pH | 7.63 ± 0.99^a^ | 7.75 ± 0.58^a^ |
| SAL (ppt) | 28.7 ± 1.72^a^ | 88.02 ± 6^b^ |
| SPC (us/cm) | 44.4 ± 2.38^a^ | 121.04 ± 5.71^b^ |
| TP (mg/L) | 0.28 ± 0.02^a^ | 0.82 ± 0.3^b^ |
| DOC (mg/L) | 5.81 ± 0.75^a^ | 8.21 ± 1.03^b^ |
| TDS (g/L) | 29.06 ± 1.83^a^ | 78.48 ± 3.8^b^ |
| NH_4_^+^-N (mg/L) | 1.26 ± 0.16^a^ | 1.81 ± 0.67^a^ |
| TN (mg/L) | 7.11 ± 8.25^a^ | 1.86 ± 2.06^a^ |
| COD (mg/L) | 5.86 ± 0.32^a^ | 5.56 ± 0.34^a^ |

Values are presented as mean ± standard deviation (n = 5). Different lowercase letters (a, b) within the same row indicate significant differences (*P* < 0.05, t-test).Abbreviations: SAL, salinity; SPC, specific conductance; TP, total phosphorus; DOC, dissolved organic carbon; TDS, total dissolved solids; NH₄^+^-N, ammonium nitrogen; TN, total nitrogen; COD, chemical oxygen demand.

**Supplementary Table S6.** Environmental parameters of low-salinity interface (LSI) and high-salinity interface (HSI) samples.

| Environmental parameters | LSI | HSI |
| --- | --- | --- |
| pH | 7.06 ± 0.89^a^ | 8.09 ± 0.82^a^ |
| SAL (ppt) | 28.19 ± 2.03^a^ | 90.07 ± 12.08^b^ |
| SPC (us/cm) | 41.69 ± 3.72^a^ | 124.95 ± 9.66^b^ |
| TP (mg/L) | 0.28 ± 0.03^a^ | 0.85 ± 0.29^b^ |
| DOC (mg/L) | 5.7 ± 0.88^a^ | 8.62 ± 0.63^b^ |
| TDS (g/L) | 29.05 ± 2.11^a^ | 81.45 ± 7.99^b^ |
| NH_4_^+^-N (mg/L) | 1.32 ± 0.1^a^ | 1.7 ± 0.58^a^ |
| TN (mg/L) | 7.77 ± 9.14^a^ | 1.71 ± 1.76^a^ |
| COD (mg/L) | 5.59 ± 0.66^a^ | 5.91 ± 0.88^a^ |

Values are presented as mean ± standard deviation (n = 5). Different lowercase letters (a, b) within the same row indicate significant differences (*P* < 0.05, t-test).Abbreviations: SAL, salinity; SPC, specific conductance; TP, total phosphorus; DOC, dissolved organic carbon; TDS, total dissolved solids; NH_4_^+^-N, ammonium nitrogen; TN, total nitrogen; COD, chemical oxygen demand.

**Supplementary Table S7**. Environmental parameters of low-salinity sediment (LSS) and high-salinity sediment (HSS) samples.

| Environmental parameters | LSS | HSS |
| --- | --- | --- |
| pH | 8.67 ± 0.16^a^ | 8.09 ± 0.82^a^ |
| SPC (us/cm) | 54.34 ± 19.19^a^ | 197.53 ± 52.77^b^ |
| TOC (%) | 0.5 ± 0.2^a^ | 0.7 ± 0.4^a^ |
| TSi (mg/g) | 222.23 ± 32.16^a^ | 190.34 ± 19.98^a^ |
| TP (mg/g) | 0.54 ± 0.04^a^ | 0.85 ± 0.07^b^ |
| TFe (mg/g) | 22.45 ± 2.46^a^ | 17.8 ± 5.69^a^ |
| TAl (mg/g) | 66.43 ± 8.67^a^ | 50.44 ± 7.4^b^ |
| DOC (mg/L) | 4.92 ± 0.77^a^ | 6.55 ± 0.46^b^ |
| SAL (ppt) | 24.78± 4.76^a^ | 95.87 ± 12.13^b^ |

Values are presented as mean ± standard deviation (n = 5). Different lowercase letters (a, b) within the same row indicate significant differences (*P* < 0.05, t-test). Abbreviations: SPC, specific conductance; TOC, total organic carbon; TSi, total silicon; TP, total phosphorus; TFe, total iron; TAl, total aluminum; DOC, dissolved organic carbon; SAL, salinity.

**Supplementary Table S8.** Topological properties of bacteria-DOM co-occurrence networks in low-salinity (LS) and high-salinity (HS) groups.

| Topology parameters | LS | HS |
| --- | --- | --- |
| Node | 263 | 916 |
| Edge | 1177 | 1847 |
| Positive connections (%) | 81.8 | 65.4 |
| Negative connections (%) | 18.2 | 34.7 |
| Network diameter | 7.36 | 8.23 |
| Average path length | 2.44 | 2.59 |
| Density | 0.03 | 0.004 |

**Supplementary Table S9.** Comparison of node centrality metrics between bacterial and DOM nodes in the co-occurrence network.

| Centrality parameters | Bacteria | DOM |
| --- | --- | --- |
| Degree centrality | 45.82 ± 93.66^a^ | 2.72 ± 2.29^b^ |
| Betweenness centrality | 8988.44 ± 30286.40^a^ | 172.11 ± 1518.54^b^ |
| Closeness centrality | 0.18 ± 0.37^a^ | 0.01 ± 0.10^b^ |
| Eigen centrality | 0.20 ± 0.31^a^ | 0.06 ± 0.06^b^ |

Values are presented as mean ± standard deviation Different superscript letters indicate significant differences between bacteria and DOM (*P* < 0.05, Mann-Whitney U test).

**Supplementary Table S10.** Taxonomic annotation of network hubs identified in low-salinity (LS) and high-salinity (HS) co-occurrence networks

| Group | OTU ID |  | Phylum | Class | Order | Family |
| --- | --- | --- | --- | --- | --- | --- |
| LS | OTU66 | Network hubs | Firmicutes | Bacilli | Izemoplasmatales | norank_Izemoplasmatales |
|  | OTU26 |  | Proteobacteria | Alphaproteobacteria | Rhodobacterales | Rhodobacteraceae |
|  | OTU5002 |  | Proteobacteria | Alphaproteobacteria | Rhodobacterales | Rhodobacteraceae |
|  | OTU38 |  | Actinobacteriota | Actinobacteria | PeM15 | norank_PeM15 |
|  | OTU7 |  | Actinobacteriota | Actinobacteria | PeM15 | norank_PeM15 |
|  | OTU73 |  | Bacteroidota | Bacteroidia | Flavobacteriales | Flavobacteriaceae |
|  | OTU845 |  | Actinobacteriota | Actinobacteria | Micrococcales | Microbacteriaceae |
|  | OTU941 |  | Actinobacteriota | Actinobacteria | Micrococcales | Microbacteriaceae |
|  | OTU160 |  | Bacteroidota | Bacteroidia | Flavobacteriales | Cryomorphaceae |
|  | OTU143 |  | Actinobacteriota | Acidimicrobiia | Microtrichales | Ilumatobacteraceae |
| HS | OTU137 | Network hubs | Verrucomicrobiota | Verrucomicrobiae | Opitutales | Puniceicoccaceae |
|  | OTU46 |  | Proteobacteria | Gammaproteobacteria | norank_Gammaproteobacteria | norank_Gammaproteobacteria |

The table lists all hub OTUs detected in the LS (n = 8) and HS (n = 2) networks, along with their corresponding taxonomic assignments at the phylum, class, order, and family levels.

**Supplementary Table S11.** Sensitivity analysis of bacteria-DOM association network topology under two correlation thresholds.

| Topology parameters | LS-0.9 | LS-0.8 | HS-0.9 | HS-0.8 | Qualitative consistency |
| --- | --- | --- | --- | --- | --- |
| Nodes | 263 | 460 | 916 | 1600 | HS > LS at both thresholds |
| Edges | 1177 | 3445 | 1847 | 7648 | HS > LS at both thresholds |
| Positive edges (%) | 81.8 | 83.3 | 65.3 | 58.8 | LS > HS at both thresholds |
| Negative edges (%) | 18.2 | 16.7 | 34.7 | 41.2 | HS > LS at both thresholds |
| Average degree | 8.951 | 14.978 | 4.033 | 9.560 | LS > HS at both thresholds |
| Network density | 0.0342 | 0.0326 | 0.0044 | 0.0060 | LS > HS at both thresholds |
| Network diameter | 7.315 | 7.082 | 8.226 | 11.240 | HS > LS at both thresholds |
| Modularity | 0.231 | 0.203 | 0.378 | 0.218 | HS > LS at both thresholds |

Sensitivity analysis of bacteria-DOM association network topology under alternative correlation thresholds. Here, LS-0.9 and HS-0.9 denote the low-salinity (LS) and high-salinity (HS) bacteria-DOM association networks constructed using the primary stringent threshold (|r| > 0.9, FDR-adjusted *P* < 0.05), whereas LS-0.8 and HS-0.8 denote the corresponding sensitivity-analysis networks reconstructed using the relaxed threshold (|r| > 0.8, FDR-adjusted *P* < 0.05). Reported topological parameters include node number, edge number, proportions of positive and negative associations, average degree, network density, network diameter, and modularity. As expected, relaxing the correlation threshold increased the absolute numbers of nodes and edges in both salinity groups because additional weaker associations were retained. However, the major relative patterns between LS and HS remained qualitatively consistent across thresholds. In both comparisons, the HS networks consistently exhibited more nodes and edges, a higher proportion of negative associations, a larger network diameter, and higher modularity, whereas the LS networks consistently showed a higher proportion of positive associations, greater average degree, and higher network density. These results indicate that, although absolute topological values were sensitive to threshold choice, the principal biological interpretations derived from the bacteria-DOM network comparison remained stable. In particular, the HS networks retained a larger but more negatively connected and modular association structure, whereas the LS networks retained a denser and more positively connected association pattern. Thus, the main conclusions of the network analysis were robust to moderate variation in the correlation cutoff.

**Supplementary Table S12.** Zi-Pi network hub stability across correlation thresholds

| Group | Threshold | No. of network hubs | Identified network hubs | No. of overlapping hubs  between \|r \|> 0.9 and \|r\| > 0.8 | Overlapping hubs | Recovery of \|r\| > 0.9 hubs | Overlap within \|r\| > 0.8 hubs |
| --- | --- | --- | --- | --- | --- | --- | --- |
| LS | \|r\| > 0.9 | 8 | OTU66, OTU26, OTU5002, OTU38, OTU7, OTU73, OTU845, OTU941 | 6 | OTU26, OTU38, OTU66, OTU845, OTU941, OTU5002 | 75.0% | 100.0% |
| LS | \|r\| > 0.8 | 6 | OTU66, OTU26, OTU5002, OTU38, OTU845, OTU941 | 6 | OTU26, OTU38, OTU66, OTU845, OTU941, OTU5002 | 75.0% | 100.0% |
| HS | \|r\| > 0.9 | 2 | OTU137, OTU46 | 2 | OTU46, OTU137 | 100.0% | 18.2% |
| HS | \|r\| > 0.8 | 11 | OTU137, OTU161, OTU222, OTU231, OTU24, OTU3, OTU40, OTU42, OTU46, OTU49, OTU51 | 2 | OTU46, OTU137 | 100.0% | \|r\| > 0.8 |

Network hubs were identified based on within-module connectivity (Zi) and among-module co|r| > 0.9 and |r| > 0.8, both with FDR-adjusted *P* < 0.05) for low-salinity (LS) and high-salinity (HS) groups. For each group and threshold, the table reports the total number of network hubs, their identities, and the extent of overlap between hubs identified under the two thresholds. Overlap metrics include the number and identity of shared hubs, the recovery of hubs originally identified under the stricter threshold (|r| > 0.9) within the relaxed network, and the proportion of hubs under the relaxed threshold that overlap with the stricter-threshold set. In the LS group, six of the eight hubs identified under the stringent threshold were retained under the relaxed threshold (75% recovery), and all hubs detected under the relaxed threshold corresponded to those identified under the stringent threshold (100% overlap within |r| > 0.8), indicating high stability of hub identity with minimal introduction of new hub candidates. In the HS group, all hubs identified under the stringent threshold were retained under the relaxed threshold (100% recovery), but the relaxed network identified additional hub candidates, resulting in a lower proportion of overlap within the |r| > 0.8 set. This pattern suggests that, while the relaxed threshold increases the number of candidate hubs—particularly in the HS network—the core hub nodes identified under the stringent threshold are consistently preserved across thresholds. Overall, these results demonstrate that the identification of key network hubs is robust to moderate variation in correlation cutoff, and that the central nodes supporting the main biological interpretations are not dependent on a single threshold choice.

**Supplementary Table S13. Functional annotation of the 20 key metabolites identified in this study**

| Compounds | HMDB | KEGG | Functional Class | Notes |
| --- | --- | --- | --- | --- |
| 4-Nitrophenol | HMDB0001232 | C00870 | Nitrophenol compound | Phenolic metabolite involved in 4-aminobenzoate degradation and xenobiotic metabolism |
| Dopamine | HMDB0000073 | C03758 | Biogenic amine neurotransmitter | Catecholamine neurotransmitter derived from tyrosine; dopaminergic signalling |
| 2-Phenylacetamide | HMDB0010715 | C02505 | Aromatic amide | Aromatic amide intermediate in phenylalanine metabolism and styrene degradation |
| Taurocyamine | HMDB0003584 | C01959 | Guanidino-sulfonic acid derivative | Energy‑storage guanidino-sulfonic acid; taurine metabolism intermediate |
| N-Acetylleucine | HMDB0011756 | C02710 | Acetylated amino-acid derivative/anti-vertigo drug | Acetylated leucine derivative approved as anti‑vertigo drug |
| O-benzyl-L-serine | NA | NA | Benzylated non-natural amino acid | BenSer; inhibitor of ASCT2/LAT1 amino-acid transporters[12] |
| Tropine | HMDB0259297 | C00729 | Tropane alkaloid | Tropane alkaloid precursor for atropine/scopolamine biosynthesis |
| Arecaidine | HMDB0030352 | C10128 | Areca pyridine alkaloid | Betel-nut pyridine alkaloid; GABA re-uptake inhibitor |
| Primidone | HMDB0014932 | C07371 | Barbiturate antiepileptic | Barbiturate antiepileptic; pro-drug of phenobarbital |
| Pyridostigmine | HMDB0014685 | C07410 | Reversible acetylcholinesterase inhibitor | Reversible acetylcholinesterase inhibitor for myasthenia gravis |
| Zoxazolamine | HMDB0260050 | C13841 | Oxazolone derivative/central muscle relaxant | Central muscle relaxant withdrawn for hepatotoxicity |
| 6-Methyl-5-hepten-2-one | HMDB0035915 | C07287 | Volatile aliphatic ketone | Sulcatone; volatile ketone, mosquito attractant/alarm pheromone |
| Dimethirimol | NA | NA | Pyrimidine fungicide | Systemic pyrimidine fungicide; adenosine deaminase inhibitor https://sitem.herts.ac.uk/aeru/ppdb/en/Reports/232.htm |
| 2-Aminopyrazine | NA | NA | Heterocyclic amine (synthetic intermediate) | Building block in favipiravir synthesis https://www.scientificupdate.com/process-chemistry-articles/favipiravir-and-the-battle-against-covid-19/ |
| Sodium malate | NA | NA | Hydroxy dicarboxylate (TCA intermediate) | Malate salt; TCA-cycle intermediate https://rest.kegg.jp/get/C00149 |
| 4-Oxo-2-nonenal | HMDB0060285 | NA | Lipid peroxidation product/reactive aldehyde | Reactive lipid aldehyde causing protein cross-links[13]  https://hmdb.ca/metabolites/HMDB0060285 |
| 4alpha-formyl-4-methylzymosterol | HMDB0012167 | NA | Sterol biosynthesis intermediate | Intermediate in Bloch cholesterol biosynthesis https://hmdb.ca/metabolites/HMDB0012167 |
| Butylphthalide | NA | NA | Phthalide derivative/neuroprotectant | Phthalide neuroprotectant for acute ischemic stroke[14] |
| Isoproterenol hydrochloride | HMDB0015197 | C07056 | Non‑selective β‑adrenergic agonist | Non-selective β-adrenergic agonist; cardiac stimulant |
| Selegiline | HMDB0015171 | C07245 | Selective MAO‑B inhibitor | Selective MAO-B inhibitor for Parkinson’s disease |

The structural identifiers, pathway assignments and functional roles of the twenty metabolites that were selected for downstream interpretation of the metabolomic data set. “HMDB” and “KEGG” give the primary identifiers in the Human Metabolome Database and in the KEGG COMPOUND library, respectively. When a metabolite has no entry in KEGG, the field is marked “NA”. “Functional Class” summarises the major biochemical or pharmacological role inferred from KEGG BRITE categories or the cited literature. “Notes” supply concise functional descriptions; for compounds without KEGG records these notes also provide the literature or database source used to assign the class. Pathway information and BRITE classifications were obtained from the KEGG database (https://www.genome.jp/kegg/) and its integrated viewer ([https://www.genome.jp/dbget/](https://www.genome.jp/dbget/)) except where indicated otherwise. For metabolites lacking KEGG entries (NA) the functional assignment is based on (i) BRITE higher-level categories available for structural analogues or (ii) primary literature reports, the hyperlinks to which are included directly in the “Notes” column. All other hyperlinks in “Notes” point to peer-reviewed articles or specialised chemical resources and are formatted as underlined blue text for ease of access. Abbreviations: MAO-B, monoamine-oxidase B; ONE, 4-oxo-2-nonenal; TCA, tricarboxylic acid cycle. Beyond technical annotation, the metabolites compiled in this table also reveal common biochemical themes that characterize the high-salinity metabolome. Most compounds fall into a few major functional classes, including phenolic and aromatic amide intermediates (e.g. 4-nitrophenol, 2-phenylacetamide, primidone), biogenic amines and neurotransmitter-related molecules (e.g. dopamine, selegiline, isoproterenol hydrochloride), amino acid and amino-sulfonic acid derivatives (e.g. N-acetylleucine, taurocyamine), heterocyclic amines (e.g. 2-aminopyrazine), and pyridine or tropane alkaloids (e.g. arecaidine, tropine). Several metabolites are lipid peroxidation products or reactive carbonyls (e.g. 4-Oxo-2-nonenal, 6-methyl-5-hepten-2-one), indicating active oxidative processing of membrane lipids under hypersaline conditions. Functionally, many of these metabolites are linked to pathways for aromatic-ring and xenobiotic degradation (e.g. 4-nitrophenol in 4-aminobenzoate/xenobiotic metabolism, benzoate- and styrene-related intermediates), amine and amino-acid turnover (e.g. dopamine, N-acetylleucine, taurocyamine), or redox and stress responses (e.g. MAO-B inhibitors, catecholamine analogues, reactive aldehydes). Together with the random-forest results, this pattern suggests that microbes in the high-salinity treatments relied strongly on the biotransformation of aromatic, amine-rich and lipid-derived substrates, coupling xenobiotic and aromatic-compound metabolism, nitrogen-containing heterocycle processing, and oxidative lipid turnover to the observed restructuring of DOM molecular composition.

**Supplementary Table S14. Parameter estimates, fit statistics, and convergence diagnostics for the two-pool DOC decay model across treatments**

| Condition | n | BDOC (95% CI) | k (95% CI) | RDOC (95% CI) | BDOC (%) | RDOC (%) | *R*^2^ | RMSE | Starts tested/successful/failed | First start converged / Final fit at boundary |
| --- | --- | --- | --- | --- | --- | --- | --- | --- | --- | --- |
| LSW | 3 | 1.612 (1.461-1.762) | 0.085 (0.080-0.090) | 5.326 (5.237-5.415) | 23.2 | 76.8 | 0.981 | 0.074 | 54/54/0 | 3/0 |
| LSI | 3 | 1.767 (1.539-1.995) | 0.084 (0.056-0.112) | 5.173 (4.987-5.360) | 25.4 | 74.6 | 0.991 | 0.050 | 54/54/0 | 3/0 |
| LSS | 3 | 1.576 (1.435-1.718) | 0.080 (0.075-0.086) | 5.389 (5.275-5.503) | 22.6 | 77.4 | 0.989 | 0.054 | 54/54/0 | 3/0 |
| HSW | 3 | 3.823 (3.412-4.233) | 0.089 (0.068-0.109) | 3.875 (3.739-4.011) | 49.6 | 50.4 | 0.995 | 0.083 | 54/54/0 | 3/0 |
| HSI | 3 | 3.591 (3.248-3.934) | 0.079 (0.062-0.096) | 3.927 (3.763-4.091) | 47.7 | 52.3 | 0.991 | 0.116 | 54/54/0 | 3/0 |
| HSS | 3 | 3.707 (3.646-3.769) | 0.080 (0.069-0.092) | 3.962 (3.881-4.043) | 48.3 | 51.7 | 0.977 | 0.189 | 54/54/0 | 3/0 |

Parameter estimates, fit statistics, and convergence diagnostics for the two-pool exponential model describing DOC decay under different salinity treatments. BDOC represents the biodegradable DOC pool, RDOC represents the recalcitrant DOC, and k is the apparent first-order decay constant of the labile pool. Values are shown as treatment means based on replicate-level fits (n = 3), with 95% confidence intervals calculated from replicate-level parameter estimates. BDOC (%) and RDOC (%) indicate the relative contributions of the two pools to total modeled DOC. Model performance is summarized by the coefficient of determination (*R*^2^) and the root mean square error (RMSE). “Starts tested/successful/failed” indicates the number of initial-value combinations evaluated for each replicate during multi-start fitting. “First start converged/Final fit at boundary” indicates, respectively, the number of replicates for which the first starting-value combination converged and the number of replicates in which the final best-fit solution occurred at an imposed lower bound. All 54 starting-value combinations converged successfully for every replicate, and no final best-fit parameter estimate occurred at the imposed lower bounds.

**Supplementary Table S15.** Chemical characteristics of DOM at day 0 and day 100 under low-salinity (LS) and high-salinity (HS) conditions.

| Parameters | LS_day 0 | LS_day 100 | HS_day 0 | HS_day 100 |
| --- | --- | --- | --- | --- |
| AI_mod_ | 0.08 ± 0.33 | 0.1 ± 0.26 | 0.09 ± 0.26 | 0.07 ± 0.22 |
| DBE | 6.91 ± 3.05 | 7.18 ± 2.91 | 7.42 ± 3.11 | 7.14 ± 2.94 |
| NOSC | -0.34 ± 0.48 | -0.36 ± 0.4 | -0.35 ± 0.42 | -0.39 ± 0.37 |
| H/C | 1.42 ± 0.29 | 1.41 ± 0.26 | 1.42 ± 2.56 | 1.44 ± 0.24 |
| O/C | 0.48 ± 15 | 0.47 ± 0.13 | 0.48 ± 0.14 | 0.47 ± 0.16 |

Values represent the mean ± standard deviation of five molecular-level indices, including modified aromaticity index (AI_mod_), double bond equivalents (DBE), nominal oxidation state of carbon (NOSC), hydrogen-to-carbon ratio (H/C), and oxygen-to-carbon ratio (O/C), measured at the beginning and end of the 100-day incubation for LS and HS treatments.

**References**

1. Martin M. Cutadapt removes adapter sequences from high-throughput sequencing reads. *EMBnet journal* 2011;**17**:10-2 <https://doi.org/10.14806/ej.17.1.200>

2. Zhang J, Kobert K, Flouri T *et al.* Pear: A fast and accurate illumina paired-end read merger. *Bioinformatics* 2014;**30**:614-20 <https://doi.org/10.1093/bioinformatics/btt593>

3. Schmieder R, Edwards R. Quality control and preprocessing of metagenomic datasets. *Bioinformatics* 2011;**27**:863-4 <https://doi.org/10.1093/bioinformatics/btr026>

4. Edgar RC. Uparse: Highly accurate otu sequences from microbial amplicon reads. *Nat Methods* 2013;**10**:996-8 <https://doi.org/10.1038/nmeth.2604>

5. Edgar RC. Sintax: A simple non-bayesian taxonomy classifier for 16s and its sequences. *biorxiv* 2016:074161 <https://doi.org/10.1101/074161>

6. Ning D, Yuan M, Wu L *et al.* A quantitative framework reveals ecological drivers of grassland microbial community assembly in response to warming. *Nat Commun* 2020;**11**:4717 <https://doi.org/10.1038/s41467-020-18560-z>

7. Wen S, Hu A, Dini‐Andreote F *et al.* Molecular activity mediates the composition and assembly of dissolved organic matter in lake sediments. *Limnol Oceanogr* 2025;**70**:1196-209 <https://doi.org/10.1002/lno.70015>

8. Stedmon CA, Markager S, Bro R. Tracing dissolved organic matter in aquatic environments using a new approach to fluorescence spectroscopy. *Mar Chem* 2003;**82**:239-54 <https://doi.org/10.1016/s0304-4203(03)00072-0>

9. Williams CJ, Yamashita Y, Wilson HF *et al.* Unraveling the role of land use and microbial activity in shaping dissolved organic matter characteristics in stream ecosystems. *Limnol Oceanogr* 2010;**55**:1159-71 <https://doi.org/10.4319/lo.2010.55.3.1159>

10. Coble PG. Characterization of marine and terrestrial dom in seawater using excitation-emission matrix spectroscopy. *Mar Chem* 1996;**51**:325-46 <https://doi.org/10.1016/0304-4203(95)00062-3>

11. Murphy KR, Stedmon CA, Waite TD *et al.* Distinguishing between terrestrial and autochthonous organic matter sources in marine environments using fluorescence spectroscopy. *Mar Chem* 2008;**108**:40-58 <https://doi.org/10.1016/j.marchem.2007.10.003>

12. Van Geldermalsen M, Quek L-E, Turner N *et al.* Benzylserine inhibits breast cancer cell growth by disrupting intracellular amino acid homeostasis and triggering amino acid response pathways. *BMC Cancer* 2018;**18**:689 <https://doi.org/10.1186/s12885-018-4599-8>

13. May-Zhang LS, Yermalitsky V, Melchior JT *et al.* Modified sites and functional consequences of 4-oxo-2-nonenal adducts in hdl that are elevated in familial hypercholesterolemia. *J Biol Chem* 2019;**294**:19022-33 <https://doi.org/10.1074/jbc.RA119.009424>

14. Luo R, Wangqin R, Zhu L *et al.* Neuroprotective mechanisms of 3-n-butylphthalide in neurodegenerative diseases. *Biomed Rep* 2019;**11**:235-40 <https://doi.org/10.3892/br.2019.1246>
